# Reproducibility of radiomic features in CT images of NSCLC patients: an integrative analysis on the impact of acquisition and reconstruction parameters

**DOI:** 10.1186/s41747-021-00258-6

**Published:** 2022-01-25

**Authors:** Lisa Rinaldi, Simone P. De Angelis, Sara Raimondi, Stefania Rizzo, Cristiana Fanciullo, Cristiano Rampinelli, Manuel Mariani, Alessandro Lascialfari, Marta Cremonesi, Roberto Orecchia, Daniela Origgi, Francesca Botta

**Affiliations:** 1 Radiation Research Unit, IEO European Institute of Oncology IRCCS, via Ripamonti 435, 20141 Milan, Italy; 2 Department of Physics, Università degli Studi di Pavia and INFN, via Bassi 6, 27100 Pavia, Italy; 3 Department of Experimental Oncology, IEO European Institute of Oncology IRCCS, via Ripamonti 435, 20141 Milan, Italy; 4 Clinica di Radiologia EOC, Istituto Imaging della Svizzera Italiana (IIMSI), via Tesserete 46, 6900 Lugano, Switzerland; 5 Università della Svizzera italiana, via G.Buffi 13, 6900 Lugano, Switzerland; 6 Postgraduate School of Diagnostic and Interventional Radiology, Università degli Studi di Milano, Via Festa del Perdono 7, 20122 Milan, Italy; 7 Department of Radiology, IEO European Institute of Oncology IRCCS, via Ripamonti 435, 20141 Milan, Italy; 8 Scientific Directorate, IEO European Institute of Oncology IRCCS, via Ripamonti 435, 20141 Milan, Italy; 9 Medical Physics Unit, IEO European Institute of Oncology IRCCS, via Ripamonti 435, 20141 Milan, Italy

**Keywords:** Carcinoma (non-small-cell lung), Image processing (computer-assisted), Machine learning, Reproducibility of results, Tomography (x-ray computed)

## Abstract

**Background:**

We investigated to what extent tube voltage, scanner model, and reconstruction algorithm affect radiomic feature reproducibility in a single-institution retrospective database of computed tomography images of non-small-cell lung cancer patients.

**Methods:**

This study was approved by the Institutional Review Board (UID 2412). Images of 103 patients were considered, being acquired on either among two scanners, at 100 or 120 kVp. For each patient, images were reconstructed with six iterative blending levels, and 1414 features were extracted from each reconstruction. At univariate analysis, Wilcoxon-Mann-Whitney test was applied to evaluate feature differences within scanners and voltages, whereas the impact of the reconstruction was established with the overall concordance correlation coefficient (OCCC). A multivariable mixed model was also applied to investigate the independent contribution of each acquisition/reconstruction parameter. Univariate and multivariable analyses were combined to analyse feature behaviour.

**Results:**

Scanner model and voltage did not affect features significantly. The reconstruction blending level showed a significant impact at both univariate analysis (154/1414 features yielding an OCCC < 0.85) and multivariable analysis, with most features (1042/1414) revealing a systematic trend with the blending level (multiple comparisons adjusted *p* < 0.05). Reproducibility increased in association to image processing with smooth filters, nonetheless specific investigation in relation to clinical endpoints should be performed to ensure that textural information is not removed.

**Conclusions:**

Combining univariate and multivariable models is allowed to identify features for which corrections may be applied to reduce the trend with the algorithm and increase reproducibility. Subsequent clustering may be applied to eliminate residual redundancy.

**Supplementary Information:**

The online version contains supplementary material available at 10.1186/s41747-021-00258-6.

## Key points

- Scanner and voltage did not affect features significantly.
- Reconstruction algorithm blending levels impact on the reproducibility of features.
- Combination of multiple analyses may help to characterise feature behaviour.

## Background

Current clinical practice relies on radiological imaging for the diagnosis, evaluation, and monitoring of diseases. Radiomics is an emerging discipline which aims to add further quantitative objectivity to the visual interpretation of the medical images performed by the physicians [1, 2]. A huge variety of mathematical descriptors, named *radiomic features*, can be calculated from images quantifying different aspects of the tumour shape and texture. Radiomics investigates the ability of some of such features to characterise clinical properties of the lesions [3]. Under the assumption that the features might capture relevant information not discriminated by the human eye [4], radiomics attempts to become a precious tool to support personalised clinical decisions [5].

Importantly, radiomics can be applied successfully in the clinical practice only if the radiomic-based predictive models are robust and generalisable. To this aim, radiomic features must not be biased by any variation in the image signal except for that ascribable to actual biological processes [6, 7]. Conversely, it was extensively observed that the use of different parameters during acquisition and post-acquisition may modify the image signal with a significant impact on the value of the radiomic features, even when the diagnostic quality of the image is maintained [8–11].

Quite often image databases collected for radiomic research are highly heterogeneous, including images obtained with different scanners, acquisition protocols and post-acquisition techniques [1, 12, 13]. Among others, a recent study from our group [14] confirmed the possible confounding factor of reconstruction algorithms. In this study, we built a predictive model, identifying a possible association of the radiomic and clinical information with the lymph node status and the overall survival in 270 patients with lung cancer. Through an analysis of the variance on 422 selected radiomic features, we found that 254 of them differed significantly between the two types of reconstruction algorithm: iterative reconstruction (IR) and filtered backprojection (FBP). In these cases, it is fundamental to take the reproducibility issue into account, by either disregarding or properly correcting and harmonising the features significantly affected by the different imaging procedures [15–17].

Focusing on computed tomography (CT), previous studies investigated the radiomic features variability related to different acquisition parameters (tube current [18], slice thickness [19–22] and tube voltage peak [18]), reconstruction techniques [19, 20, 22–26], segmentation of the volume of interest [27–29], and post-processing techniques [30–33]. Quite often, however, the list of reproducible features obtained in one study is not fully provided or is not directly exportable to a different database of images, if obtained with different equipment or image settings.

In this paper, we faced the reproducibility issue in a retrospective database of CT images available at our Institute for patients affected by non-small-cell lung cancer (NSCLC). We aimed to contribute to the existing literature first by identifying the list of reproducible features for radiomic analysis of NSCLC CT studies, and, most importantly, by suggesting an integration of different metrics, which can improve the interpretation of reproducibility results and can be replicated in other scenarios.

## Methods

### Patients and ethics issues

Patients undergoing diagnostic chest contrast-enhanced CT imaging at our Institute between January 2019 and December 2019 were retrospectively selected. Inclusion criteria were the availability of CT raw-data, CT being acquired with the institutional standard protocol and with beam energy set to either 100 or 120 kVp, and histologically proven diagnosis of NSCLC. Exclusion criteria were tumour volume smaller than 5 cm^3^ or larger than 200 cm^3^. The Institutional Review Board approved the study (UID 2412) waiving the need for informed consent.

### Image acquisition and reconstruction

Contrast-enhanced CT images were acquired using either Discovery CT750 HD or Optima CT660 scanner (General Electric Healthcare, Wisconsin, USA) according to the current institutional standard protocol (acquisition: helical acquisition, 2.5 mm slice thickness and spacing, automatic tube current modulation, tube voltage set to 100, 120 or 140 kVp according to patient body mass index, noise index suitably optimised for each voltage to provide comparable image quality; reconstruction with standard convolution kernel, adaptive statistical iterative reconstruction (ASIR) algorithm with 60% blending level on Discovery CT750 HD and 50% blending level on Optima CT660). At our institute, two types of iodinated-contrast medium are usually injected, Visipaque® 320 (General Electric Healthcare, Wisconsin, USA) or Ultravist® 370 (Bayer Healthcare, Leverkusen, Germany), and the volume of the contrast medium is selected depending on contrast medium concentration and patient weight.

Previously, during the optimisation process, different blending levels were tested and appear in our retrospective databases. To replicate and investigate this variability, the CT images (portal phase series) of the patients included in this study were reconstructed applying each time a different IR blending level: 0% (equivalent to FBP), 20%, 40%, 50%, 60% and 80% (referred to as IR20, IR40, IR50, IR60 and IR80, respectively).

### Tumour segmentation

One pulmonary lesion for each patient was contoured manually slice by slice on the series used for clinical reporting (AWServer 3.2 Ext. 2.0 tool, General Electric Healthcare, Wisconsin, USA). The so obtained volume of interest was used for the radiomic analysis of all the six reconstructions, inherently co-registered. This allowed us to investigate the impact of reconstruction algorithms avoiding possible biases that might have occurred if the segmentation was repeated separately on each reconstructed image.

Tumours were contoured by three operators with similar experience (more than 7 years of experience) after agreement on segmentation criteria and settings. Radiologists trained among each other to reach a consensus on the segmentation procedure, including window setting for visualisation (1500 Hounsfield units, HU, width and − 600 HU level for lung window, 350 HU width and 40 HU level for mediastinal window, depending on lesion localisation), exclusion of the vessels, and inclusion of opacity on the lesion edge.

#### Radiomic feature extraction

The radiomic features were computed through the open-source package Pyradiomics v. 2.2.0 [34], from each of the six reconstructed images for each patient. Radiomic features were extracted considering the following seven categories: shape; first order; grey level co-occurrence matrix; grey level run length matrix; grey level size zone matrix; neighbouring grey tone difference matrix; and grey level dependence matrix.

According to IBSI recommendations [35], before feature computation image resampling in the axial plane (Pyradiomics B-Spline interpolator, ‘sitkBSpline’ [36, 37]) and voxel intensity discretisation (25 HU fixed bin [7, 34, 38, 39]) were applied.

*Shape*, intensity (*first order*) and *texture* features were calculated, both from original images without filtering and after applying the wavelet filter (order 1 Coiflet, Pyradiomics default [1, 7, 34, 40]) and Laplacian of Gaussian (LoG) filter with different values of Gaussian standard deviation (*sigma*: 0.5, 1.0, 1.5, 2.5 and 5.0 mm [20, 34, 41–43]). The names of the features will be presented with the suffix “original”, “Wavelet” or “LoG”, followed by the feature category and the feature name. Additional details on extracted feature categories and parameters set for calculation are reported in Supplementary Methods.

Features from the s*hape* category will be included only in the “original” group since they are identical for original and filtered images.

### Statistical analysis

Clinical similarity between patient and tumour characteristics (age, volume, gender, side, position, tumour type, previous therapy and pTNM stage), according to scanner and tube voltage, was evaluated with *χ*^2^ or Fisher exact test for categorical variables, and with Wilcoxon-Mann-Whitney test for continuous variables.

Univariate analysis to evaluate differences in feature values within CT scanners (Optima CT660 versus Discovery CT750 HD) and within tube voltages (100 versus 120 kVp) was performed with Wilcoxon-Mann-Whitney test using feature values obtained from FBP and IR60 images (this latter chosen as representative, being in the middle of the IR blending level interval investigated).

The overall concordance between the six different settings for the reconstruction algorithm on the same patient was evaluated for each feature with the overall concordance correlation coefficient (OCCC) [44]. An OCCC threshold equal to 0.85 was used to classify features affected (OCCC < 0.85) or not (OCCC ≥ 0.85) by the IR blending level applied during reconstruction [20, 45, 46].

Additionally, to investigate the independent contribution of each acquisition and reconstruction parameter on feature variation, a multivariable mixed model was used, including subjects as random effect to take into account within-subject variation for the six reconstructions, and adjusting by clinical volume. For this analysis, FBP was set as reference category, and one model coefficient and *p* value for each IR blending level was calculated for comparison with FBP. All *p* values were corrected with the false discovery rate (FDR) method [47] to properly account for multiple testing; adjusted *p* values < 0.05 were considered statistically significant.

For a deeper understanding of feature dependence on reconstruction algorithm settings, features were classified in four groups.

*Group 1*, with OCCC ≥ 0.85 and mixed model FDR-adjusted *p* value < 0.05. Over-threshold OCCC indicates that feature variations among the different reconstruction settings for each patient are small in comparison to the variations observed in the entire dataset (differences among patients). The significant *p* value of the multivariable mixed model indicates that such small variations follow systematically the same trend for almost all the patients in the dataset. Hence, features belonging to this category change slightly when modifying the reconstruction setting, and the trend of such variation can be predicted and corrected, since it is similar for almost all patients.

*Group 2*, with OCCC ≥ 0.85 and mixed model FDR-adjusted *p* value ≥ 0.05. When changing the reconstruction setting, the features vary slightly (or do not vary at all if OCCC = 1) in comparison to the whole dataset variations, but the non-significant *p* value of the mixed model indicates that the trend of such variation (if any) is not systematic among patients, but random.

*Group 3*, with OCCC < 0.85 and mixed model FDR-adjusted *p* value < 0.05. The low OCCC value indicates that the feature variation when changing the reconstruction setting is not negligible in comparison to the variations observed in the whole dataset. The trend of such variations is systematically the same for almost all patients.

Group 4, with OCCC < 0.85 and mixed model FDR-adjusted *p* value ≥ 0.05. The features exhibit a relevant variation in comparison to the variations observed in the whole dataset, but the sign and entity of such variations change randomly among patients.

As sensitivity analysis, pair differences were calculated with Wilcoxon signed rank-test to compare algorithms among them (not necessarily with FBP as reference).

The whole analysis was also performed on a subgroup of IR blending levels (IR40, IR50, IR60 and IR80), taking the IR40 as a reference, in order to provide results also in a setting more representative of the current clinical applications.

Finally, the features extracted from original images were clustered according to a minimum intra-cluster correlation criterion (Spearman’s |ρ| ≥ 0.75) to quantify feature redundancy.

All analyses were performed with R (v. 4.0.0) [48], and tests were two-sided.

## Results

Among 163 patients selected for the availability of CT raw-data, 103 (59 men, mean age 71 years; 44 women, mean age 67 years) fulfilled the remaining enrolment criteria and were included in the study: 50 (49%) imaged on Optima CT660 scanner (50% at 100 kVp, 50% at 120 kVp); 53 (51%) imaged on Discovery CT750 HD scanner (51% at 100 kVp, 49% at 120 kVp), resulting in four populations according to scanner and tube voltage (Figure S1).

The baseline clinical characteristics are summarised in Table 1, as long as the *p* values for the comparison of clinical characteristics between the two scanners and the two tube voltage patient populations. No statistically significant difference was found, confirming clinical similarity of the four populations. For the subgroup of patients with available information on pTNM stage and grading, no statistically significant difference was observed among the populations (results not shown).

**Table 1:** Baseline characteristics of the study population.

Variables	Overall cohort (*n* = 103)	Scanner Optima CT660 (*n* = 50)	Scanner DiscoveryCT750 HD (*n* = 53)	***p*** value (scanner)	Tube voltage 120 kVp (*n* = 51)	Tube voltage 100 kVp (*n* = 52)	***p*** value (kVp)
**Gender**							
Male Female	59 (57%) 44 (43%)	28 (56%) 22 (44%)	31 (58%) 22 (42%)	0.798^a^	33 (65%) 18 (35%)	26 (50%) 26 (50%)	0.131^a^
**Age**							
Mean (median) IQR	69.2 (70) (64–75)	69.4 (70) (65–75.3)	68.9 (69) (62–74.5)	0.498^c^	69.8 (70) (64–76)	68.6 (68.5) (62–74.8)	0.251^c^
**Side** Right Left	60 (58%) 43 (42%)	31 (62%) 19 (38%)	29 (55%) 24 (45%)	0.454^a^	31 (61%) 20 (39%)	29 (56%) 23 (44%)	0.606^a^
**Position** Upper Medium Lower Mixed	63 (64%) 1 (1%) 29 (30%) 5 (5%)	33 (69%) 1 (2%) 13 (27%) 1 (2%)	30 (60%) 0 (0%) 16 (32%) 4 (8%)	0.360^b^	30 (61%) 1 (2%) 16 (33%) 2 (4%)	33 (67%) 0 (0%) 13 (27%) 3 (6%)	0.731^b^
**Volume (cm**^**3**^**)**							
Mean (median) IQR	46.4 (39.1) (19.1–62.8)	44.2 (40.6) (19–54.7)	48.5 (38.1) (19.5–71.9)	0.843^c^	52.1 (42) (20.7–67.9)	40.9 (36.7) (18.4–56.2)	0.181^c^
**Histological type**							
Adenocarcinoma Squamous cell carcinoma Neuroendocrine	83 (82%) 16 (16%) 2 (2%)	38 (78%) 10 (20%) 1 (2%)	45 (87%) 6 (11%) 1 (2%)	0.580^b^	40 (78%) 9 (18%) 2 (4%)	43 (86%) 7 (14%) 0 (0%)	0.380^b^
**Previous therapy**							
No Yes	75 (74%) 26 (26%)	38 (76%) 12 (24%)	37 (73%) 14 (27%)	0.692^a^	33 (66%) 17 (34%)	42 (82%) 9 (18%)	0.060^a^
**Scanner**							
Optima CT660 Discovery CT750 HD	50 (49%) 53 (51%)	–	–	–	25 (49%) 26 (51%)	25 (48%) 27 (52%)	0.924^a^
**Tube voltage (kVp)**							
120 100	51 (50%) 52 (50%)	25 (50%) 25 (50%)	26 (49%) 27 (51%)	0.924^a^	–	–	–

^a^*χ*^2^ test

^b^Fisher’s exact test

^c^Wilcoxon-Mann-Whitney test. Missing data: histological type (*n* = 2); previous therapy (*n* = 2); position (*n* = 5). *IQR* Interquartile range

A total of 1414 radiomic features were extracted, including 154 from original images (14 *shape*, 17 *first order* and 123 *texture* features), 560 from wavelet-filtered images (68 *first order* and 492 *texture*) and 700 from LoG-filtered images (85 *first order* and 615 *texture*). The full feature list is reported in Table S1 along with the 33 groups in which the original image features were clustered.

### Scanner model and tube voltage

Forty-four features were significantly different according to scanner and/or tube voltage, either at univariate or multivariable (mixed model) analysis or both (Table S2). Focusing on multivariable analysis, only 5 features (1 from *shape* category and 4 from *texture* category and wavelet-filtered images) showed significant dependence on tube voltage, and 1 (*shape_SurfaceArea*) on scanner (Table 2).

**Table 2:** FDR-adjusted *p* values for univariate and multivariable analysis for the effect of scanner and tube voltage

Features	Scanner (univar) FBP	Scanner (univar) IR60	Tube voltage (univar) FBP	Tube voltage (univar) IR60	Scanner (mixed)	Tube voltage (mixed)
shape_SurfaceArea	0.897	0.960	0.735	0.695	**0.027**	0.886
shape_VoxelVolume	0.936	0.960	0.784	0.695	0.190°	**< 0.001°**
Wavelet-glszm_SizeZoneNonUniformityNormalized*	0.264	0.905	**0.005**	**0.016**	0.996	**0.005**
Wavelet-glszm_SmallAreaEmphasis*	0.264	0.905	**0.006**	**0.016**	0.996	**0.005**
Wavelet-glcm1_Correlation*	0.462	0.905	0.144	0.130	0.561	**0.018**
Wavelet-glcm1_InverseVariance*	0.231	0.905	**0.004**	0.097	0.309	**0.012**

Only the features with significant FDR-adjusted *p* values at multivariate analysis

*HH filter

°In the model with VoxelVolume as the dependent variable, clinical volume was not used as independent predictor. *FBP* filtered backprojection, *FDR* false discovery rate, *IR* iterative reconstruction

### Reconstruction algorithm

**Fig. 1:**
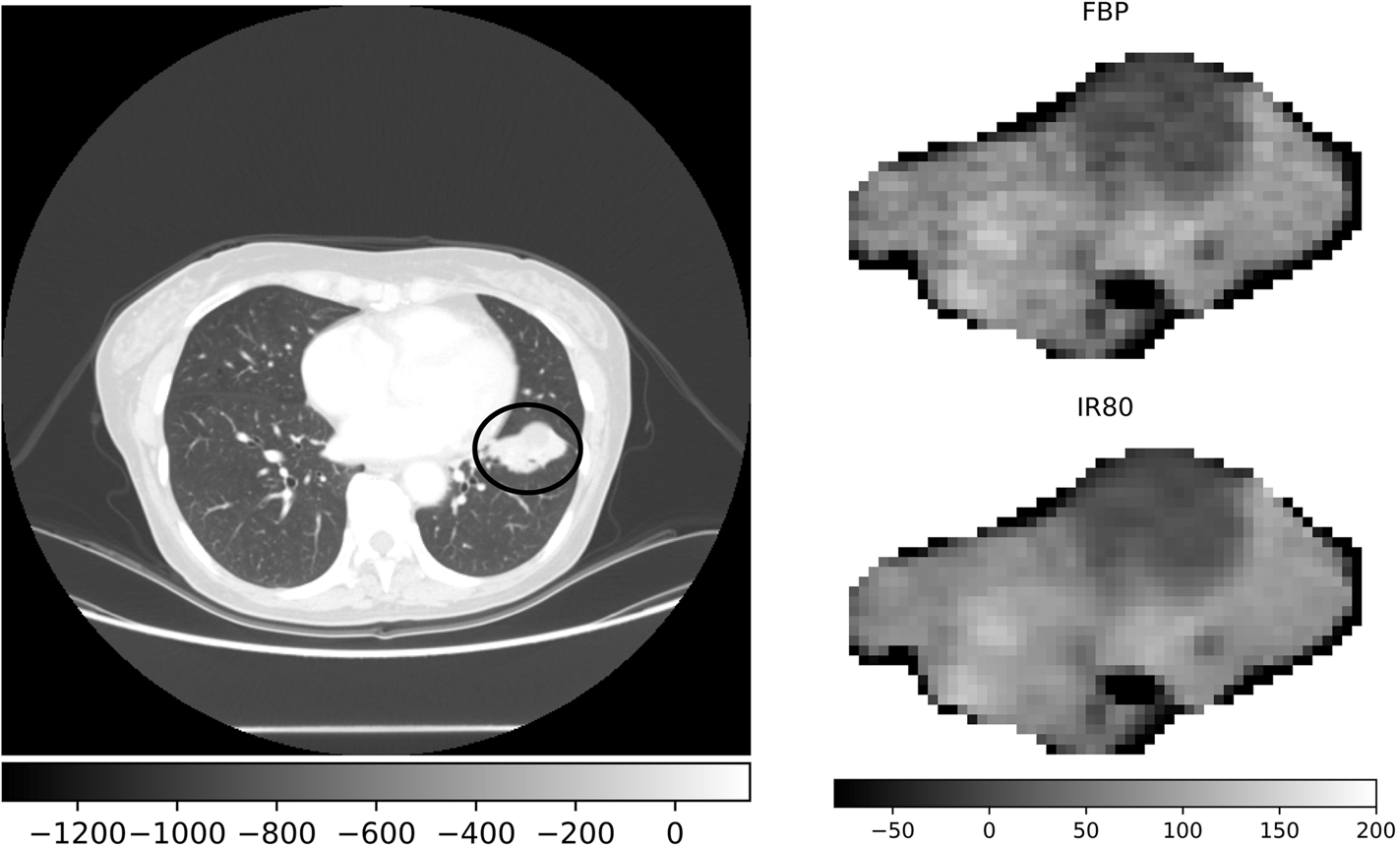
Visual comparison of computed tomography images of the same patient reconstructed with two blending levels. The image on the left shows the thorax of the patient with the encircled lesion, displayed using the lung window. The same lesion is isolated in the right figures, displaying the filtered backprojection (FBP) reconstruction and the iterative algorithm with ASIR 80% (IR80) with a mediastinal window

In order to evidence the impact of the reconstruction algorithm on the image texture, we reported an example of two reconstructions (FBP and IR80) of the same lesion (Fig. 1).

**Fig. 2:**
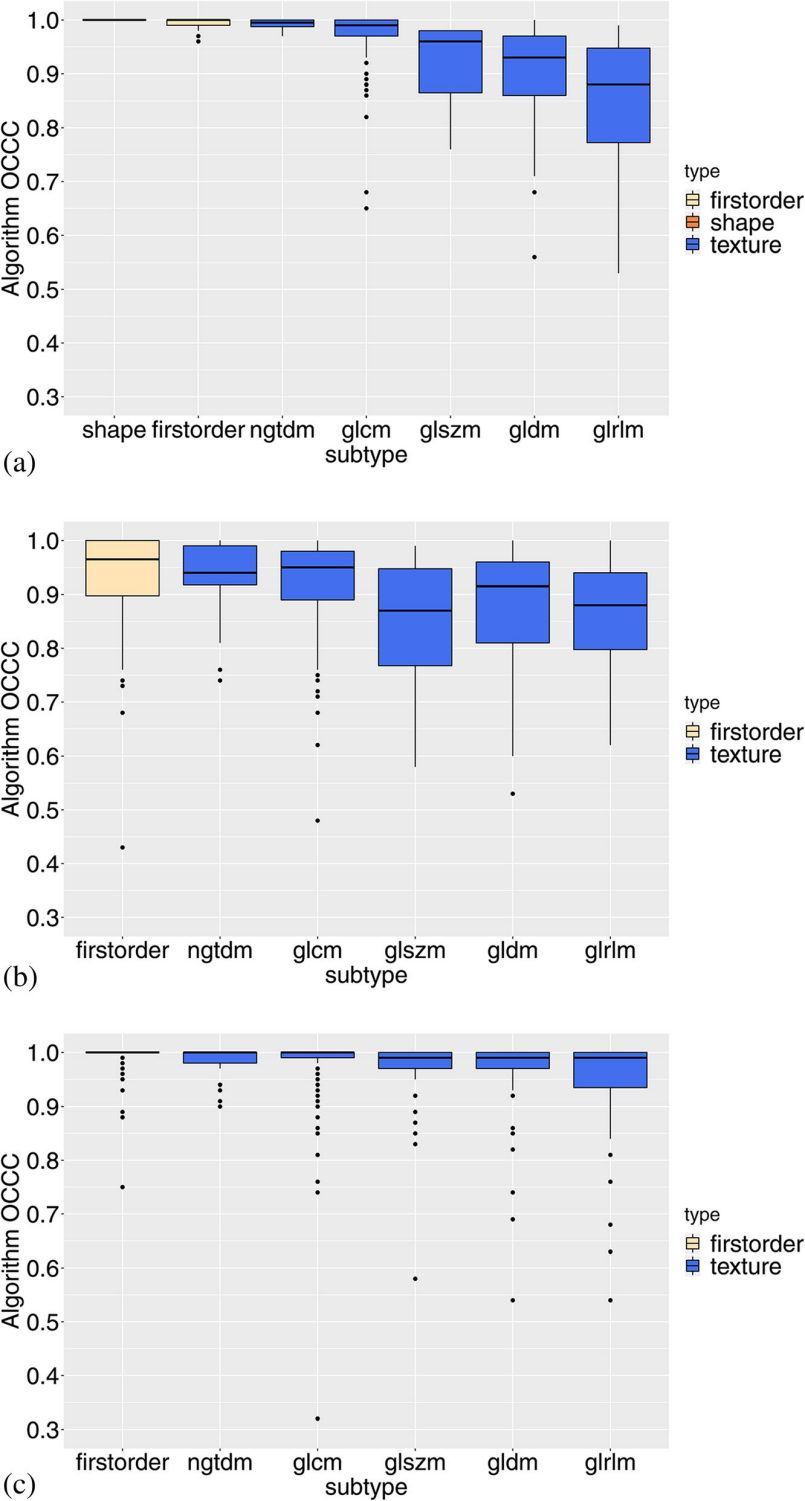
Overall concordance correlation coefficient (OCCC) among the different reconstruction algorithms. The OCCC is plotted within each subtype of feature and for feature extracted from the original images (**a**), and the wavelet- (**b**) and LoG-filtered (**c**) images

From the concordance analysis between the different reconstruction settings, we obtained that 16/154 features (10%) had small reproducibility (OCCC < 0.85), all in *texture* categories, in case of features from original images (Fig. 2a). Features from *grey level run length matrix* category were mostly affected by reconstruction algorithm setting. In case of wavelet-filtered images (Fig. 2b), 116/560 features (21%) yielded OCCC < 0.85, mostly (51%) from the HH-wavelet group, whereas LL-wavelet features exhibited the highest concordance. Features from LoG-filtered images showed the highest reproducibility in all feature categories and for each value of sigma parameter (Fig. 2c): only 22/700 features (3%) yielded OCCC < 0.85. The analogous results obtained when restricting the analysis to the IR40-IR80 range are reported in Figure S2, with 6/154 (4%), 22/560 (4%) and 6/700 (1%) features yielding OCCC < 0.85 for the original, wavelet and LoG features, respectively. Table 3 reports the median OCCC for each image type and feature category, both for the main analysis and, in parentheses, for the subanalysis restricted to the IR40-IR80 range. Full results (OCCC value obtained for each feature) are reported in Tables S3, S4 and S5 along with the FDR-adjusted *p* values obtained from the multivariable mixed model.

**Table 3:** Median OCCC values calculated for each image type and feature category

Image type	Image subtype	Feature category						
		Shape	First order	ngtdm	glcm	glszm	gldm	glrlm
**Original**	**All**	**1.00 (1.00)**	**1.00 (1.00)**	**1.00 (1.00)**	**0.99 (1.00)**	**0.96 (0.98)**	**0.93 (0.97)**	**0.88 (0.95)**
**Wavelet**	**All**	**–**	**0.97 (0.99)**	**0.94 (0.97)**	**0.95 (0.98)**	**0.87 (0.94)**	**0.92 (0.95)**	**0.88 (0.95)**
	LH	–	0.93 (0.98)	0.94 (0.97)	0.93 (0.98)	0.80 (0.93)	0.89 (0.94)	0.85 (0.94)
	HL	–	0.96 (0.98)	0.94 (0.98)	0.96 (0.99)	0.88 (0.94)	0.90 (0.95)	0.88 (0.95)
	HH	–	0.88 (0.96)	0.84 (0.92)	0.89 (0.95)	0.81 (0.92)	0.88 (0.95)	0.87 (0.94)
	LL	–	1.00 (1.00)	1.00 (1.00)	0.99 (1.00)	0.97 (0.99)	0.96 (0.99)	0.94 (0.97)
**LoG**	**All**	**–**	**1.00 (1.00)**	**1.00 (1.00)**	**1.00 (1.00)**	**0.99 (1.00)**	**0.99 (1.00)**	**0.99 (1.00)**
	0.5 mm	–	0.97 (0.99)	0.96 (0.98)	0.96 (0.99)	0.87 (0.95)	0.86 (0.93)	0.85 (0.93)
	1.0 mm	–	1.00 (1.00)	0.98 (0.99)	0.99 (1.00)	0.98 (0.99)	0.98 (0.99)	0.97 (0.98)
	1.5 mm	–	1.00 (1.00)	0.99 (1.00)	1.00 (1.00)	0.99 (1.00)	1.00 (1.00)	0.99 (1.00)
	2.5 mm	–	1.00 (1.00)	1.00 (1.00)	1.00 (1.00)	1.00 (1.00)	1.00 (1.00)	1.00 (1.00)
	5.0 mm	–	1.00 (1.00)	1.00 (1.00)	1.00 (1.00)	1.00 (1.00)	1.00 (1.00)	1.00 (1.00)

In parentheses, the results obtained when restricting the analysis to the settings most used in clinics (IR40, IR50, IR60 and IR80). *IR* iterative reconstruction, *glcm* grey level co-occurrence matrix, *gldm* grey level dependence matrix, *glrlm* grey level run length matrix, *glszm* grey level size zone matrix, *ngtdm* neighbouring grey tone difference matrix, *LoG* Laplacian of Gaussian, OCCC overall concordance correlation coefficient

According to the multivariable mixed model, 110/140 (78.5%) features from original images (*shape* features excluded), 462/560 (82.5%) from wavelet-filtered images and 470/700 (67%) from LoG-filtered images were significantly affected by IR setting (mixed model FDR-adjusted *p* value < 0.05). Similar results were obtained for the subanalysis restricted to the IR40-IR80 range: 112/140 (80%) for original images, 457/560 (82%) for wavelet-filtered images and 421/700 (60%) for LoG-filtered images.

**Fig. 3:**
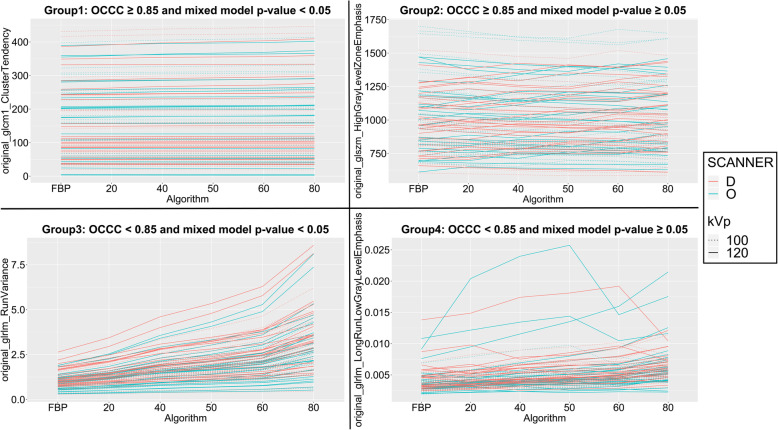
Feature variation according to reconstruction algorithm, scanner and tube voltage parameters, for all the investigated patients. One feature is selected as representative for each of the four groups of features identified, according to the overall concordance correlation coefficient (OCCC) and multivariable analysis results

We combined the results of the two metrics adopted for the reproducibility analysis and divided the features in four groups, as described in the “Methods” section (“Statistical analysis” section). One representative feature for each group was selected and displayed in Fig. 3 to highlight the different behaviours of the features falling in the different groups. To this aim, we plotted the absolute value of these features when increasing the reconstruction blending levels for the four patient populations, each line representing a different patient.

**Fig. 4:**
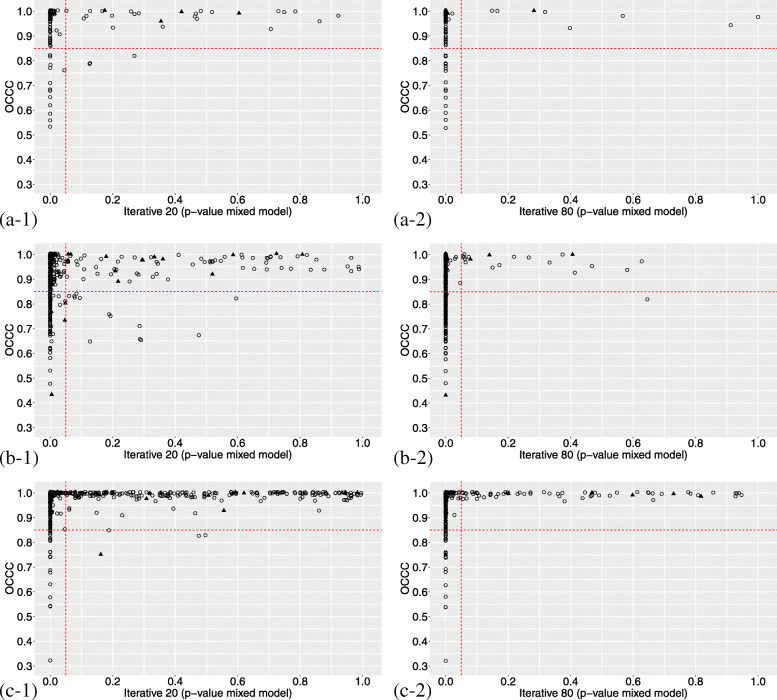
Comparison between the overall concordance correlation coefficient (OCCC) and the *p* value from the multivariable mixed models for reconstruction algorithm analysis. The plots report two analysed cases of iterative reconstruction (IR20 and IR80), as an example, for original, Wavelet and LoG features: original-IR20 (**a-1**), original-IR80 (**a-2**), Wavelet-IR20 (**b-1**), Wavelet-IR80 (**b-2**), LoG-IR20 (**c-1**) and LoG-IR80 (**c-2**). The triangles indicate *first order* features, while the circles stand for *texture* features. The red dotted lines divide the plots in the four parts, according to the threshold chosen for OCCC and *p* value, equal to 0.85 and 0.05, respectively

We found that the majority of the features fall in group 1 (OCCC ≥ 0.85 and *p* value < 0.05), suggesting the capability of the features to capture the gradual smoothing effect of the increasing IR strength on the image texture, with a similar trend for all the patients. In contrast, group 4 is the less populated. In Fig. 4, we reported some examples of these findings, by plotting the OCCC value versus the mixed model FDR-adjusted *p* value (each point in the graph representing a feature) for six cases: original (Fig. 4a), wavelet-filtered (Fig. 4b) and LoG-filtered (Fig. 4c) images, in each case including the two extreme configurations of IR blending level (IR20 and IR80) versus FBP. The red lines divide each plot in four quadrants, corresponding to the four groups described in the “Methods” section (“Statistical analysis” section). The percentage of features falling in each group for the six cases is reported in Table 4, whereas Table S6 reports the corresponding results for the subanalysis (IR50 and IR80 versus IR40).

**Table 4:** Percentage of features falling in each of the four groups

Image type	Image subtype	Group 1		Group 2		Group 3		Group 4	
		IR20	IR80	IR20	IR80	IR20	IR80	IR20	IR80
**Original**	**All** (shape excluded)	**70.0**	**82.9**	**18.6**	**5.7**	**9.3**	**11.4**	**2.1**	**0.0**
**Wavelet**	**All**	**65.0**	**75.9**	**14.3**	**3.4**	**18.4**	**20.5**	**2.3**	**0.2**
	LH	16.8	19.3	2.9	0.4	4.6	5.3	0.7	0.0
	HL	18.0	20.7	4.1	1.4	2.7	2.9	0.2	0.0
	HH	10.9	13.9	3.6	0.5	9.8	10.3	0.7	0.2
	LL	19.3	22.0	3.7	1.1	1.3	2.0	0.7	0.0
**LoG**	**All**	**64.6**	**87.6**	**32.3**	**9.3**	**2.7**	**3.1**	**0.4**	**0.0**
	0.5 mm	13.4	16.0	3.6	1.0	2.6	3.0	0.4	0.0
	1.0 mm	15.5	19.0	4.4	0.9	0.1	0.1	0.0	0.0
	1.5 mm	13.1	18.4	6.9	1.6	0.0	0.0	0.0	0.0
	2.5 mm	11.6	17.6	8.4	2.4	0.0	0.0	0.0	0.0
	5.0 mm	11.0	16.6	9.0	3.4	0.0	0.0	0.0	0.0

The results are reported for the IR20 and the IR80 reconstructions. The percentage for the original images is evaluated excluding the *shape* features. *IR* iterative reconstruction, *LoG* Laplacian of Gaussian

The pair comparisons among reconstruction settings are reported in Figure S3 for the features obtained from original images. The number of features with significantly different values between two algorithms ranges from 117 (84%, excluding *shape* category) to 128 (91%), with the number of poorly reproducible features increasing when increasing the IR blending level interval (considering FBP as more similar to IR20).

## Discussion

The main findings of this study are related to the influence of reconstruction setting on the value of radiomic features, and its interpretation. Our findings in relation to the dependence on scanner and tube voltage (not statistically significant in our sample) basically confirm previous results [18, 49–51].

Besides confirming that the IR blending level has a significant impact on the value of a set of features extracted from CT images of patients affected by NSCLC [20, 23, 24, 52], we provided feature-by-feature results which might be conveniently compared with similar findings obtained on different dataset (images of clinically comparable cases obtained at different Institutes, with different scanner models, acquisition and reconstruction settings) to verify if the subset of reproducible radiomic features is coherent among different samples.

In addition, we introduced a novel approach to investigate and handle the dependency of each feature value on the reconstruction setting. By joining two different statistical analyses (concordance analysis and multivariable mixed model), we showed how radiomic features can be classified in four different groups exhibiting different behaviour in relation to the reconstruction settings, which might require different selection or correction strategies to guarantee robustness and reproducibility of radiomic results. We believe that such combined approach is useful to provide more complete information as compared to the use of one model alone, and it might allow a more comprehensive handling of the reproducibility issue.

Indeed, if considering the results of univariate concordance analysis alone, 1260/1414 features (89%) exhibiting OCCC ≥ 0.85 would be considered as reproducible and usable, without additional correction, for a radiomic analysis performed on a clinical database with similar characteristics as the one considered here. Conversely, the remaining 154/1414 features (11%) exhibiting OCCC < 0.85 would be excluded. Nonetheless, the subset of such excluded features falling in group 3 (OCCC < 0.85 and mixed model FDR-adjusted *p* value < 0.05) could be retrieved and included back in the analysis after properly accounting for the fact that their dependence on reconstruction setting is systematic among patients. The parameters needed to apply such correction are given as output by the multivariable model itself. To provide an example, we applied such correction to a feature belonging to group 3, *original_glrlm_RunVariance*, and compared the values obtained for different reconstruction settings before and after the correction (Figure S4). The systematic trend observed when varying the reconstruction setting is reduced, which might allow to retain the feature (and its potential informative content) for the radiomic analysis.

Similarly, among the features exhibiting OCCC ≥ 0.85, the subgroup falling in group 1 (OCCC ≥ 0.85 and mixed model FDR-adjusted *p* value < 0.05) might require a correction before being considered reproducible. The real necessity of such correction might depend on the clinical question that the radiomic analysis is supposed to answer. For example, if the aim is to discriminate two patient populations for which the difference—in terms of radiomic features—exists but is very small, even the slight feature variation introduced by different IR blending levels may have a relevant impact, confounding the data and impairing the ability of radiomics to reach its goal. In this case, the feature correction should be applied, similarly for features in group 3, despite the OCCC ≥ 0.85 would suggest feature reproducibility. If instead the difference between the features of two populations is far larger than the fluctuations due to the different reconstruction settings, it might be irrelevant to perform the correction or not. We plan to investigate these aspects in future studies for different clinical endpoints on the NSCLC population.

It must be noted that, in our sample the features falling in the above cited groups 1 and 3 are the vast majority, with highest prevalence in group 1 as compared to group 3 (Fig. 4 and Table 4). These features are the ones for which a trend among reconstruction blending levels has been observed and an appropriate correction may be thus applied to take into account these differences. This is of importance, because it suggests that possible differences in radiomic features according to different blending levels may be properly corrected for the majority of the features, thus avoiding discharging them from further statistical analyses. A similar behaviour was identified by Prezzi et al., analysing CT images of 28 patients with primary colorectal cancer with multilevel linear regression [25]. They studied the impact of the reconstruction strength by applying an ASIR algorithm in steps of 20% from 0 to 100%, in a controlled acquisition setting. Similar to our results, they found that the majority of the features extracted from original images had a systematic trend (increasing or decreasing linear behaviour) with the reconstruction strength. The features belonging to group 2 (OCCC ≥ 0.85 and *p* value ≥ 0.05) for all the IR blending levels can be definitively considered reproducible without need of any correction for any clinical endpoint, but in our sample their number is very small: 7 features extracted from the original images, 11 from the wavelet-filtered images and 55 from the LoG-filtered images.

Lastly, the features in group 4 (OCCC < 0.85 and *p* value ≥ 0.05) should be rejected without possibility of correction. In our sample, however, this group was poorly populated.

It should be highlighted that the data discussed so far refer to a heterogeneous database including all the six reconstruction settings from FBP to IR80. The full data reported in Tables S3, S4, S5 allow to derive conclusions for database including only FBP and a subset of the IR blending levels here considered, and the results of our subanalysis (including only IR40, IR50, IR60 and IR80) can be taken as reference for the current clinical scenario where FBP is progressively replaced by iterative algorithms. As expectable, the results of such subanalysis are quite similar to the ones of the main analysis, but with a general increase in feature reproducibility.

In addition, the result of the comparison of pairwise reconstruction algorithms for the original images (Figure S3) shows that if we changed the reference IR blending level in the multivariable model, the number of features whose value changes significantly between two IR blending levels would not vary considerably; hence, the above considerations hold valid independently on this choice. It is also important to note that, even after applying the feature selection and correction described so far, the number of reproducible features is likely to be very high, but many of them are highly correlated, so their number would be further reduced by clustering procedures (Table S1).

As possible limitations of the present study, we acknowledge the relatively small number of patients, the impossibility to investigate radiomic feature repeatability, the lack of an external validation of our results, and the inability to specifically account for the possible effect of segmentation performed by different operators. In this study, the segmentation by multiple operators should have had a negligible impact when focusing on the effect of reconstruction algorithm intra-patient, since in this case the region of interest was fixed across the different reconstruction settings. However, it might have slightly affected the assessment of inter-patients’ behaviour and the analysis on scanner and tube voltage dependence. An interesting future alternative may be applying automatic approach based on deep learning. Regarding repeatability, we plan to account for this effect in future studies either on clinical images as previously performed by Zwanenburg et al. [53], or relying on dedicated phantoms under development in our group. Another limitation of our study is the use of two different iodinated-contrast media (Ultravist® 370 and Visipaque® 320). While all the patients scanned on the Discovery CT750 HD scanner received the Ultravist® 370, in the two populations scanned on the Optima 660 scanner this type of contrast was injected only in about half of the patients (the 52% and the 56% at 100 kVp and 120 kVp, respectively). The administration of two different contrast media may have affected the texture of the CT image. However, a previous study of our group performed on CT images of NSCLC patients [14] showed that the radiomic features were not significantly influenced by the different contrast media, and therefore this factor was not investigated in this study.

In conclusion, the present study confirmed that the use of different blending levels during CT reconstruction may introduce confounding factors in a radiomic analysis of NSCLC population, especially when a wide range of different blending levels are present in the dataset. Aiming to improve the robustness and efficacy of radiomic studies, a novel approach for the identification of reproducible features in a given dataset is proposed, to be applied before redundancy reduction and correlation analysis with clinical endpoints.

## Supplementary Information

**Additional file 1: Supplementary Methods.** Details on the extracted radiomic features. **Table S1**. List of radiomic features included in the study: the name of the feature and the corresponding category, sub-category and cluster is reported. **Table S2.** False Discovery Rate (FDR) adjusted p values for univariate and multivariable analysis for the effect of scanner and tube voltage on selected radiomic fetaures^. **Table S3.** OCCC values and False Discovery Rate (FDR) adjusted p values from the multivariable mixed model for the reconstruction algorithm impact for all the original features (shape features are not included because their value was always 1 since the same VOI was used for all the reconstructions). **Table S4.** OCCC values and False Discovery Rate (FDR) adjusted p value from the multivariable mixed model for the reconstruction algorithm impact for all the wavelet features. **Table S5.** OCCC values and False Discovery Rate (FDR) adjusted p value from the multivariable mixed model for the reconstruction algorithm impact (FDR corrected) for all the LoG features. **Table S6.** Percentage of features falling in each of the 4 groups, for the IR50 and the IR80 reconstructions. The results reported in this table refer to the sub-analysis performed on IR40, IR50, IR60 and IR80 (taking IR40 for comparison), similarly to Table 4 for the complete analysis. The percentage for the original images is evaluated excluding the features of the shape category. **Figure S1.** Study flowchart with exclusion criteria. **Figure S2.** Overall Concordance Correlation Coefficient (OCCC) for concordance between different algorithms for the sub-analysis of the blending levels mostly used in our clinical setting (IR40, IR50, IR60 and IR80). The OCCC is plotted within each subtype of feature and for feature extracted from the original images (a), and the Wavelet (b) and LoG-filtered (c) images. **Figure S3.** Heatmap representing the number of features significantly different for paired comparisons of reconstruction algorithm strength (Wilcoxon signed rank-test), for the original images. Shape feature excluded. **Figure S4.** Box plot representing the distribution of the radiomic features (a) *original_glrlm_RunVariance* and (b) modified *original_glrlm_RunVariance,* according to algorithm. The modified *original_glrlm_RunVariance* was obtained after rescaling, using the correspondent coefficients for each algorithm obtained in the mixed model. Minimum and maximum are depicted by whiskers, the box signifies the upper and lower quartiles, the median and the mean are represented, respectively by a line and a small rhombus within the box.

## Data Availability

The datasets used and/or analysed during the current study are available from the corresponding author on reasonable request.

## Abbreviations

ASIR: Adaptive statistical iterative reconstruction
CT: Computed tomography
FBP: Filtered backprojection
FDR: False discovery rate
GLCM: Grey level co-occurrence matrix
GLDM: Grey level dependence matrix
GLRLM: Grey level run length matrix
GLSZM: Grey level size zone matrix
HU: Hounsfield unit
IR: Iterative reconstruction
LoG: Laplacian of Gaussian
NGTDM: Neighbouring grey tone difference matrix
NSCLC: Non-small-cell lung cancer
OCCC: Overall concordance correlation coefficient
