# Supplementary material for: Reproducibility of radiomic features in CT images of NSCLC patients: an integrative analysis on the impact of acquisition and reconstruction parameters
Source: Eur Radiol Exp. 2022 Jan 25;6:2. doi: 10.1186/s41747-021-00258-6 (PMC8786992; doi:10.1186/s41747-021-00258-6)
Supplement: Supplementary file 1 — Additional file 1: Supplementary Methods. Details on the extracted radiomic features. Table S1. List of radiomic features included in the study: the name of the feature and the corresponding category, sub-category and cluster is reported. Table S2. False Discovery Rate (FDR) adjusted p values for univariate and multivariable analysis for the effect of scanner and tube voltage on selected radiomic fetaures^. Table S3. OCCC values and False Discovery Rate (FDR) adjusted p values from the multivariable mixed model for the reconstruction algorithm impact for all the original features (shape features are not included because their value was always 1 since the same VOI was used for all the reconstructions). Table S4. OCCC values and False Discovery Rate (FDR) adjusted p value from the multivariable mixed model for the reconstruction algorithm impact for all the wavelet features. Table S5. OCCC values and False Discovery Rate (FDR) adjusted p value from the multivariable mixed model for the reconstruction algorithm impact (FDR corrected) for all the LoG features. Table S6. Percentage of features falling in each of the 4 groups, for the IR50 and the IR80 reconstructions. The results reported in this table refer to the sub-analysis performed on IR40, IR50, IR60 and IR80 (taking IR40 for comparison), similarly to Table 4 for the complete analysis. The percentage for the original images is evaluated excluding the features of the shape category. Figure S1. Study flowchart with exclusion criteria. Figure S2. Overall Concordance Correlation Coefficient (OCCC) for concordance between different algorithms for the sub-analysis of the blending levels mostly used in our clinical setting (IR40, IR50, IR60 and IR80). The OCCC is plotted within each subtype of feature and for feature extracted from the original images (a), and the Wavelet (b) and LoG-filtered (c) images. Figure S3. Heatmap representing the number of features significantly different for paired comparisons of reconstructio [file 41747_2021_258_MOESM1_ESM.docx]

**ELECTRONIC SUPPLEMENTARY MATERIAL**

**Supplementary Methods**

*Details on the extracted radiomic features*

The CT images and the corresponding RT Structure files were converted from DICOM (Digital Imaging and COmmunications in Medicine) to NRRD (Nearly Raw Raster Data) format with the open-source software Slicer (v. 4.10.0) and uploaded in Python (v. 3.7) environment.

Radiomic features were extracted with Pyradiomics v. 2.2.0. All the features implemented in Pyradiomics were calculated: *Shape* features which describe the VOI from a geometric point of view, analysing the properties of the volume itself and of its surface; the *first order* features quantifying the properties of the voxel intensity histogram inside the VOI; the remaining features, referred to as second order features, describe the distribution of voxel intensities inside the VOI by taking into account the spatial relationships between a pixel and its neighbours (*glcm*, *glszm*, *glrlm*, *gldm*, *ngtdm*). The mathematical definition of each feature and its compatibility with the IBSI can be found in the online Pyradiomics Documentation (<https://pyradiomics.readthedocs.io/_/downloads/en/2.2.0/pdf/>).

Both *glcm* and *ngtdm* matrices were calculated considering three different offsets (1, 4 and 7) between the reference and the neighbour voxel.

The 2D setting was applied for the calculation of all features, meaning that every feature was calculated on each axial slice and then the average value among all the VOI slices was returned as an output, as recommended in presence of not-isotropic voxels (<https://arxiv.org/pdf/1612.07003.pdf>).

Features that were not included in the analysis are : Energy feature from *First Order* category and JointAverage features from the *glcm* category, because they provide identical output to the TotalEnergy and SumAverage features, respectively; Busyness from the *ngtdm* category, GrayLevelNonUniformity and RunLengthNonUniformity from the *glrlm* category, GrayLevelNonUniformity from the *glszm* category and DependenceNonUniformity from the *gldm* category because their formula intrinsically include a dependence on the number of voxels in the VOI (N) [1,2]. For these last four features, the normalised version already implemented in Pyradiomics was taken into consideration, since a dependence on the number of voxels was not observed for this version. Additionally, the TotalEnergy feature from the *First Order* category, the Coarseness feature from the *ngtdm* category and the GrayLevelNonUniformity feature from the *gldm* category were corrected *a posteriori* for their intrinsic dependence on the number of voxels in the VOI, respectively by dividing [1], multiplying [2], and again dividing by N. Since no reference was found in the literature for the *gldm* category*,* GrayLevelNonUniformity was adjusted following the same method of the corresponding feature in the *glrlm* category [1], observing a reduction in the correlation with the number of voxels.

The features were extracted from non-filtered and filtered images. The filters available in Pyradiomics and applied in this study were the Wavelet and LoG ones. The Wavelet transform, for 2D extraction, can capture the details in horizontal (Low-High pass filter, LH), vertical (High-Low pass filter, HL) and diagonal (High-High pass filter, HH) or can create a smoothed approximation of the original image (Low-Low pass filter, LL), by filtering the images twice sequentially. The LoG filter, instead, is the application in sequence of a Gaussian and a Laplacian filter. The second derivative on a smoothed image, because it is the combination of a Gaussian filter, followed by a Laplacian one. The Gaussian filter is used to reduce the noise inside the image, while the Laplacian filter highlights gray-level discontinuities, enhancing the details of the texture. The different smoothing effect of the Gaussian filter was taken in consideration by choosing the following values for the standard deviation (*Sigma*).

A summary of all parameters set in Pyradiomics during the feature extraction is given below. The remaining customisable parameters were set to default values.

| **imageType:** | Original: {}  Wavelet:  wavelet: coif1  LoG:  sigma: [0.5, 1.0, 1.5, 2.5, 5.0] |
| --- | --- |
| **featureClass:** | shape  firstorder  glrlm  glszm  gldm  glcm  ngtdm |
| **resampledPixelSpacing:** | [0.78, 0.78, 0] * |
| **interpolator:** | 'sitkBSpline' |
| **binWidth:** | 25 |
| **distances:** | [1], [4], [7] |
| **force2D:** | true |
| **force2Ddimension:** | 0 |
| **voxelArrayShift:** | 1000 |

***** pixel size was resampled considering the average value among the 103 images included in the study (range 0.52 – 1.38 mm).

1. Fave, X.; Zhang, L.; Yang, J.; Mackin, D.; Balter, P.; Gomez, D.; Followill, D.; Jones, A.K.; Stingo, F.; Court, L. Impact of Image Preprocessing on the Volume Dependence and Prognostic Potential of Radiomics Features in Non-Small Cell Lung Cancer. *Transl. Cancer Res.* **2016**, *5*, doi:10.21037/8709.

2. Shafiq-Ul-Hassan, M.; Latifi, K.; Zhang, G.; Ullah, G.; Gillies, R.; Moros, E. Voxel Size and Gray Level Normalization of CT Radiomic Features in Lung Cancer. *Sci. Rep.* **2018**, *8*, 10545, doi:10.1038/s41598-018-28895-9.

**Table S1. List of radiomic features included in the study: the name of the feature and the corresponding category, sub-category and cluster is reported.**

| **Features** | **Category** | **Sub-category** | **Cluster*** |
| --- | --- | --- | --- |
| Elongation | shape |  | 1 |
| Flatness | shape |  | 2 |
| MeshVolume | shape |  | 3 |
| VoxelVolume | shape |  | 3 |
| SurfaceArea | shape |  | 3 |
| MinorAxisLength | shape |  | 3 |
| LeastAxisLength | shape |  | 3 |
| Maximum2DDiameterColumn | shape |  | 3 |
| Maximum3DDiameter | shape |  | 4 |
| Maximum2DDiameterRow | shape |  | 4 |
| MajorAxisLength | shape |  | 4 |
| Maximum2DDiameterSlice | shape |  | 4 |
| Sphericity | shape |  | 5 |
| SurfaceVolumeRatio | shape |  | 6 |
| SumSquares | texture | glcm1 | 7 |
| Variance | firstorder |  | 7 |
| GrayLevelVariance | texture | gldm | 7 |
| ClusterTendency | texture | glcm1 | 7 |
| SumSquares | texture | glcm4 | 7 |
| SumSquares | texture | glcm7 | 7 |
| MeanAbsoluteDeviation | firstorder |  | 7 |
| ClusterTendency | texture | glcm4 | 7 |
| ClusterTendency | texture | glcm7 | 7 |
| GrayLevelVariance | texture | glrlm | 7 |
| 10Percentile | firstorder |  | 7 |
| GrayLevelNonUniformityNormalized | texture | glszm | 7 |
| RunEntropy | texture | glrlm | 7 |
| 90Percentile | firstorder |  | 8 |
| Median | firstorder |  | 8 |
| SumEntropy | texture | glcm1 | 9 |
| Entropy | firstorder |  | 9 |
| SumEntropy | texture | glcm4 | 9 |
| JointEntropy | texture | glcm4 | 9 |
| SumEntropy | texture | glcm7 | 9 |
| JointEntropy | texture | glcm7 | 9 |
| JointEntropy | texture | glcm1 | 9 |
| DependenceEntropy | texture | gldm | 9 |
| GrayLevelNonUniformityNormalized | texture | glrlm | 9 |
| GrayLevelNonUniformity_modified | texture | gldm | 9 |
| Uniformity | firstorder |  | 9 |
| InterquartileRange | firstorder |  | 9 |
| RobustMeanAbsoluteDeviation | firstorder |  | 9 |
| Mean | firstorder |  | 10 |
| Kurtosis | firstorder |  | 10 |
| RootMeanSquared | firstorder |  | 10 |
| Skewness | firstorder |  | 10 |
| TotalEnergy_modified | firstorder |  | 10 |
| Maximum | firstorder |  | 11 |
| Minimum | firstorder |  | 12 |
| Range | firstorder |  | 12 |
| Autocorrelation | texture | glcm1 | 13 |
| SumAverage | texture | glcm4 | 13 |
| HighGrayLevelEmphasis | texture | gldm | 13 |
| SumAverage | texture | glcm1 | 13 |
| SumAverage | texture | glcm7 | 13 |
| Autocorrelation | texture | glcm4 | 13 |
| Autocorrelation | texture | glcm7 | 13 |
| HighGrayLevelRunEmphasis | texture | glrlm | 13 |
| HighGrayLevelZoneEmphasis | texture | glszm | 13 |
| SmallAreaHighGrayLevelEmphasis | texture | glszm | 13 |
| ShortRunHighGrayLevelEmphasis | texture | glrlm | 13 |
| LongRunEmphasis | texture | glrlm | 14 |
| LargeDependenceEmphasis | texture | gldm | 14 |
| RunPercentage | texture | glrlm | 14 |
| RunVariance | texture | glrlm | 14 |
| DependenceNonUniformityNormalized | texture | gldm | 14 |
| LongRunHighGrayLevelEmphasis | texture | glrlm | 15 |
| LargeDependenceHighGrayLevelEmphasis | texture | gldm | 15 |
| LongRunLowGrayLevelEmphasis | texture | glrlm | 16 |
| LargeDependenceLowGrayLevelEmphasis | texture | gldm | 16 |
| ShortRunLowGrayLevelEmphasis | texture | glrlm | 17 |
| LowGrayLevelRunEmphasis | texture | glrlm | 17 |
| LowGrayLevelZoneEmphasis | texture | glszm | 17 |
| LowGrayLevelEmphasis | texture | gldm | 17 |
| SmallAreaLowGrayLevelEmphasis | texture | glszm | 17 |
| SmallDependenceLowGrayLevelEmphasis | texture | gldm | 17 |
| Id | texture | glcm4 | 18 |
| Idm | texture | glcm4 | 18 |
| Id | texture | glcm1 | 18 |
| Idm | texture | glcm1 | 18 |
| Id | texture | glcm7 | 18 |
| Idm | texture | glcm7 | 18 |
| RunLengthNonUniformityNormalized | texture | glrlm | 18 |
| ShortRunEmphasis | texture | glrlm | 18 |
| LargeAreaEmphasis | texture | glszm | 18 |
| ZoneVariance | texture | glszm | 18 |
| LargeAreaLowGrayLevelEmphasis | texture | glszm | 18 |
| LargeAreaHighGrayLevelEmphasis | texture | glszm | 18 |
| ClusterProminence | texture | glcm1 | 19 |
| ClusterShade | texture | glcm4 | 19 |
| ClusterShade | texture | glcm7 | 19 |
| ClusterProminence | texture | glcm4 | 19 |
| ClusterProminence | texture | glcm7 | 19 |
| ClusterShade | texture | glcm1 | 19 |
| GrayLevelVariance | texture | glszm | 19 |
| Strength | texture | ngtdm4 | 20 |
| Strength | texture | ngtdm7 | 20 |
| Strength | texture | ngtdm1 | 20 |
| SizeZoneNonUniformity | texture | glszm | 20 |
| SmallAreaEmphasis | texture | glszm | 21 |
| SizeZoneNonUniformityNormalized | texture | glszm | 21 |
| InverseVariance | texture | glcm1 | 21 |
| ZoneEntropy | texture | glszm | 22 |
| DifferenceEntropy | texture | glcm1 | 23 |
| DifferenceEntropy | texture | glcm4 | 23 |
| Idn | texture | glcm7 | 23 |
| DifferenceAverage | texture | glcm4 | 23 |
| DifferenceEntropy | texture | glcm7 | 23 |
| Idn | texture | glcm4 | 23 |
| DifferenceAverage | texture | glcm1 | 23 |
| SmallDependenceEmphasis | texture | gldm | 23 |
| Idmn | texture | glcm7 | 23 |
| Idmn | texture | glcm4 | 23 |
| Contrast | texture | ngtdm1 | 23 |
| ZonePercentage | texture | glszm | 23 |
| DifferenceAverage | texture | glcm7 | 23 |
| Contrast | texture | ngtdm4 | 23 |
| InverseVariance | texture | glcm7 | 23 |
| Contrast | texture | ngtdm7 | 23 |
| InverseVariance | texture | glcm4 | 23 |
| Idn | texture | glcm1 | 23 |
| Idmn | texture | glcm1 | 23 |
| DependenceVariance | texture | gldm | 24 |
| Complexity | texture | ngtdm4 | 25 |
| Complexity | texture | ngtdm7 | 25 |
| Complexity | texture | ngtdm1 | 25 |
| SmallDependenceHighGrayLevelEmphasis | texture | gldm | 25 |
| Contrast | texture | glcm4 | 26 |
| DifferenceVariance | texture | glcm4 | 26 |
| Contrast | texture | glcm7 | 26 |
| DifferenceVariance | texture | glcm7 | 26 |
| Contrast | texture | glcm1 | 26 |
| DifferenceVariance | texture | glcm1 | 26 |
| Correlation | texture | glcm4 | 27 |
| MCC | texture | glcm1 | 27 |
| Correlation | texture | glcm1 | 27 |
| Correlation | texture | glcm7 | 27 |
| Imc1 | texture | glcm1 | 28 |
| Imc2 | texture | glcm1 | 29 |
| Coarseness_modified | texture | ngtdm1 | 29 |
| JointEnergy | texture | glcm4 | 30 |
| JointEnergy | texture | glcm7 | 30 |
| MaximumProbability | texture | glcm7 | 30 |
| MaximumProbability | texture | glcm4 | 30 |
| MaximumProbability | texture | glcm1 | 30 |
| JointEnergy | texture | glcm1 | 30 |
| MCC | texture | glcm7 | 31 |
| MCC | texture | glcm4 | 31 |
| Imc1 | texture | glcm4 | 31 |
| Imc2 | texture | glcm7 | 32 |
| Imc2 | texture | glcm4 | 32 |
| Imc1 | texture | glcm7 | 32 |
| Coarseness_modified  Coarseness_modified | texture  texture | ngtdm4  ngtdm7 | 33  33 |

*cluster analysis was performed on the original, not-filtered, radiomic features

**Table S2. False Discovery Rate (FDR) adjusted p values for univariate and multivariable analysis for the effect of scanner and tube voltage on selected radiomic fetaures^.**

| **Features** | **Filter** | **Scanner (univar)**  **FBP** | **Scanner (univar)**  **IR60** | **Tube voltage (univar) FBP** | **Tube voltage (univar) IR60** | **Scanner (multivar)** | **Tube voltage (multivar)** |
| --- | --- | --- | --- | --- | --- | --- | --- |
| shape_SurfaceArea | original | 0.90 | 0.96 | 0.74 | 0.70 | **0.03** | 0.89 |
| shape_VoxelVolume | original | 0.94 | 0.96 | 0.78 | 0.70 | 0.19° | **<0.0001°** |
| firstorder_10Percentile | wavelet-HH | 0.24 | 0.90 | **0.01** | 0.51 | 1.00 | 0.81 |
| firstorder_90Percentile | wavelet-HH | 0.26 | 0.90 | **0.01** | 0.53 | 1.00 | 0.81 |
| firstorder_Entropy | wavelet-HH | 0.47 | 0.92 | **0.05** | 0.53 | 1.00 | 0.75 |
| firstorder_Interquartile Range | wavelet-HH | 0.23 | 0.90 | **0.01** | 0.37 | 1.00 | 0.75 |
| firstorder_MeanAbsolute Deviation | wavelet-HH | 0.26 | 0.90 | **0.01** | 0.40 | 1.00 | 0.66 |
| firstorder_RobustMean AbsoluteDeviation | wavelet-HH | 0.23 | 0.90 | **0.01** | 0.40 | 1.00 | 0.75 |
| firstorder_Uniformity | wavelet-HH | 0.47 | 0.94 | **0.05** | 0.53 | 1.00 | 0.81 |
| firstorder_Variance | wavelet-HH | 0.39 | 0.90 | **0.01** | 0.42 | 1.00 | 0.67 |
| glcm1_ClusterShade | wavelet-HL | 0.62 | 0.90 | **0.05** | 0.14 | 1.00 | 0.25 |
| glcm1_Contrast | wavelet-HH | 0.42 | 0.90 | **0.01** | 0.22 | 1.00 | 0.60 |
| glcm1_Correlation | wavelet-HH | 0.46 | 0.91 | 0.14 | 0.13 | 0.56 | **0.02** |
| glcm1_Correlation | wavelet-HL | 0.95 | 1.00 | 0.07 | **0.04** | 1.00 | 0.18 |
| glcm1_DifferenceAverage | wavelet-HH | 0.26 | 0.90 | **0.003** | 0.13 | 1.00 | 0.44 |
| glcm1_DifferenceEntropy | wavelet-HH | 0.46 | 0.90 | **0.04** | 0.47 | 1.00 | 0.67 |
| glcm1_DifferenceVariance | wavelet-HH | 0.46 | 0.90 | **0.05** | 0.35 | 1.00 | 0.66 |
| glcm1_Id | wavelet-HH | 0.23 | 0.90 | **0.002** | 0.06 | 1.00 | 0.34 |
| glcm1_Idm | wavelet-HH | 0.24 | 0.90 | **0.002** | 0.09 | 1.00 | 0.35 |
| glcm1_InverseVariance | wavelet-HH | 0.23 | 0.91 | **0.004** | 0.10 | 0.31 | **0.01** |
| glcm4_ClusterShade | wavelet-HL | 0.46 | 0.90 | **0.04** | 0.16 | 1.00 | 0.19 |
| gldm_GrayLevelNonUniformity_modified | wavelet-HH | 0.47 | 0.94 | **0.05** | 0.53 | 1.00 | 0.81 |
| gldm_GrayLevelVariance | wavelet-HH | 0.48 | 0.92 | **0.05** | 0.42 | 1.00 | 0.72 |
| gldm_LargeDependence Emphasis | wavelet-HH | 0.23 | 0.90 | **0.002** | 0.04 | 1.00 | 0.12 |
| gldm_LargeDependence LowGrayLevelEmphasis | wavelet-HH | 0.75 | 0.92 | **0.05** | 0.23 | 1.00 | 0.40 |
| gldm_SmallDependence Emphasis | wavelet-HH | 0.41 | 0.90 | **0.01** | 0.34 | 1.00 | 0.60 |
| gldm_SmallDependence HighGrayLevelEmphasis | wavelet-HH | 0.73 | 0.94 | **0.05** | 0.36 | 1.00 | 0.35 |
| glrlm_GrayLevelNonUniformityNormalized | wavelet-HH | 0.47 | 0.94 | **0.05** | 0.53 | 1.00 | 0.79 |
| glrlm_GrayLevelVariance | wavelet-HH | 0.51 | 0.92 | **0.05** | 0.40 | 1.00 | 0.67 |
| glrlm_LongRunEmphasis | wavelet-HH | 0.23 | 0.90 | **0.003** | 0.16 | 1.00 | 0.33 |
| glrlm_RunPercentage | wavelet-HH | 0.23 | 0.90 | **0.002** | 0.07 | 1.00 | 0.27 |
| glrlm_RunVariance | wavelet-HH | 0.23 | 0.90 | **0.01** | 0.29 | 1.00 | 0.35 |
| glrlm_ShortRunEmphasis | wavelet-HH | 0.28 | 0.90 | **0.003** | 0.16 | 1.00 | 0.35 |
| glszm_GrayLevelVariance | wavelet-HH | 0.46 | 0.90 | **0.01** | **0.05** | 1.00 | 0.08 |
| glszm_SizeZoneNonUniformityNormalized | wavelet-HH | 0.26 | 0.91 | **0.01** | **0.02** | 1.00 | **0.01** |
| glszm_SizeZoneNonUniformityNormalized | wavelet-HL | 0.88 | 0.90 | **0.05** | 0.14 | 1.00 | 0.34 |
| glszm_SmallAreaEmphasis | wavelet-HH | 0.26 | 0.91 | **0.01** | **0.02** | 1.00 | **0.01** |
| glszm_SmallAreaEmphasis | wavelet-HL | 0.88 | 0.90 | **0.05** | 0.14 | 1.00 | 0.35 |
| glszm_SmallAreaHighGrayLevelEmphasis | wavelet-HH | 0.84 | 0.94 | **0.05** | 0.16 | 1.00 | 0.21 |
| glszm_ZoneEntropy | wavelet-HH | 0.29 | 0.90 | **0.02** | 0.51 | 1.00 | 0.33 |
| glszm_ZoneEntropy | wavelet-HL | 0.64 | 0.97 | **0.05** | 0.28 | 1.00 | 0.56 |
| glszm_ZonePercentage | wavelet-HH | 0.47 | 0.94 | **0.05** | 0.51 | 1.00 | 0.81 |
| ngtdm4_Coarseness_ modified | wavelet-HH | 0.46 | 0.94 | **0.04** | 0.53 | 1.00 | 0.79 |
| ngtdm7_Coarseness_ modified | wavelet-HH | 0.46 | 0.92 | **0.05** | 0.53 | 1.00 | 0.81 |

^: features with significant FDR-adjusted p values at either univariate or multivariable analysis or both

°: in the model with VoxelVolume as the dependent variable, clinical volume was not used as independent predictor

**Table S3. OCCC values and False Discovery Rate (FDR) adjusted p values from the multivariable mixed model for the reconstruction algorithm impact for all the original features** (shape features are not included because their value was always 1 since the same VOI was used for all the reconstructions).

| **Feature** | **Category** | **Sub-category** | **OCCC** | **IR20**  **(mixed)** | **IR40 (mixed)** | **IR50 (mixed)** | **IR60 (mixed)** | **IR80 (mixed)** |
| --- | --- | --- | --- | --- | --- | --- | --- | --- |
| 10Percentile | firstorder |  | 1.00 | 0.17 | **0.003** | **0.0003** | **<0.0001** | **<0.0001** |
| 90Percentile | firstorder |  | 1.00 | **<0.0001** | **<0.0001** | **<0.0001** | **<0.0001** | **<0.0001** |
| Entropy | firstorder |  | 0.99 | **<0.0001** | **<0.0001** | **<0.0001** | **<0.0001** | **<0.0001** |
| InterquartileRange | firstorder |  | 1.00 | **<0.0001** | **<0.0001** | **<0.0001** | **<0.0001** | **<0.0001** |
| Kurtosis | firstorder |  | 0.96 | 0.35 | **0.04** | **0.008** | **0.001** | **<0.0001** |
| Maximum | firstorder |  | 0.99 | 0.60 | 0.60 | 0.84 | 0.77 | **0.007** |
| Mean | firstorder |  | 1.00 | **<0.0001** | **<0.0001** | **<0.0001** | **<0.0001** | **<0.0001** |
| MeanAbsolute  Deviation | firstorder |  | 1.00 | 0.42 | 0.18 | 0.15 | 0.15 | 0.28 |
| Median | firstorder |  | 1.00 | **<0.0001** | **<0.0001** | **<0.0001** | **<0.0001** | **<0.0001** |
| Minimum | firstorder |  | 0.97 | **<0.0001** | **<0.0001** | **<0.0001** | **<0.0001** | **<0.0001** |
| Range | firstorder |  | 0.99 | **0.01** | **<0.0001** | **<0.0001** | **<0.0001** | **<0.0001** |
| RobustMeanAbsoluteDeviation | firstorder |  | 1.00 | **<0.0001** | **<0.0001** | **<0.0001** | **<0.0001** | **<0.0001** |
| RootMeanSquared | firstorder |  | 1.00 | **<0.0001** | **<0.0001** | **<0.0001** | **<0.0001** | **<0.0001** |
| Skewness | firstorder |  | 0.99 | **0.006** | **<0.0001** | **<0.0001** | **<0.0001** | **<0.0001** |
| TotalEnergy_modified | firstorder |  | 1.00 | **<0.0001** | **<0.0001** | **<0.0001** | **<0.0001** | **<0.0001** |
| Uniformity | firstorder |  | 0.98 | **<0.0001** | **<0.0001** | **<0.0001** | **<0.0001** | **<0.0001** |
| Variance | firstorder |  | 1.00 | **0.002** | **<0.0001** | **<0.0001** | **<0.0001** | **<0.0001** |
| Autocorrelation | texture | glcm1 | 0.97 | **0.002** | **<0.0001** | **<0.0001** | **<0.0001** | **<0.0001** |
| ClusterProminence | texture | glcm1 | 1.00 | **<0.0001** | **<0.0001** | **<0.0001** | **<0.0001** | **<0.0001** |
| ClusterShade | texture | glcm1 | 1.00 | **<0.0001** | **<0.0001** | **<0.0001** | **<0.0001** | **<0.0001** |
| ClusterTendency | texture | glcm1 | 1.00 | **0.01** | **<0.0001** | **<0.0001** | **<0.0001** | **<0.0001** |
| Contrast | texture | glcm1 | 0.99 | **0.002** | **<0.0001** | **<0.0001** | **<0.0001** | **<0.0001** |
| Correlation | texture | glcm1 | 0.98 | 0.46 | **0.02** | **0.001** | **<0.0001** | **<0.0001** |
| DifferenceAverage | texture | glcm1 | 0.99 | **<0.0001** | **<0.0001** | **<0.0001** | **<0.0001** | **<0.0001** |
| DifferenceEntropy | texture | glcm1 | 0.98 | **<0.0001** | **<0.0001** | **<0.0001** | **<0.0001** | **<0.0001** |
| DifferenceVariance | texture | glcm1 | 0.97 | **<0.0001** | **<0.0001** | **<0.0001** | **<0.0001** | **<0.0001** |
| Id | texture | glcm1 | 0.88 | **<0.0001** | **<0.0001** | **<0.0001** | **<0.0001** | **<0.0001** |
| Idm | texture | glcm1 | 0.89 | **<0.0001** | **<0.0001** | **<0.0001** | **<0.0001** | **<0.0001** |
| Idmn | texture | glcm1 | 1.00 | 0.13 | **<0.0001** | **<0.0001** | **<0.0001** | **<0.0001** |
| Idn | texture | glcm1 | 0.98 | **<0.0001** | **<0.0001** | **<0.0001** | **<0.0001** | **<0.0001** |
| Imc1 | texture | glcm1 | **0.65*** | **<0.0001** | **<0.0001** | **<0.0001** | **<0.0001** | **<0.0001** |
| Imc2 | texture | glcm1 | 0.87 | **<0.0001** | **<0.0001** | **<0.0001** | **<0.0001** | **<0.0001** |
| InverseVariance | texture | glcm1 | **0.68*** | **<0.0001** | **<0.0001** | **<0.0001** | **<0.0001** | **<0.0001** |
| JointEnergy | texture | glcm1 | 0.86 | **<0.0001** | **<0.0001** | **<0.0001** | **<0.0001** | **<0.0001** |
| JointEntropy | texture | glcm1 | 0.98 | **<0.0001** | **<0.0001** | **<0.0001** | **<0.0001** | **<0.0001** |
| MaximumProbability | texture | glcm1 | **0.82*** | **<0.0001** | **<0.0001** | **<0.0001** | **<0.0001** | **<0.0001** |
| MCC | texture | glcm1 | 0.98 | 0.92 | 0.55 | 0.60 | 0.74 | 1.00 |
| SumAverage | texture | glcm1 | 0.97 | **0.0008** | **<0.0001** | **<0.0001** | **<0.0001** | **<0.0001** |
| SumEntropy | texture | glcm1 | 0.99 | **<0.0001** | **<0.0001** | **<0.0001** | **<0.0001** | **<0.0001** |
| SumSquares | texture | glcm1 | 1.00 | **0.008** | **<0.0001** | **<0.0001** | **<0.0001** | **<0.0001** |
| Autocorrelation | texture | glcm4 | 0.97 | **0.002** | **<0.0001** | **<0.0001** | **<0.0001** | **<0.0001** |
| ClusterProminence | texture | glcm4 | 1.00 | **0.0002** | **<0.0001** | **<0.0001** | **<0.0001** | **<0.0001** |
| ClusterShade | texture | glcm4 | 1.00 | **<0.0001** | **<0.0001** | **<0.0001** | **<0.0001** | **<0.0001** |
| ClusterTendency | texture | glcm4 | 1.00 | **0.02** | **<0.0001** | **<0.0001** | **<0.0001** | **<0.0001** |
| Contrast | texture | glcm4 | 1.00 | **0.005** | **<0.0001** | **<0.0001** | **<0.0001** | **<0.0001** |
| Correlation | texture | glcm4 | 1.00 | **0.05** | **<0.0001** | **<0.0001** | **<0.0001** | **<0.0001** |
| DifferenceAverage | texture | glcm4 | 1.00 | **<0.0001** | **<0.0001** | **<0.0001** | **<0.0001** | **<0.0001** |
| DifferenceEntropy | texture | glcm4 | 0.99 | **<0.0001** | **<0.0001** | **<0.0001** | **<0.0001** | **<0.0001** |
| DifferenceVariance | texture | glcm4 | 0.99 | **<0.0001** | **<0.0001** | **<0.0001** | **<0.0001** | **<0.0001** |
| Id | texture | glcm4 | 0.97 | **<0.0001** | **<0.0001** | **<0.0001** | **<0.0001** | **<0.0001** |
| Idm | texture | glcm4 | 0.97 | **<0.0001** | **<0.0001** | **<0.0001** | **<0.0001** | **<0.0001** |
| Idmn | texture | glcm4 | 1.00 | 0.52 | 0.30 | **0.005** | **0.0001** | **<0.0001** |
| Idn | texture | glcm4 | 1.00 | **<0.0001** | **<0.0001** | **<0.0001** | **<0.0001** | **<0.0001** |
| Imc1 | texture | glcm4 | 0.92 | **<0.0001** | **<0.0001** | **<0.0001** | **<0.0001** | **<0.0001** |
| Imc2 | texture | glcm4 | 0.96 | **<0.0001** | **<0.0001** | **<0.0001** | **<0.0001** | **<0.0001** |
| InverseVariance | texture | glcm4 | 0.99 | **0.01** | **0.002** | **0.02** | 0.22 | **0.02** |
| JointEnergy | texture | glcm4 | 0.93 | **<0.0001** | **<0.0001** | **<0.0001** | **<0.0001** | **<0.0001** |
| JointEntropy | texture | glcm4 | 0.99 | **<0.0001** | **<0.0001** | **<0.0001** | **<0.0001** | **<0.0001** |
| MaximumProbability | texture | glcm4 | 0.90 | **<0.0001** | **<0.0001** | **<0.0001** | **<0.0001** | **<0.0001** |
| MCC | texture | glcm4 | 0.98 | **0.0002** | **<0.0001** | **<0.0001** | **<0.0001** | **<0.0001** |
| SumAverage | texture | glcm4 | 0.97 | **0.001** | **<0.0001** | **<0.0001** | **<0.0001** | **<0.0001** |
| SumEntropy | texture | glcm4 | 1.00 | **<0.0001** | **<0.0001** | **<0.0001** | **<0.0001** | **<0.0001** |
| SumSquares | texture | glcm4 | 1.00 | **0.01** | **<0.0001** | **<0.0001** | **<0.0001** | **<0.0001** |
| Autocorrelation | texture | glcm7 | 0.97 | **0.002** | **<0.0001** | **<0.0001** | **<0.0001** | **<0.0001** |
| ClusterProminence | texture | glcm7 | 1.00 | **0.0001** | **<0.0001** | **<0.0001** | **<0.0001** | **<0.0001** |
| ClusterShade | texture | glcm7 | 1.00 | **<0.0001** | **<0.0001** | **<0.0001** | **<0.0001** | **<0.0001** |
| ClusterTendency | texture | glcm7 | 1.00 | **0.01** | **<0.0001** | **<0.0001** | **<0.0001** | **<0.0001** |
| Contrast | texture | glcm7 | 1.00 | **0.009** | **<0.0001** | **<0.0001** | **<0.0001** | **<0.0001** |
| Correlation | texture | glcm7 | 1.00 | 0.17 | **0.001** | **<0.0001** | **<0.0001** | **<0.0001** |
| DifferenceAverage | texture | glcm7 | 1.00 | **<0.0001** | **<0.0001** | **<0.0001** | **<0.0001** | **<0.0001** |
| DifferenceEntropy | texture | glcm7 | 1.00 | **<0.0001** | **<0.0001** | **<0.0001** | **<0.0001** | **<0.0001** |
| DifferenceVariance | texture | glcm7 | 1.00 | **<0.0001** | **<0.0001** | **<0.0001** | **<0.0001** | **<0.0001** |
| Id | texture | glcm7 | 0.98 | **<0.0001** | **<0.0001** | **<0.0001** | **<0.0001** | **<0.0001** |
| Idm | texture | glcm7 | 0.98 | **<0.0001** | **<0.0001** | **<0.0001** | **<0.0001** | **<0.0001** |
| Idmn | texture | glcm7 | 1.00 | 0.26 | **0.03** | **<0.0001** | **<0.0001** | **<0.0001** |
| Idn | texture | glcm7 | 1.00 | **<0.0001** | **<0.0001** | **<0.0001** | **<0.0001** | **<0.0001** |
| Imc1 | texture | glcm7 | 0.97 | **<0.0001** | **<0.0001** | **<0.0001** | **<0.0001** | **<0.0001** |
| Imc2 | texture | glcm7 | 0.98 | **<0.0001** | **<0.0001** | **<0.0001** | **<0.0001** | **<0.0001** |
| InverseVariance | texture | glcm7 | 1.00 | **<0.0001** | **<0.0001** | **<0.0001** | **<0.0001** | **<0.0001** |
| JointEnergy | texture | glcm7 | 0.94 | **<0.0001** | **<0.0001** | **<0.0001** | **<0.0001** | **<0.0001** |
| JointEntropy | texture | glcm7 | 0.99 | **<0.0001** | **<0.0001** | **<0.0001** | **<0.0001** | **<0.0001** |
| MaximumProbability | texture | glcm7 | 0.92 | **<0.0001** | **<0.0001** | **<0.0001** | **<0.0001** | **<0.0001** |
| MCC | texture | glcm7 | 0.99 | **<0.0001** | **<0.0001** | **<0.0001** | **<0.0001** | **<0.0001** |
| SumAverage | texture | glcm7 | 0.97 | **0.001** | **<0.0001** | **<0.0001** | **<0.0001** | **<0.0001** |
| SumEntropy | texture | glcm7 | 1.00 | **<0.0001** | **<0.0001** | **<0.0001** | **<0.0001** | **<0.0001** |
| SumSquares | texture | glcm7 | 1.00 | **0.01** | **<0.0001** | **<0.0001** | **<0.0001** | **<0.0001** |
| DependenceEntropy | texture | gldm | 0.99 | **0.0002** | **<0.0001** | **<0.0001** | **<0.0001** | **<0.0001** |
| DependenceNon  UniformityNormalized | texture | gldm | 0.88 | **<0.0001** | **<0.0001** | **<0.0001** | **<0.0001** | **<0.0001** |
| DependenceVariance | texture | gldm | **0.56*** | **<0.0001** | **<0.0001** | **<0.0001** | **<0.0001** | **<0.0001** |
| GrayLevelNon  Uniformity_modified | texture | gldm | 1.00 | **<0.0001** | **<0.0001** | **<0.0001** | **<0.0001** | **<0.0001** |
| GrayLevelVariance | texture | gldm | 1.00 | **0.002** | **<0.0001** | **<0.0001** | **<0.0001** | **<0.0001** |
| HighGrayLevel  Emphasis | texture | gldm | 0.97 | **0.001** | **<0.0001** | **<0.0001** | **<0.0001** | **<0.0001** |
| LargeDependence  Emphasis | texture | gldm | **0.68*** | **<0.0001** | **<0.0001** | **<0.0001** | **<0.0001** | **<0.0001** |
| LargeDependenceHighGrayLevelEmphasis | texture | gldm | **0.71*** | **<0.0001** | **<0.0001** | **<0.0001** | **<0.0001** | **<0.0001** |
| LargeDependenceLowGrayLevelEmphasis | texture | gldm | 0.86 | **0.001** | **<0.0001** | **<0.0001** | **<0.0001** | **<0.0001** |
| LowGrayLevel  Emphasis | texture | gldm | 0.93 | 0.71 | 0.82 | 0.48 | **0.02** | **0.0001** |
| SmallDependence  Emphasis | texture | gldm | 0.96 | **<0.0001** | **<0.0001** | **<0.0001** | **<0.0001** | **<0.0001** |
| SmallDependenceHighGrayLevelEmphasis | texture | gldm | 0.92 | **<0.0001** | **<0.0001** | **<0.0001** | **<0.0001** | **<0.0001** |
| SmallDependenceLowGrayLevelEmphasis | texture | gldm | 0.96 | 0.86 | **0.05** | **0.004** | **0.0001** | **<0.0001** |
| GrayLevelNon  UniformityNormalized | texture | glrlm | 0.99 | 0.28 | 1.00 | 0.13 | **0.0002** | **<0.0001** |
| GrayLevelVariance | texture | glrlm | 0.99 | **<0.0001** | **<0.0001** | **<0.0001** | **<0.0001** | **<0.0001** |
| HighGrayLevelRun  Emphasis | texture | glrlm | 0.98 | 0.20 | **0.0007** | **<0.0001** | **<0.0001** | **<0.0001** |
| LongRunEmphasis | texture | glrlm | **0.59*** | **<0.0001** | **<0.0001** | **<0.0001** | **<0.0001** | **<0.0001** |
| LongRunHighGray  LevelEmphasis | texture | glrlm | **0.62*** | **<0.0001** | **<0.0001** | **<0.0001** | **<0.0001** | **<0.0001** |
| LongRunLowGray  LevelEmphasis | texture | glrlm | **0.78*** | **0.0011** | **<0.0001** | **<0.0001** | **<0.0001** | **<0.0001** |
| LowGrayLevelRun  Emphasis | texture | glrlm | 0.93 | 0.20 | 0.10 | 0.09 | 0.39 | 0.40 |
| RunEntropy | texture | glrlm | **0.82*** | **<0.0001** | **<0.0001** | **<0.0001** | **<0.0001** | **<0.0001** |
| RunLengthNonUniformityNormalized | texture | glrlm | 0.89 | **<0.0001** | **<0.0001** | **<0.0001** | **<0.0001** | **<0.0001** |
| RunPercentage | texture | glrlm | **0.77*** | **<0.0001** | **<0.0001** | **<0.0001** | **<0.0001** | **<0.0001** |
| RunVariance | texture | glrlm | **0.53*** | **<0.0001** | **<0.0001** | **<0.0001** | **<0.0001** | **<0.0001** |
| ShortRunEmphasis | texture | glrlm | 0.87 | **<0.0001** | **<0.0001** | **<0.0001** | **<0.0001** | **<0.0001** |
| ShortRunHighGray  LevelEmphasis | texture | glrlm | 0.95 | **<0.0001** | **<0.0001** | **<0.0001** | **<0.0001** | **<0.0001** |
| ShortRunLowGray  LevelEmphasis | texture | glrlm | 0.94 | 0.36 | 0.38 | 0.47 | 1.00 | 0.91 |
| GrayLevelNon  UniformityNormalized | texture | glszm | 0.97 | **<0.0001** | **<0.0001** | **<0.0001** | **<0.0001** | **<0.0001** |
| GrayLevelVariance | texture | glszm | 0.98 | **<0.0001** | **<0.0001** | **<0.0001** | **<0.0001** | **<0.0001** |
| HighGrayLevelZone  Emphasis | texture | glszm | 0.98 | 0.12 | **0.01** | **0.047** | 0.26 | 0.57 |
| LargeAreaEmphasis | texture | glszm | **0.79*** | 0.13 | **0.0003** | **<0.0001** | **<0.0001** | **<0.0001** |
| LargeAreaHighGray  LevelEmphasis | texture | glszm | **0.76*** | **0.04** | **<0.0001** | **<0.0001** | **<0.0001** | **<0.0001** |
| LargeAreaLowGray  LevelEmphasis | texture | glszm | **0.82*** | 0.27 | **0.01** | **0.0008** | **<0.0001** | **<0.0001** |
| LowGrayLevelZone  Emphasis | texture | glszm | 0.91 | **0.03** | **0.0005** | **0.0002** | **0.0009** | **0.0001** |
| SizeZoneNon  Uniformity | texture | glszm | 0.98 | **<0.0001** | **<0.0001** | **<0.0001** | **<0.0001** | **<0.0001** |
| SizeZoneNon  UniformityNormalized | texture | glszm | 0.98 | **0.0004** | **<0.0001** | **<0.0001** | **<0.0001** | **<0.0001** |
| SmallAreaEmphasis | texture | glszm | 0.98 | **0.002** | **<0.0001** | **<0.0001** | **<0.0001** | **<0.0001** |
| SmallAreaHighGrayLevelEmphasis | texture | glszm | 0.97 | 0.47 | 0.52 | 0.80 | 0.10 | **0.0006** |
| SmallAreaLowGray  LevelEmphasis | texture | glszm | 0.92 | **0.02** | **0.0003** | **0.0001** | **0.0001** | **<0.0001** |
| ZoneEntropy | texture | glszm | 0.92 | **0.0008** | **<0.0001** | **<0.0001** | **<0.0001** | **<0.0001** |
| ZonePercentage | texture | glszm | 0.96 | **<0.0001** | **<0.0001** | **<0.0001** | **<0.0001** | **<0.0001** |
| ZoneVariance | texture | glszm | **0.79*** | 0.13 | **0.0003** | **<0.0001** | **<0.0001** | **<0.0001** |
| Coarseness_modified | texture | ngtdm1 | 1.00 | 0.75 | 0.38 | 0.16 | **0.05** | **0.0004** |
| Complexity | texture | ngtdm1 | 0.97 | 0.11 | **0.006** | **0.02** | **0.006** | **0.01** |
| Contrast | texture | ngtdm1 | 1.00 | 0.48 | 0.52 | 0.12 | 0.16 | 0.16 |
| Strength | texture | ngtdm1 | 0.98 | **0.0001** | **<0.0001** | **<0.0001** | **<0.0001** | **<0.0001** |
| Coarseness_modified | texture | ngtdm4 | 1.00 | **<0.0001** | **<0.0001** | **<0.0001** | **<0.0001** | **<0.0001** |
| Complexity | texture | ngtdm4 | 0.99 | 0.47 | **0.03** | **0.0001** | **<0.0001** | **<0.0001** |
| Contrast | texture | ngtdm4 | 1.00 | 0.78 | 1.00 | 0.44 | 0.58 | 0.32 |
| Strength | texture | ngtdm4 | 0.98 | **0.001** | **<0.0001** | **<0.0001** | **<0.0001** | **<0.0001** |
| Coarseness_modified | texture | ngtdm7 | 1.00 | **<0.0001** | **<0.0001** | **<0.0001** | **<0.0001** | **<0.0001** |
| Complexity | texture | ngtdm7 | 0.99 | 0.27 | **0.003** | **<0.0001** | **<0.0001** | **<0.0001** |
| Contrast | texture | ngtdm7 | 1.00 | 0.73 | 0.96 | 0.33 | 0.40 | 0.15 |
| Strength | texture | ngtdm7 | 0.99 | **0.002** | **<0.0001** | **<0.0001** | **<0.0001** | **<0.0001** |

Notes: Significant FDR-adjusted p values for the multivariate model are in bold. OCCC < 0.85 are in bold and marked with an asterisk. Reference algorithm for mixed model analysis=FBP.

**Table S4. OCCC values and False Discovery Rate (FDR) adjusted p value from the multivariable mixed model for the reconstruction algorithm impact for all the wavelet features.**

| **Feature** | **Category** | **Sub-category** | **Filter** | **OCCC** | **IR20**  **(mixed)** | **IR40 (mixed)** | **IR50 (mixed)** | **IR60 (mixed)** | **IR80 (mixed)** |
| --- | --- | --- | --- | --- | --- | --- | --- | --- | --- |
| 10Percentile | firstorder |  | LH | 0.98 | 0.06 | **0.0004** | **<0.0001** | **<0.0001** | **<0.0001** |
|  |  |  | HL | 0.98 | 0.36 | 0.13 | 0.08 | 0.06 | 0.08 |
|  |  |  | HH | 0.88 | **0.002** | **<0.0001** | **<0.0001** | **<0.0001** | **<0.0001** |
|  |  |  | LL | 1.00 | 0.06 | **0.0002** | **<0.0001** | **<0.0001** | **<0.0001** |
| 90Percentile | firstorder |  | LH | 0.95 | **0.0005** | **<0.0001** | **<0.0001** | **<0.0001** | **<0.0001** |
|  |  |  | HL | 0.98 | **0.01** | **<0.0001** | **<0.0001** | **<0.0001** | **<0.0001** |
|  |  |  | HH | 0.88 | **0.008** | **<0.0001** | **<0.0001** | **<0.0001** | **<0.0001** |
|  |  |  | LL | 1.00 | **<0.0001** | **<0.0001** | **<0.0001** | **<0.0001** | **<0.0001** |
| Entropy | firstorder |  | LH | 0.97 | **<0.0001** | **<0.0001** | **<0.0001** | **<0.0001** | **<0.0001** |
|  |  |  | HL | 0.99 | **0.007** | **<0.0001** | **<0.0001** | **<0.0001** | **<0.0001** |
|  |  |  | HH | 0.91 | **<0.0001** | **<0.0001** | **<0.0001** | **<0.0001** | **<0.0001** |
|  |  |  | LL | 1.00 | **<0.0001** | **<0.0001** | **<0.0001** | **<0.0001** | **<0.0001** |
| InterquartileRange | firstorder |  | LH | 0.86 | **<0.0001** | **<0.0001** | **<0.0001** | **<0.0001** | **<0.0001** |
|  |  |  | HL | 0.91 | **<0.0001** | **<0.0001** | **<0.0001** | **<0.0001** | **<0.0001** |
|  |  |  | HH | **0.68*** | **<0.0001** | **<0.0001** | **<0.0001** | **<0.0001** | **<0.0001** |
|  |  |  | LL | 1.00 | **<0.0001** | **<0.0001** | **<0.0001** | **<0.0001** | **<0.0001** |
| Kurtosis | firstorder |  | LH | **0.77*** | **0.004** | **<0.0001** | **<0.0001** | **<0.0001** | **<0.0001** |
|  |  |  | HL | **0.80*** | **0.049** | **<0.0001** | **<0.0001** | **<0.0001** | **<0.0001** |
|  |  |  | HH | **0.43*** | **0.005** | **<0.0001** | **<0.0001** | **<0.0001** | **<0.0001** |
|  |  |  | LL | 0.98 | 0.30 | **0.03** | **0.006** | **0.0008** | **<0.0001** |
| Maximum | firstorder |  | LH | 0.93 | **<0.0001** | **<0.0001** | **<0.0001** | **<0.0001** | **<0.0001** |
|  |  |  | HL | 0.92 | **<0.0001** | **<0.0001** | **<0.0001** | **<0.0001** | **<0.0001** |
|  |  |  | HH | **0.84*** | **<0.0001** | **<0.0001** | **<0.0001** | **<0.0001** | **<0.0001** |
|  |  |  | LL | 1.00 | 0.81 | 0.66 | 0.24 | **0.04** | **<0.0001** |
| Mean | firstorder |  | LH | 1.00 | 0.72 | 0.46 | 0.35 | 0.26 | 0.14 |
|  |  |  | HL | 1.00 | 0.07 | **0.0002** | **<0.0001** | **<0.0001** | **<0.0001** |
|  |  |  | HH | 0.99 | 0.33 | 0.06 | **0.02** | **0.003** | **0.0001** |
|  |  |  | LL | 1.00 | **<0.0001** | **<0.0001** | **<0.0001** | **<0.0001** | **<0.0001** |
| MeanAbsolute  Deviation | firstorder |  | LH | 0.98 | **0.006** | **<0.0001** | **<0.0001** | **<0.0001** | **<0.0001** |
|  |  |  | HL | 0.99 | 0.18 | **0.01** | **0.005** | **0.003** | **0.004** |
|  |  |  | HH | 0.92 | **0.001** | **<0.0001** | **<0.0001** | **<0.0001** | **<0.0001** |
|  |  |  | LL | 1.00 | 0.58 | 0.36 | 0.32 | 0.32 | 0.41 |
| Median | firstorder |  | LH | 0.87 | **<0.0001** | **<0.0001** | **<0.0001** | **<0.0001** | **<0.0001** |
|  |  |  | HL | 0.90 | **<0.0001** | **<0.0001** | **<0.0001** | **<0.0001** | **<0.0001** |
|  |  |  | HH | **0.74*** | **<0.0001** | **<0.0001** | **<0.0001** | **<0.0001** | **<0.0001** |
|  |  |  | LL | 1.00 | **0.0001** | **<0.0001** | **<0.0001** | **<0.0001** | **<0.0001** |
| Minimum | firstorder |  | LH | 0.90 | **<0.0001** | **<0.0001** | **<0.0001** | **<0.0001** | **<0.0001** |
|  |  |  | HL | 0.89 | **<0.0001** | **<0.0001** | **<0.0001** | **<0.0001** | **<0.0001** |
|  |  |  | HH | **0.84*** | **<0.0001** | **<0.0001** | **<0.0001** | **<0.0001** | **<0.0001** |
|  |  |  | LL | 0.98 | **<0.0001** | **<0.0001** | **<0.0001** | **<0.0001** | **<0.0001** |
| Range | firstorder |  | LH | 0.91 | **<0.0001** | **<0.0001** | **<0.0001** | **<0.0001** | **<0.0001** |
|  |  |  | HL | 0.89 | **<0.0001** | **<0.0001** | **<0.0001** | **<0.0001** | **<0.0001** |
|  |  |  | HH | **0.83*** | **<0.0001** | **<0.0001** | **<0.0001** | **<0.0001** | **<0.0001** |
|  |  |  | LL | 0.99 | **<0.0001** | **<0.0001** | **<0.0001** | **<0.0001** | **<0.0001** |
| RobustMeanAbsolute  Deviation | firstorder |  | LH | 0.91 | **<0.0001** | **<0.0001** | **<0.0001** | **<0.0001** | **<0.0001** |
|  |  |  | HL | 0.95 | **<0.0001** | **<0.0001** | **<0.0001** | **<0.0001** | **<0.0001** |
|  |  |  | HH | **0.76*** | **<0.0001** | **<0.0001** | **<0.0001** | **<0.0001** | **<0.0001** |
|  |  |  | LL | 1.00 | **<0.0001** | **<0.0001** | **<0.0001** | **<0.0001** | **<0.0001** |
| RootMeanSquared | firstorder |  | LH | 1.00 | **0.01** | **<0.0001** | **<0.0001** | **<0.0001** | **<0.0001** |
|  |  |  | HL | 1.00 | **0.0002** | **<0.0001** | **<0.0001** | **<0.0001** | **<0.0001** |
|  |  |  | HH | 0.98 | 0.29 | **0.001** | **<0.0001** | **<0.0001** | **<0.0001** |
|  |  |  | LL | 1.00 | **<0.0001** | **<0.0001** | **<0.0001** | **<0.0001** | **<0.0001** |
| Skewness | firstorder |  | LH | 0.89 | 0.22 | **0.003** | **0.0001** | **<0.0001** | **<0.0001** |
|  |  |  | HL | 0.93 | 0.21 | **0.003** | **0.0001** | **<0.0001** | **<0.0001** |
|  |  |  | HH | **0.73*** | **0.047** | **<0.0001** | **<0.0001** | **<0.0001** | **<0.0001** |
|  |  |  | LL | 0.99 | **0.003** | **<0.0001** | **<0.0001** | **<0.0001** | **<0.0001** |
| TotalEnergy_modified | firstorder |  | LH | 1.00 | **0.01** | **<0.0001** | **<0.0001** | **<0.0001** | **<0.0001** |
|  |  |  | HL | 1.00 | **0.0002** | **<0.0001** | **<0.0001** | **<0.0001** | **<0.0001** |
|  |  |  | HH | 0.98 | 0.29 | **0.001** | **<0.0001** | **<0.0001** | **<0.0001** |
|  |  |  | LL | 1.00 | **<0.0001** | **<0.0001** | **<0.0001** | **<0.0001** | **<0.0001** |
| Uniformity | firstorder |  | LH | 0.92 | **<0.0001** | **<0.0001** | **<0.0001** | **<0.0001** | **<0.0001** |
|  |  |  | HL | 0.97 | **<0.0001** | **<0.0001** | **<0.0001** | **<0.0001** | **<0.0001** |
|  |  |  | HH | 0.92 | **0.0007** | **<0.0001** | **<0.0001** | **<0.0001** | **<0.0001** |
|  |  |  | LL | 0.99 | **<0.0001** | **<0.0001** | **<0.0001** | **<0.0001** | **<0.0001** |
| Variance | firstorder |  | LH | 0.96 | **0.006** | **<0.0001** | **<0.0001** | **<0.0001** | **<0.0001** |
|  |  |  | HL | 0.96 | **<0.0001** | **<0.0001** | **<0.0001** | **<0.0001** | **<0.0001** |
|  |  |  | HH | 0.92 | 0.52 | **0.004** | **<0.0001** | **<0.0001** | **<0.0001** |
|  |  |  | LL | 1.00 | **0.001** | **<0.0001** | **<0.0001** | **<0.0001** | **<0.0001** |
| Autocorrelation | texture | glcm1 | LH | 0.89 | **<0.0001** | **<0.0001** | **<0.0001** | **<0.0001** | **<0.0001** |
|  |  |  | HL | 0.88 | **<0.0001** | **<0.0001** | **<0.0001** | **<0.0001** | **<0.0001** |
|  |  |  | HH | **0.79*** | **<0.0001** | **<0.0001** | **<0.0001** | **<0.0001** | **<0.0001** |
|  |  |  | LL | 0.98 | **<0.0001** | **<0.0001** | **<0.0001** | **<0.0001** | **<0.0001** |
| ClusterProminence | texture | glcm1 | LH | 0.90 | **0.004** | **<0.0001** | **<0.0001** | **<0.0001** | **<0.0001** |
|  |  |  | HL | 0.91 | **0.0002** | **<0.0001** | **<0.0001** | **<0.0001** | **<0.0001** |
|  |  |  | HH | **0.83*** | **0.03** | **<0.0001** | **<0.0001** | **<0.0001** | **<0.0001** |
|  |  |  | LL | 1.00 | **<0.0001** | **<0.0001** | **<0.0001** | **<0.0001** | **<0.0001** |
| ClusterShade | texture | glcm1 | LH | 0.97 | 0.51 | 0.07 | **0.02** | **0.0005** | **<0.0001** |
|  |  |  | HL | 0.97 | 0.55 | **0.04** | **0.003** | **0.0001** | **<0.0001** |
|  |  |  | HH | **0.83*** | **0.01** | **<0.0001** | **<0.0001** | **<0.0001** | **<0.0001** |
|  |  |  | LL | 1.00 | **<0.0001** | **<0.0001** | **<0.0001** | **<0.0001** | **<0.0001** |
| ClusterTendency | texture | glcm1 | LH | 0.97 | **0.002** | **<0.0001** | **<0.0001** | **<0.0001** | **<0.0001** |
|  |  |  | HL | 0.97 | **<0.0001** | **<0.0001** | **<0.0001** | **<0.0001** | **<0.0001** |
|  |  |  | HH | 0.90 | **0.0005** | **<0.0001** | **<0.0001** | **<0.0001** | **<0.0001** |
|  |  |  | LL | 1.00 | **0.008** | **<0.0001** | **<0.0001** | **<0.0001** | **<0.0001** |
| Contrast | texture | glcm1 | LH | 0.94 | 0.11 | **<0.0001** | **<0.0001** | **<0.0001** | **<0.0001** |
|  |  |  | HL | 0.94 | **0.0002** | **<0.0001** | **<0.0001** | **<0.0001** | **<0.0001** |
|  |  |  | HH | 0.88 | **0.02** | **<0.0001** | **<0.0001** | **<0.0001** | **<0.0001** |
|  |  |  | LL | 0.99 | **0.0001** | **<0.0001** | **<0.0001** | **<0.0001** | **<0.0001** |
| Correlation | texture | glcm1 | LH | 0.89 | **0.01** | **0.0002** | **0.0001** | **0.0005** | **0.046** |
|  |  |  | HL | 0.90 | 0.38 | **0.0003** | **<0.0001** | **<0.0001** | **<0.0001** |
|  |  |  | HH | 0.95 | **<0.0001** | **<0.0001** | **<0.0001** | **<0.0001** | **<0.0001** |
|  |  |  | LL | 0.99 | **0.002** | **<0.0001** | **<0.0001** | **<0.0001** | **<0.0001** |
| DifferenceAverage | texture | glcm1 | LH | 0.96 | **0.004** | **<0.0001** | **<0.0001** | **<0.0001** | **<0.0001** |
|  |  |  | HL | 0.98 | 0.53 | 0.84 | 0.70 | 0.24 | **0.003** |
|  |  |  | HH | 0.91 | **0.05** | **<0.0001** | **<0.0001** | **<0.0001** | **<0.0001** |
|  |  |  | LL | 1.00 | **<0.0001** | **<0.0001** | **<0.0001** | **<0.0001** | **<0.0001** |
| DifferenceEntropy | texture | glcm1 | LH | 0.96 | **0.02** | **<0.0001** | **<0.0001** | **<0.0001** | **<0.0001** |
|  |  |  | HL | 0.98 | 0.70 | 0.86 | 0.41 | 0.07 | **<0.0001** |
|  |  |  | HH | 0.90 | **0.0005** | **<0.0001** | **<0.0001** | **<0.0001** | **<0.0001** |
|  |  |  | LL | 0.99 | **<0.0001** | **<0.0001** | **<0.0001** | **<0.0001** | **<0.0001** |
| DifferenceVariance | texture | glcm1 | LH | 0.91 | **0.0002** | **<0.0001** | **<0.0001** | **<0.0001** | **<0.0001** |
|  |  |  | HL | 0.90 | **<0.0001** | **<0.0001** | **<0.0001** | **<0.0001** | **<0.0001** |
|  |  |  | HH | 0.86 | **0.004** | **<0.0001** | **<0.0001** | **<0.0001** | **<0.0001** |
|  |  |  | LL | 0.98 | **<0.0001** | **<0.0001** | **<0.0001** | **<0.0001** | **<0.0001** |
| Id | texture | glcm1 | LH | 0.86 | **<0.0001** | **<0.0001** | **<0.0001** | **<0.0001** | **<0.0001** |
|  |  |  | HL | 0.94 | **<0.0001** | **<0.0001** | **<0.0001** | **<0.0001** | **<0.0001** |
|  |  |  | HH | 0.92 | 0.28 | **<0.0001** | **<0.0001** | **<0.0001** | **<0.0001** |
|  |  |  | LL | 0.95 | **<0.0001** | **<0.0001** | **<0.0001** | **<0.0001** | **<0.0001** |
| Idm | texture | glcm1 | LH | 0.87 | **<0.0001** | **<0.0001** | **<0.0001** | **<0.0001** | **<0.0001** |
|  |  |  | HL | 0.94 | **<0.0001** | **<0.0001** | **<0.0001** | **<0.0001** | **<0.0001** |
|  |  |  | HH | 0.92 | 0.19 | **<0.0001** | **<0.0001** | **<0.0001** | **<0.0001** |
|  |  |  | LL | 0.95 | **<0.0001** | **<0.0001** | **<0.0001** | **<0.0001** | **<0.0001** |
| Idmn | texture | glcm1 | LH | 0.93 | **<0.0001** | **<0.0001** | **<0.0001** | **<0.0001** | **<0.0001** |
|  |  |  | HL | 0.98 | **0.0002** | **<0.0001** | **<0.0001** | **<0.0001** | **<0.0001** |
|  |  |  | HH | **0.72*** | **<0.0001** | **<0.0001** | **<0.0001** | **<0.0001** | **<0.0001** |
|  |  |  | LL | 1.00 | **0.02** | **<0.0001** | **<0.0001** | **<0.0001** | **<0.0001** |
| Idn | texture | glcm1 | LH | 0.85 | **<0.0001** | **<0.0001** | **<0.0001** | **<0.0001** | **<0.0001** |
|  |  |  | HL | 0.94 | **<0.0001** | **<0.0001** | **<0.0001** | **<0.0001** | **<0.0001** |
|  |  |  | HH | **0.75*** | **<0.0001** | **<0.0001** | **<0.0001** | **<0.0001** | **<0.0001** |
|  |  |  | LL | 0.99 | **<0.0001** | **<0.0001** | **<0.0001** | **<0.0001** | **<0.0001** |
| Imc1 | texture | glcm1 | LH | **0.76*** | **<0.0001** | **<0.0001** | **<0.0001** | **<0.0001** | **<0.0001** |
|  |  |  | HL | **0.79*** | **<0.0001** | **<0.0001** | **<0.0001** | **<0.0001** | **<0.0001** |
|  |  |  | HH | 0.86 | **<0.0001** | **<0.0001** | **<0.0001** | **<0.0001** | **<0.0001** |
|  |  |  | LL | **0.79*** | **<0.0001** | **<0.0001** | **<0.0001** | **<0.0001** | **<0.0001** |
| Imc2 | texture | glcm1 | LH | 0.89 | **<0.0001** | **<0.0001** | **<0.0001** | **<0.0001** | **<0.0001** |
|  |  |  | HL | 0.93 | **<0.0001** | **<0.0001** | **<0.0001** | **<0.0001** | **<0.0001** |
|  |  |  | HH | 0.85 | **<0.0001** | **<0.0001** | **<0.0001** | **<0.0001** | **<0.0001** |
|  |  |  | LL | 0.89 | **<0.0001** | **<0.0001** | **<0.0001** | **<0.0001** | **<0.0001** |
| InverseVariance | texture | glcm1 | LH | **0.62*** | **<0.0001** | **<0.0001** | **<0.0001** | **<0.0001** | **<0.0001** |
|  |  |  | HL | **0.48*** | **<0.0001** | **<0.0001** | **<0.0001** | **<0.0001** | **<0.0001** |
|  |  |  | HH | **0.77*** | **0.0001** | **<0.0001** | **<0.0001** | **<0.0001** | **<0.0001** |
|  |  |  | LL | 0.98 | 0.85 | **0.002** | **<0.0001** | **<0.0001** | **<0.0001** |
| JointEnergy | texture | glcm1 | LH | 0.85 | **<0.0001** | **<0.0001** | **<0.0001** | **<0.0001** | **<0.0001** |
|  |  |  | HL | 0.93 | **<0.0001** | **<0.0001** | **<0.0001** | **<0.0001** | **<0.0001** |
|  |  |  | HH | 0.93 | **0.02** | **<0.0001** | **<0.0001** | **<0.0001** | **<0.0001** |
|  |  |  | LL | 0.93 | **<0.0001** | **<0.0001** | **<0.0001** | **<0.0001** | **<0.0001** |
| JointEntropy | texture | glcm1 | LH | 0.95 | **<0.0001** | **<0.0001** | **<0.0001** | **<0.0001** | **<0.0001** |
|  |  |  | HL | 0.98 | **0.0002** | **<0.0001** | **<0.0001** | **<0.0001** | **<0.0001** |
|  |  |  | HH | 0.92 | **0.0005** | **<0.0001** | **<0.0001** | **<0.0001** | **<0.0001** |
|  |  |  | LL | 0.99 | **<0.0001** | **<0.0001** | **<0.0001** | **<0.0001** | **<0.0001** |
| MaximumProbability | texture | glcm1 | LH | **0.80*** | **<0.0001** | **<0.0001** | **<0.0001** | **<0.0001** | **<0.0001** |
|  |  |  | HL | 0.88 | **<0.0001** | **<0.0001** | **<0.0001** | **<0.0001** | **<0.0001** |
|  |  |  | HH | 0.95 | 0.62 | **0.0004** | **<0.0001** | **<0.0001** | **<0.0001** |
|  |  |  | LL | 0.89 | **<0.0001** | **<0.0001** | **<0.0001** | **<0.0001** | **<0.0001** |
| MCC | texture | glcm1 | LH | **0.81*** | **<0.0001** | **<0.0001** | **<0.0001** | **<0.0001** | **<0.0001** |
|  |  |  | HL | **0.81*** | **<0.0001** | **<0.0001** | **<0.0001** | **<0.0001** | **<0.0001** |
|  |  |  | HH | **0.78*** | **<0.0001** | **<0.0001** | **<0.0001** | **<0.0001** | **<0.0001** |
|  |  |  | LL | 0.97 | 0.51 | 0.19 | 0.20 | 0.75 | 0.63 |
| SumAverage | texture | glcm1 | LH | 0.89 | **<0.0001** | **<0.0001** | **<0.0001** | **<0.0001** | **<0.0001** |
|  |  |  | HL | 0.88 | **<0.0001** | **<0.0001** | **<0.0001** | **<0.0001** | **<0.0001** |
|  |  |  | HH | **0.79*** | **<0.0001** | **<0.0001** | **<0.0001** | **<0.0001** | **<0.0001** |
|  |  |  | LL | 0.98 | **<0.0001** | **<0.0001** | **<0.0001** | **<0.0001** | **<0.0001** |
| SumEntropy | texture | glcm1 | LH | 0.97 | **<0.0001** | **<0.0001** | **<0.0001** | **<0.0001** | **<0.0001** |
|  |  |  | HL | 0.99 | **0.0007** | **<0.0001** | **<0.0001** | **<0.0001** | **<0.0001** |
|  |  |  | HH | 0.93 | **<0.0001** | **<0.0001** | **<0.0001** | **<0.0001** | **<0.0001** |
|  |  |  | LL | 1.00 | **<0.0001** | **<0.0001** | **<0.0001** | **<0.0001** | **<0.0001** |
| SumSquares | texture | glcm1 | LH | 0.96 | **0.01** | **<0.0001** | **<0.0001** | **<0.0001** | **<0.0001** |
|  |  |  | HL | 0.96 | **<0.0001** | **<0.0001** | **<0.0001** | **<0.0001** | **<0.0001** |
|  |  |  | HH | 0.89 | **0.003** | **<0.0001** | **<0.0001** | **<0.0001** | **<0.0001** |
|  |  |  | LL | 1.00 | **0.003** | **<0.0001** | **<0.0001** | **<0.0001** | **<0.0001** |
| Autocorrelation | texture | glcm4 | LH | 0.89 | **<0.0001** | **<0.0001** | **<0.0001** | **<0.0001** | **<0.0001** |
|  |  |  | HL | 0.88 | **<0.0001** | **<0.0001** | **<0.0001** | **<0.0001** | **<0.0001** |
|  |  |  | HH | **0.79*** | **<0.0001** | **<0.0001** | **<0.0001** | **<0.0001** | **<0.0001** |
|  |  |  | LL | 0.98 | **<0.0001** | **<0.0001** | **<0.0001** | **<0.0001** | **<0.0001** |
| ClusterProminence | texture | glcm4 | LH | 0.90 | **0.01** | **<0.0001** | **<0.0001** | **<0.0001** | **<0.0001** |
|  |  |  | HL | 0.90 | **0.002** | **<0.0001** | **<0.0001** | **<0.0001** | **<0.0001** |
|  |  |  | HH | **0.83*** | 0.08 | **<0.0001** | **<0.0001** | **<0.0001** | **<0.0001** |
|  |  |  | LL | 1.00 | **0.0004** | **<0.0001** | **<0.0001** | **<0.0001** | **<0.0001** |
| ClusterShade | texture | glcm4 | LH | 0.97 | 0.34 | **0.01** | **0.002** | **<0.0001** | **<0.0001** |
|  |  |  | HL | 0.97 | 0.22 | **0.001** | **<0.0001** | **<0.0001** | **<0.0001** |
|  |  |  | HH | **0.80*** | **0.03** | **<0.0001** | **<0.0001** | **<0.0001** | **<0.0001** |
|  |  |  | LL | 1.00 | **<0.0001** | **<0.0001** | **<0.0001** | **<0.0001** | **<0.0001** |
| ClusterTendency | texture | glcm4 | LH | 0.96 | **0.04** | **<0.0001** | **<0.0001** | **<0.0001** | **<0.0001** |
|  |  |  | HL | 0.96 | **0.0001** | **<0.0001** | **<0.0001** | **<0.0001** | **<0.0001** |
|  |  |  | HH | 0.90 | **0.01** | **<0.0001** | **<0.0001** | **<0.0001** | **<0.0001** |
|  |  |  | LL | 1.00 | **0.01** | **<0.0001** | **<0.0001** | **<0.0001** | **<0.0001** |
| Contrast | texture | glcm4 | LH | 0.97 | **0.05** | **<0.0001** | **<0.0001** | **<0.0001** | **<0.0001** |
|  |  |  | HL | 0.97 | **0.0001** | **<0.0001** | **<0.0001** | **<0.0001** | **<0.0001** |
|  |  |  | HH | 0.90 | **0.01** | **<0.0001** | **<0.0001** | **<0.0001** | **<0.0001** |
|  |  |  | LL | 1.00 | **0.001** | **<0.0001** | **<0.0001** | **<0.0001** | **<0.0001** |
| Correlation | texture | glcm4 | LH | 0.97 | **<0.0001** | **<0.0001** | **<0.0001** | **<0.0001** | **<0.0001** |
|  |  |  | HL | 0.97 | **<0.0001** | **<0.0001** | **<0.0001** | **<0.0001** | **<0.0001** |
|  |  |  | HH | 0.90 | **0.0002** | **<0.0001** | **<0.0001** | **<0.0001** | **<0.0001** |
|  |  |  | LL | 1.00 | **0.003** | **<0.0001** | **<0.0001** | **<0.0001** | **<0.0001** |
| DifferenceAverage | texture | glcm4 | LH | 0.98 | **0.01** | **<0.0001** | **<0.0001** | **<0.0001** | **0.0002** |
|  |  |  | HL | 0.99 | 0.96 | 0.15 | **0.009** | **<0.0001** | **<0.0001** |
|  |  |  | HH | 0.92 | **0.01** | **<0.0001** | **<0.0001** | **<0.0001** | **<0.0001** |
|  |  |  | LL | 1.00 | **<0.0001** | **<0.0001** | **<0.0001** | **<0.0001** | **<0.0001** |
| DifferenceEntropy | texture | glcm4 | LH | 0.98 | **0.007** | **<0.0001** | **<0.0001** | **<0.0001** | **<0.0001** |
|  |  |  | HL | 0.99 | 0.32 | 0.25 | 0.38 | 0.78 | 0.07 |
|  |  |  | HH | 0.92 | **0.0003** | **<0.0001** | **<0.0001** | **<0.0001** | **<0.0001** |
|  |  |  | LL | 1.00 | **<0.0001** | **<0.0001** | **<0.0001** | **<0.0001** | **<0.0001** |
| DifferenceVariance | texture | glcm4 | LH | 0.95 | **<0.0001** | **<0.0001** | **<0.0001** | **<0.0001** | **<0.0001** |
|  |  |  | HL | 0.95 | **<0.0001** | **<0.0001** | **<0.0001** | **<0.0001** | **<0.0001** |
|  |  |  | HH | 0.88 | **0.004** | **<0.0001** | **<0.0001** | **<0.0001** | **<0.0001** |
|  |  |  | LL | 1.00 | **<0.0001** | **<0.0001** | **<0.0001** | **<0.0001** | **<0.0001** |
| Id | texture | glcm4 | LH | 0.93 | **<0.0001** | **<0.0001** | **<0.0001** | **<0.0001** | **<0.0001** |
|  |  |  | HL | 0.98 | **<0.0001** | **<0.0001** | **<0.0001** | **<0.0001** | **<0.0001** |
|  |  |  | HH | 0.93 | **0.03** | **<0.0001** | **<0.0001** | **<0.0001** | **<0.0001** |
|  |  |  | LL | 0.99 | **<0.0001** | **<0.0001** | **<0.0001** | **<0.0001** | **<0.0001** |
| Idm | texture | glcm4 | LH | 0.92 | **<0.0001** | **<0.0001** | **<0.0001** | **<0.0001** | **<0.0001** |
|  |  |  | HL | 0.98 | **<0.0001** | **<0.0001** | **<0.0001** | **<0.0001** | **<0.0001** |
|  |  |  | HH | 0.93 | **0.02** | **<0.0001** | **<0.0001** | **<0.0001** | **<0.0001** |
|  |  |  | LL | 0.98 | **<0.0001** | **<0.0001** | **<0.0001** | **<0.0001** | **<0.0001** |
| Idmn | texture | glcm4 | LH | 0.95 | **<0.0001** | **<0.0001** | **<0.0001** | **<0.0001** | **<0.0001** |
|  |  |  | HL | 0.98 | **<0.0001** | **<0.0001** | **<0.0001** | **<0.0001** | **<0.0001** |
|  |  |  | HH | **0.74*** | **<0.0001** | **<0.0001** | **<0.0001** | **<0.0001** | **<0.0001** |
|  |  |  | LL | 1.00 | 0.11 | **0.006** | **<0.0001** | **<0.0001** | **<0.0001** |
| Idn | texture | glcm4 | LH | 0.90 | **<0.0001** | **<0.0001** | **<0.0001** | **<0.0001** | **<0.0001** |
|  |  |  | HL | 0.96 | **<0.0001** | **<0.0001** | **<0.0001** | **<0.0001** | **<0.0001** |
|  |  |  | HH | **0.79*** | **<0.0001** | **<0.0001** | **<0.0001** | **<0.0001** | **<0.0001** |
|  |  |  | LL | 1.00 | **<0.0001** | **<0.0001** | **<0.0001** | **<0.0001** | **<0.0001** |
| Imc1 | texture | glcm4 | LH | **0.82*** | **<0.0001** | **<0.0001** | **<0.0001** | **<0.0001** | **<0.0001** |
|  |  |  | HL | 0.89 | **<0.0001** | **<0.0001** | **<0.0001** | **<0.0001** | **<0.0001** |
|  |  |  | HH | 0.85 | **<0.0001** | **<0.0001** | **<0.0001** | **<0.0001** | **<0.0001** |
|  |  |  | LL | 0.96 | **<0.0001** | **<0.0001** | **<0.0001** | **<0.0001** | **<0.0001** |
| Imc2 | texture | glcm4 | LH | 0.90 | **<0.0001** | **<0.0001** | **<0.0001** | **<0.0001** | **<0.0001** |
|  |  |  | HL | 0.95 | **<0.0001** | **<0.0001** | **<0.0001** | **<0.0001** | **<0.0001** |
|  |  |  | HH | 0.86 | **<0.0001** | **<0.0001** | **<0.0001** | **<0.0001** | **<0.0001** |
|  |  |  | LL | 0.98 | **<0.0001** | **<0.0001** | **<0.0001** | **<0.0001** | **<0.0001** |
| InverseVariance | texture | glcm4 | LH | 0.97 | 0.08 | **<0.0001** | **<0.0001** | **<0.0001** | **<0.0001** |
|  |  |  | HL | 0.98 | **<0.0001** | **<0.0001** | **<0.0001** | **<0.0001** | **<0.0001** |
|  |  |  | HH | **0.71*** | **0.003** | **<0.0001** | **<0.0001** | **<0.0001** | **<0.0001** |
|  |  |  | LL | 0.99 | **<0.0001** | **<0.0001** | **<0.0001** | **<0.0001** | **<0.0001** |
| JointEnergy | texture | glcm4 | LH | 0.86 | **<0.0001** | **<0.0001** | **<0.0001** | **<0.0001** | **<0.0001** |
|  |  |  | HL | 0.95 | **<0.0001** | **<0.0001** | **<0.0001** | **<0.0001** | **<0.0001** |
|  |  |  | HH | 0.93 | **0.04** | **<0.0001** | **<0.0001** | **<0.0001** | **<0.0001** |
|  |  |  | LL | 0.97 | **<0.0001** | **<0.0001** | **<0.0001** | **<0.0001** | **<0.0001** |
| JointEntropy | texture | glcm4 | LH | 0.95 | **<0.0001** | **<0.0001** | **<0.0001** | **<0.0001** | **<0.0001** |
|  |  |  | HL | 0.98 | **<0.0001** | **<0.0001** | **<0.0001** | **<0.0001** | **<0.0001** |
|  |  |  | HH | 0.93 | **0.004** | **<0.0001** | **<0.0001** | **<0.0001** | **<0.0001** |
|  |  |  | LL | 1.00 | **<0.0001** | **<0.0001** | **<0.0001** | **<0.0001** | **<0.0001** |
| MaximumProbability | texture | glcm4 | LH | **0.82*** | **<0.0001** | **<0.0001** | **<0.0001** | **<0.0001** | **<0.0001** |
|  |  |  | HL | 0.93 | **<0.0001** | **<0.0001** | **<0.0001** | **<0.0001** | **<0.0001** |
|  |  |  | HH | 0.95 | 0.95 | **0.04** | **0.0001** | **<0.0001** | **<0.0001** |
|  |  |  | LL | 0.95 | **<0.0001** | **<0.0001** | **<0.0001** | **<0.0001** | **<0.0001** |
| MCC | texture | glcm4 | LH | **0.84*** | **<0.0001** | **<0.0001** | **<0.0001** | **<0.0001** | **<0.0001** |
|  |  |  | HL | 0.92 | **<0.0001** | **<0.0001** | **<0.0001** | **<0.0001** | **<0.0001** |
|  |  |  | HH | 0.86 | **<0.0001** | **<0.0001** | **<0.0001** | **<0.0001** | **<0.0001** |
|  |  |  | LL | 0.99 | **0.001** | **<0.0001** | **<0.0001** | **<0.0001** | **<0.0001** |
| SumAverage | texture | glcm4 | LH | 0.89 | **<0.0001** | **<0.0001** | **<0.0001** | **<0.0001** | **<0.0001** |
|  |  |  | HL | 0.88 | **<0.0001** | **<0.0001** | **<0.0001** | **<0.0001** | **<0.0001** |
|  |  |  | HH | **0.79*** | **<0.0001** | **<0.0001** | **<0.0001** | **<0.0001** | **<0.0001** |
|  |  |  | LL | 0.98 | **<0.0001** | **<0.0001** | **<0.0001** | **<0.0001** | **<0.0001** |
| SumEntropy | texture | glcm4 | LH | 0.97 | **0.0002** | **<0.0001** | **<0.0001** | **<0.0001** | **<0.0001** |
|  |  |  | HL | 0.99 | **0.04** | **0.001** | **0.0008** | **0.001** | 0.055 |
|  |  |  | HH | 0.92 | **0.001** | **<0.0001** | **<0.0001** | **<0.0001** | **<0.0001** |
|  |  |  | LL | 1.00 | **<0.0001** | **<0.0001** | **<0.0001** | **<0.0001** | **<0.0001** |
| SumSquares | texture | glcm4 | LH | 0.97 | **0.049** | **<0.0001** | **<0.0001** | **<0.0001** | **<0.0001** |
|  |  |  | HL | 0.97 | **0.0001** | **<0.0001** | **<0.0001** | **<0.0001** | **<0.0001** |
|  |  |  | HH | 0.90 | **0.01** | **<0.0001** | **<0.0001** | **<0.0001** | **<0.0001** |
|  |  |  | LL | 1.00 | **0.004** | **<0.0001** | **<0.0001** | **<0.0001** | **<0.0001** |
| Autocorrelation | texture | glcm7 | LH | 0.89 | **<0.0001** | **<0.0001** | **<0.0001** | **<0.0001** | **<0.0001** |
|  |  |  | HL | 0.88 | **<0.0001** | **<0.0001** | **<0.0001** | **<0.0001** | **<0.0001** |
|  |  |  | HH | **0.79*** | **<0.0001** | **<0.0001** | **<0.0001** | **<0.0001** | **<0.0001** |
|  |  |  | LL | 0.98 | **<0.0001** | **<0.0001** | **<0.0001** | **<0.0001** | **<0.0001** |
| ClusterProminence | texture | glcm7 | LH | 0.90 | **0.01** | **<0.0001** | **<0.0001** | **<0.0001** | **<0.0001** |
|  |  |  | HL | 0.91 | **0.002** | **<0.0001** | **<0.0001** | **<0.0001** | **<0.0001** |
|  |  |  | HH | **0.83*** | 0.08 | **<0.0001** | **<0.0001** | **<0.0001** | **<0.0001** |
|  |  |  | LL | 1.00 | **0.0002** | **<0.0001** | **<0.0001** | **<0.0001** | **<0.0001** |
| ClusterShade | texture | glcm7 | LH | 0.97 | 0.69 | 0.20 | 0.11 | **0.008** | **<0.0001** |
|  |  |  | HL | 0.97 | 0.52 | 0.06 | **0.005** | **0.0002** | **<0.0001** |
|  |  |  | HH | **0.81*** | **0.049** | **<0.0001** | **<0.0001** | **<0.0001** | **<0.0001** |
|  |  |  | LL | 1.00 | **<0.0001** | **<0.0001** | **<0.0001** | **<0.0001** | **<0.0001** |
| ClusterTendency | texture | glcm7 | LH | 0.97 | 0.06 | **<0.0001** | **<0.0001** | **<0.0001** | **<0.0001** |
|  |  |  | HL | 0.97 | **0.0002** | **<0.0001** | **<0.0001** | **<0.0001** | **<0.0001** |
|  |  |  | HH | 0.89 | **0.01** | **<0.0001** | **<0.0001** | **<0.0001** | **<0.0001** |
|  |  |  | LL | 1.00 | **0.007** | **<0.0001** | **<0.0001** | **<0.0001** | **<0.0001** |
| Contrast | texture | glcm7 | LH | 0.97 | 0.06 | **<0.0001** | **<0.0001** | **<0.0001** | **<0.0001** |
|  |  |  | HL | 0.97 | **0.0002** | **<0.0001** | **<0.0001** | **<0.0001** | **<0.0001** |
|  |  |  | HH | 0.89 | **0.01** | **<0.0001** | **<0.0001** | **<0.0001** | **<0.0001** |
|  |  |  | LL | 1.00 | **0.003** | **<0.0001** | **<0.0001** | **<0.0001** | **<0.0001** |
| Correlation | texture | glcm7 | LH | 0.98 | 0.48 | **0.03** | **0.009** | **0.008** | **0.03** |
|  |  |  | HL | 0.99 | 0.70 | 0.08 | **0.05** | **0.01** | **<0.0001** |
|  |  |  | HH | **0.82*** | 0.59 | 0.75 | 0.13 | 0.42 | 0.64 |
|  |  |  | LL | 1.00 | **0.03** | **<0.0001** | **<0.0001** | **<0.0001** | **<0.0001** |
| DifferenceAverage | texture | glcm7 | LH | 0.98 | **0.02** | **0.0002** | **<0.0001** | **0.0001** | **0.005** |
|  |  |  | HL | 0.99 | 0.79 | 0.06 | **0.002** | **<0.0001** | **<0.0001** |
|  |  |  | HH | 0.91 | **0.01** | **<0.0001** | **<0.0001** | **<0.0001** | **<0.0001** |
|  |  |  | LL | 1.00 | **<0.0001** | **<0.0001** | **<0.0001** | **<0.0001** | **<0.0001** |
| DifferenceEntropy | texture | glcm7 | LH | 0.98 | **0.02** | **<0.0001** | **<0.0001** | **<0.0001** | **<0.0001** |
|  |  |  | HL | 0.99 | 0.61 | 0.81 | 0.83 | 0.32 | **0.0010** |
|  |  |  | HH | 0.92 | **0.0003** | **<0.0001** | **<0.0001** | **<0.0001** | **<0.0001** |
|  |  |  | LL | 1.00 | **<0.0001** | **<0.0001** | **<0.0001** | **<0.0001** | **<0.0001** |
| DifferenceVariance | texture | glcm7 | LH | 0.94 | **<0.0001** | **<0.0001** | **<0.0001** | **<0.0001** | **<0.0001** |
|  |  |  | HL | 0.94 | **<0.0001** | **<0.0001** | **<0.0001** | **<0.0001** | **<0.0001** |
|  |  |  | HH | 0.88 | **0.005** | **<0.0001** | **<0.0001** | **<0.0001** | **<0.0001** |
|  |  |  | LL | 1.00 | **<0.0001** | **<0.0001** | **<0.0001** | **<0.0001** | **<0.0001** |
| Id | texture | glcm7 | LH | 0.93 | **<0.0001** | **<0.0001** | **<0.0001** | **<0.0001** | **<0.0001** |
|  |  |  | HL | 0.98 | **<0.0001** | **<0.0001** | **<0.0001** | **<0.0001** | **<0.0001** |
|  |  |  | HH | 0.93 | **0.03** | **<0.0001** | **<0.0001** | **<0.0001** | **<0.0001** |
|  |  |  | LL | 0.99 | **<0.0001** | **<0.0001** | **<0.0001** | **<0.0001** | **<0.0001** |
| Idm | texture | glcm7 | LH | 0.93 | **<0.0001** | **<0.0001** | **<0.0001** | **<0.0001** | **<0.0001** |
|  |  |  | HL | 0.98 | **<0.0001** | **<0.0001** | **<0.0001** | **<0.0001** | **<0.0001** |
|  |  |  | HH | 0.92 | **0.02** | **<0.0001** | **<0.0001** | **<0.0001** | **<0.0001** |
|  |  |  | LL | 0.99 | **<0.0001** | **<0.0001** | **<0.0001** | **<0.0001** | **<0.0001** |
| Idmn | texture | glcm7 | LH | 0.95 | **<0.0001** | **<0.0001** | **<0.0001** | **<0.0001** | **<0.0001** |
|  |  |  | HL | 0.98 | **<0.0001** | **<0.0001** | **<0.0001** | **<0.0001** | **<0.0001** |
|  |  |  | HH | **0.74*** | **<0.0001** | **<0.0001** | **<0.0001** | **<0.0001** | **<0.0001** |
|  |  |  | LL | 1.00 | **0.01** | **<0.0001** | **<0.0001** | **<0.0001** | **<0.0001** |
| Idn | texture | glcm7 | LH | 0.91 | **<0.0001** | **<0.0001** | **<0.0001** | **<0.0001** | **<0.0001** |
|  |  |  | HL | 0.97 | **<0.0001** | **<0.0001** | **<0.0001** | **<0.0001** | **<0.0001** |
|  |  |  | HH | **0.79*** | **<0.0001** | **<0.0001** | **<0.0001** | **<0.0001** | **<0.0001** |
|  |  |  | LL | 1.00 | **<0.0001** | **<0.0001** | **<0.0001** | **<0.0001** | **<0.0001** |
| Imc1 | texture | glcm7 | LH | 0.89 | **<0.0001** | **<0.0001** | **<0.0001** | **<0.0001** | **<0.0001** |
|  |  |  | HL | 0.94 | **<0.0001** | **<0.0001** | **<0.0001** | **<0.0001** | **<0.0001** |
|  |  |  | HH | 0.91 | **<0.0001** | **<0.0001** | **<0.0001** | **<0.0001** | **<0.0001** |
|  |  |  | LL | 0.99 | **<0.0001** | **<0.0001** | **<0.0001** | **<0.0001** | **<0.0001** |
| Imc2 | texture | glcm7 | LH | 0.94 | **<0.0001** | **<0.0001** | **<0.0001** | **<0.0001** | **<0.0001** |
|  |  |  | HL | 0.97 | **<0.0001** | **<0.0001** | **<0.0001** | **<0.0001** | **<0.0001** |
|  |  |  | HH | 0.91 | **<0.0001** | **<0.0001** | **<0.0001** | **<0.0001** | **<0.0001** |
|  |  |  | LL | 0.99 | **<0.0001** | **<0.0001** | **<0.0001** | **<0.0001** | **<0.0001** |
| InverseVariance | texture | glcm7 | LH | 0.98 | 0.16 | **<0.0001** | **<0.0001** | **<0.0001** | **<0.0001** |
|  |  |  | HL | 0.98 | **<0.0001** | **<0.0001** | **<0.0001** | **<0.0001** | **<0.0001** |
|  |  |  | HH | **0.68*** | **0.009** | **<0.0001** | **<0.0001** | **<0.0001** | **<0.0001** |
|  |  |  | LL | 0.99 | **<0.0001** | **<0.0001** | **<0.0001** | **<0.0001** | **<0.0001** |
| JointEnergy | texture | glcm7 | LH | 0.87 | **<0.0001** | **<0.0001** | **<0.0001** | **<0.0001** | **<0.0001** |
|  |  |  | HL | 0.95 | **<0.0001** | **<0.0001** | **<0.0001** | **<0.0001** | **<0.0001** |
|  |  |  | HH | 0.93 | **0.04** | **<0.0001** | **<0.0001** | **<0.0001** | **<0.0001** |
|  |  |  | LL | 0.98 | **<0.0001** | **<0.0001** | **<0.0001** | **<0.0001** | **<0.0001** |
| JointEntropy | texture | glcm7 | LH | 0.95 | **<0.0001** | **<0.0001** | **<0.0001** | **<0.0001** | **<0.0001** |
|  |  |  | HL | 0.98 | **<0.0001** | **<0.0001** | **<0.0001** | **<0.0001** | **<0.0001** |
|  |  |  | HH | 0.93 | **0.005** | **<0.0001** | **<0.0001** | **<0.0001** | **<0.0001** |
|  |  |  | LL | 1.00 | **<0.0001** | **<0.0001** | **<0.0001** | **<0.0001** | **<0.0001** |
| MaximumProbability | texture | glcm7 | LH | **0.84*** | **<0.0001** | **<0.0001** | **<0.0001** | **<0.0001** | **<0.0001** |
|  |  |  | HL | 0.94 | **<0.0001** | **<0.0001** | **<0.0001** | **<0.0001** | **<0.0001** |
|  |  |  | HH | 0.94 | 0.99 | 0.07 | **0.0005** | **<0.0001** | **<0.0001** |
|  |  |  | LL | 0.96 | **<0.0001** | **<0.0001** | **<0.0001** | **<0.0001** | **<0.0001** |
| MCC | texture | glcm7 | LH | 0.89 | **<0.0001** | **<0.0001** | **<0.0001** | **<0.0001** | **<0.0001** |
|  |  |  | HL | 0.95 | **<0.0001** | **<0.0001** | **<0.0001** | **<0.0001** | **<0.0001** |
|  |  |  | HH | 0.87 | **<0.0001** | **<0.0001** | **<0.0001** | **<0.0001** | **<0.0001** |
|  |  |  | LL | 0.99 | **<0.0001** | **<0.0001** | **<0.0001** | **<0.0001** | **<0.0001** |
| SumAverage | texture | glcm7 | LH | 0.89 | **<0.0001** | **<0.0001** | **<0.0001** | **<0.0001** | **<0.0001** |
|  |  |  | HL | 0.88 | **<0.0001** | **<0.0001** | **<0.0001** | **<0.0001** | **<0.0001** |
|  |  |  | HH | **0.79*** | **<0.0001** | **<0.0001** | **<0.0001** | **<0.0001** | **<0.0001** |
|  |  |  | LL | 0.98 | **<0.0001** | **<0.0001** | **<0.0001** | **<0.0001** | **<0.0001** |
| SumEntropy | texture | glcm7 | LH | 0.97 | **0.0003** | **<0.0001** | **<0.0001** | **<0.0001** | **<0.0001** |
|  |  |  | HL | 0.99 | 0.06 | **0.005** | **0.004** | **0.01** | 0.26 |
|  |  |  | HH | 0.92 | **0.001** | **<0.0001** | **<0.0001** | **<0.0001** | **<0.0001** |
|  |  |  | LL | 1.00 | **<0.0001** | **<0.0001** | **<0.0001** | **<0.0001** | **<0.0001** |
| SumSquares | texture | glcm7 | LH | 0.97 | 0.06 | **<0.0001** | **<0.0001** | **<0.0001** | **<0.0001** |
|  |  |  | HL | 0.97 | **0.0002** | **<0.0001** | **<0.0001** | **<0.0001** | **<0.0001** |
|  |  |  | HH | 0.89 | **0.01** | **<0.0001** | **<0.0001** | **<0.0001** | **<0.0001** |
|  |  |  | LL | 1.00 | **0.005** | **<0.0001** | **<0.0001** | **<0.0001** | **<0.0001** |
| DependenceEntropy | texture | gldm | LH | 0.98 | **0.007** | **<0.0001** | **<0.0001** | **<0.0001** | **<0.0001** |
|  |  |  | HL | 0.99 | 0.27 | 0.49 | 0.86 | 0.07 | **<0.0001** |
|  |  |  | HH | 0.92 | **<0.0001** | **<0.0001** | **<0.0001** | **<0.0001** | **<0.0001** |
|  |  |  | LL | 0.99 | **<0.0001** | **<0.0001** | **<0.0001** | **<0.0001** | **<0.0001** |
| DependenceNon  UniformityNormalized | texture | gldm | LH | **0.72*** | **<0.0001** | **<0.0001** | **<0.0001** | **<0.0001** | **<0.0001** |
|  |  |  | HL | 0.87 | **<0.0001** | **<0.0001** | **<0.0001** | **<0.0001** | **<0.0001** |
|  |  |  | HH | 0.93 | **0.001** | **<0.0001** | **<0.0001** | **<0.0001** | **<0.0001** |
|  |  |  | LL | 0.96 | **<0.0001** | **<0.0001** | **<0.0001** | **<0.0001** | **<0.0001** |
| DependenceVariance | texture | gldm | LH | **0.53*** | **<0.0001** | **<0.0001** | **<0.0001** | **<0.0001** | **<0.0001** |
|  |  |  | HL | **0.60*** | **<0.0001** | **<0.0001** | **<0.0001** | **<0.0001** | **<0.0001** |
|  |  |  | HH | 0.91 | **0.001** | **<0.0001** | **<0.0001** | **<0.0001** | **<0.0001** |
|  |  |  | LL | **0.67*** | **<0.0001** | **<0.0001** | **<0.0001** | **<0.0001** | **<0.0001** |
| GrayLevelNon  Uniformity_modified | texture | gldm | LH | 0.92 | **<0.0001** | **<0.0001** | **<0.0001** | **<0.0001** | **<0.0001** |
|  |  |  | HL | 0.97 | **<0.0001** | **<0.0001** | **<0.0001** | **<0.0001** | **<0.0001** |
|  |  |  | HH | 0.92 | **0.0007** | **<0.0001** | **<0.0001** | **<0.0001** | **<0.0001** |
|  |  |  | LL | 0.99 | **<0.0001** | **<0.0001** | **<0.0001** | **<0.0001** | **<0.0001** |
| GrayLevelVariance | texture | gldm | LH | 0.96 | **0.004** | **<0.0001** | **<0.0001** | **<0.0001** | **<0.0001** |
|  |  |  | HL | 0.96 | **<0.0001** | **<0.0001** | **<0.0001** | **<0.0001** | **<0.0001** |
|  |  |  | HH | 0.88 | **0.001** | **<0.0001** | **<0.0001** | **<0.0001** | **<0.0001** |
|  |  |  | LL | 1.00 | **0.0009** | **<0.0001** | **<0.0001** | **<0.0001** | **<0.0001** |
| HighGrayLevel  Emphasis | texture | gldm | LH | 0.89 | **<0.0001** | **<0.0001** | **<0.0001** | **<0.0001** | **<0.0001** |
|  |  |  | HL | 0.88 | **<0.0001** | **<0.0001** | **<0.0001** | **<0.0001** | **<0.0001** |
|  |  |  | HH | **0.79*** | **<0.0001** | **<0.0001** | **<0.0001** | **<0.0001** | **<0.0001** |
|  |  |  | LL | 0.98 | **<0.0001** | **<0.0001** | **<0.0001** | **<0.0001** | **<0.0001** |
| LargeDependence  Emphasis | texture | gldm | LH | **0.72*** | **<0.0001** | **<0.0001** | **<0.0001** | **<0.0001** | **<0.0001** |
|  |  |  | HL | **0.81*** | **<0.0001** | **<0.0001** | **<0.0001** | **<0.0001** | **<0.0001** |
|  |  |  | HH | 0.93 | 0.96 | 0.17 | **0.04** | **0.02** | 0.41 |
|  |  |  | LL | **0.81*** | **<0.0001** | **<0.0001** | **<0.0001** | **<0.0001** | **<0.0001** |
| LargeDependence  HighGrayLevel  Emphasis | texture | gldm | LH | **0.74*** | **<0.0001** | **<0.0001** | **<0.0001** | **<0.0001** | **<0.0001** |
|  |  |  | HL | **0.73*** | **<0.0001** | **<0.0001** | **<0.0001** | **<0.0001** | **<0.0001** |
|  |  |  | HH | **0.77*** | **<0.0001** | **<0.0001** | **<0.0001** | **<0.0001** | **<0.0001** |
|  |  |  | LL | **0.82*** | **<0.0001** | **<0.0001** | **<0.0001** | **<0.0001** | **<0.0001** |
| LargeDependenceLowGrayLevelEmphasis | texture | gldm | LH | 0.96 | 0.09 | **0.05** | **0.0003** | **<0.0001** | **<0.0001** |
|  |  |  | HL | 0.94 | **0.01** | **<0.0001** | **<0.0001** | **<0.0001** | **<0.0001** |
|  |  |  | HH | **0.72*** | **<0.0001** | **<0.0001** | **<0.0001** | **<0.0001** | **<0.0001** |
|  |  |  | LL | 0.90 | 0.11 | **0.03** | **0.006** | **0.02** | **0.0002** |
| LowGrayLevel  Emphasis | texture | gldm | LH | 0.85 | **<0.0001** | **<0.0001** | **<0.0001** | **<0.0001** | **<0.0001** |
|  |  |  | HL | 0.86 | **<0.0001** | **<0.0001** | **<0.0001** | **<0.0001** | **<0.0001** |
|  |  |  | HH | **0.69*** | **<0.0001** | **<0.0001** | **<0.0001** | **<0.0001** | **<0.0001** |
|  |  |  | LL | 0.94 | 0.83 | 0.12 | **0.01** | **0.001** | **0.003** |
| SmallDependence  Emphasis | texture | gldm | LH | 0.94 | **<0.0001** | **<0.0001** | **<0.0001** | **<0.0001** | **<0.0001** |
|  |  |  | HL | 0.98 | **0.001** | **<0.0001** | **<0.0001** | **<0.0001** | **<0.0001** |
|  |  |  | HH | 0.92 | 0.34 | **<0.0001** | **<0.0001** | **<0.0001** | **<0.0001** |
|  |  |  | LL | 0.98 | **<0.0001** | **<0.0001** | **<0.0001** | **<0.0001** | **<0.0001** |
| SmallDependenceHighGrayLevelEmphasis | texture | gldm | LH | 0.94 | **0.005** | **<0.0001** | **<0.0001** | **<0.0001** | **<0.0001** |
|  |  |  | HL | 0.94 | **0.003** | **<0.0001** | **<0.0001** | **<0.0001** | **<0.0001** |
|  |  |  | HH | **0.82*** | **0.002** | **<0.0001** | **<0.0001** | **<0.0001** | **<0.0001** |
|  |  |  | LL | 0.98 | **<0.0001** | **<0.0001** | **<0.0001** | **<0.0001** | **<0.0001** |
| SmallDependenceLowGrayLevelEmphasis | texture | gldm | LH | **0.84*** | **<0.0001** | **<0.0001** | **<0.0001** | **<0.0001** | **<0.0001** |
|  |  |  | HL | 0.90 | **<0.0001** | **<0.0001** | **<0.0001** | **<0.0001** | **<0.0001** |
|  |  |  | HH | **0.72*** | **<0.0001** | **<0.0001** | **<0.0001** | **<0.0001** | **<0.0001** |
|  |  |  | LL | 0.96 | 0.34 | 0.07 | **0.007** | **0.0007** | **0.006** |
| GrayLevelNon  UniformityNormalized | texture | glrlm | LH | 0.95 | **<0.0001** | **<0.0001** | **<0.0001** | **<0.0001** | **<0.0001** |
|  |  |  | HL | 0.98 | **0.003** | **<0.0001** | **<0.0001** | **<0.0001** | 0.06 |
|  |  |  | HH | 0.92 | **0.0005** | **<0.0001** | **<0.0001** | **<0.0001** | **<0.0001** |
|  |  |  | LL | 1.00 | **0.02** | **0.006** | **0.02** | 0.18 | 0.06 |
| GrayLevelVariance | texture | glrlm | LH | 0.92 | **<0.0001** | **<0.0001** | **<0.0001** | **<0.0001** | **<0.0001** |
|  |  |  | HL | 0.92 | **<0.0001** | **<0.0001** | **<0.0001** | **<0.0001** | **<0.0001** |
|  |  |  | HH | 0.88 | **0.0006** | **<0.0001** | **<0.0001** | **<0.0001** | **<0.0001** |
|  |  |  | LL | 1.00 | **<0.0001** | **<0.0001** | **<0.0001** | **<0.0001** | **<0.0001** |
| HighGrayLevelRun  Emphasis | texture | glrlm | LH | 0.89 | **<0.0001** | **<0.0001** | **<0.0001** | **<0.0001** | **<0.0001** |
|  |  |  | HL | 0.88 | **<0.0001** | **<0.0001** | **<0.0001** | **<0.0001** | **<0.0001** |
|  |  |  | HH | **0.79*** | **<0.0001** | **<0.0001** | **<0.0001** | **<0.0001** | **<0.0001** |
|  |  |  | LL | 0.99 | **0.0001** | **<0.0001** | **<0.0001** | **<0.0001** | **<0.0001** |
| LongRunEmphasis | texture | glrlm | LH | **0.69*** | **<0.0001** | **<0.0001** | **<0.0001** | **<0.0001** | **<0.0001** |
|  |  |  | HL | **0.78*** | **<0.0001** | **<0.0001** | **<0.0001** | **<0.0001** | **<0.0001** |
|  |  |  | HH | 0.95 | 0.74 | 0.35 | 0.07 | **0.03** | 0.15 |
|  |  |  | LL | **0.77*** | **<0.0001** | **<0.0001** | **<0.0001** | **<0.0001** | **<0.0001** |
| LongRunHighGray  LevelEmphasis | texture | glrlm | LH | **0.69*** | **<0.0001** | **<0.0001** | **<0.0001** | **<0.0001** | **<0.0001** |
|  |  |  | HL | **0.69*** | **<0.0001** | **<0.0001** | **<0.0001** | **<0.0001** | **<0.0001** |
|  |  |  | HH | **0.77*** | **<0.0001** | **<0.0001** | **<0.0001** | **<0.0001** | **<0.0001** |
|  |  |  | LL | **0.81*** | **<0.0001** | **<0.0001** | **<0.0001** | **<0.0001** | **<0.0001** |
| LongRunLowGray  LevelEmphasis | texture | glrlm | LH | 0.96 | 0.46 | 0.53 | 0.81 | 0.78 | 0.17 |
|  |  |  | HL | 0.94 | 0.20 | **0.003** | **0.0004** | **0.0004** | **<0.0001** |
|  |  |  | HH | **0.72*** | **<0.0001** | **<0.0001** | **<0.0001** | **<0.0001** | **<0.0001** |
|  |  |  | LL | 0.92 | 0.15 | **0.04** | **0.03** | **0.01** | **<0.0001** |
| LowGrayLevelRun  Emphasis | texture | glrlm | LH | 0.86 | **<0.0001** | **<0.0001** | **<0.0001** | **<0.0001** | **<0.0001** |
|  |  |  | HL | 0.87 | **<0.0001** | **<0.0001** | **<0.0001** | **<0.0001** | **<0.0001** |
|  |  |  | HH | **0.69*** | **<0.0001** | **<0.0001** | **<0.0001** | **<0.0001** | **<0.0001** |
|  |  |  | LL | 0.94 | 0.88 | 0.62 | 0.21 | 0.11 | 0.58 |
| RunEntropy | texture | glrlm | LH | 0.86 | **<0.0001** | **<0.0001** | **<0.0001** | **<0.0001** | **<0.0001** |
|  |  |  | HL | 0.85 | **<0.0001** | **<0.0001** | **<0.0001** | **<0.0001** | **<0.0001** |
|  |  |  | HH | 0.86 | **<0.0001** | **<0.0001** | **<0.0001** | **<0.0001** | **<0.0001** |
|  |  |  | LL | 0.96 | **<0.0001** | **<0.0001** | **<0.0001** | **<0.0001** | **<0.0001** |
| RunLengthNon  UniformityNormalized | texture | glrlm | LH | **0.84*** | **<0.0001** | **<0.0001** | **<0.0001** | **<0.0001** | **<0.0001** |
|  |  |  | HL | 0.92 | **<0.0001** | **<0.0001** | **<0.0001** | **<0.0001** | **<0.0001** |
|  |  |  | HH | 0.92 | 0.28 | **0.0001** | **<0.0001** | **<0.0001** | **<0.0001** |
|  |  |  | LL | 0.94 | **<0.0001** | **<0.0001** | **<0.0001** | **<0.0001** | **<0.0001** |
| RunPercentage | texture | glrlm | LH | **0.79*** | **<0.0001** | **<0.0001** | **<0.0001** | **<0.0001** | **<0.0001** |
|  |  |  | HL | 0.87 | **<0.0001** | **<0.0001** | **<0.0001** | **<0.0001** | **<0.0001** |
|  |  |  | HH | 0.94 | 0.69 | **0.01** | **0.0002** | **<0.0001** | **<0.0001** |
|  |  |  | LL | 0.88 | **<0.0001** | **<0.0001** | **<0.0001** | **<0.0001** | **<0.0001** |
| RunVariance | texture | glrlm | LH | **0.62*** | **<0.0001** | **<0.0001** | **<0.0001** | **<0.0001** | **<0.0001** |
|  |  |  | HL | **0.70*** | **<0.0001** | **<0.0001** | **<0.0001** | **<0.0001** | **<0.0001** |
|  |  |  | HH | 0.95 | 0.15 | 0.17 | 0.26 | 0.11 | **0.0001** |
|  |  |  | LL | **0.71*** | **<0.0001** | **<0.0001** | **<0.0001** | **<0.0001** | **<0.0001** |
| ShortRunEmphasis | texture | glrlm | LH | **0.83*** | **<0.0001** | **<0.0001** | **<0.0001** | **<0.0001** | **<0.0001** |
|  |  |  | HL | 0.91 | **<0.0001** | **<0.0001** | **<0.0001** | **<0.0001** | **<0.0001** |
|  |  |  | HH | 0.94 | 0.21 | **<0.0001** | **<0.0001** | **<0.0001** | **<0.0001** |
|  |  |  | LL | 0.93 | **<0.0001** | **<0.0001** | **<0.0001** | **<0.0001** | **<0.0001** |
| ShortRunHighGray  LevelEmphasis | texture | glrlm | LH | 0.93 | **<0.0001** | **<0.0001** | **<0.0001** | **<0.0001** | **<0.0001** |
|  |  |  | HL | 0.92 | **<0.0001** | **<0.0001** | **<0.0001** | **<0.0001** | **<0.0001** |
|  |  |  | HH | **0.80*** | **<0.0001** | **<0.0001** | **<0.0001** | **<0.0001** | **<0.0001** |
|  |  |  | LL | 0.99 | 0.56 | **0.04** | 0.09 | **0.02** | **0.003** |
| ShortRunLowGray  LevelEmphasis | texture | glrlm | LH | **0.83*** | **<0.0001** | **<0.0001** | **<0.0001** | **<0.0001** | **<0.0001** |
|  |  |  | HL | 0.87 | **<0.0001** | **<0.0001** | **<0.0001** | **<0.0001** | **<0.0001** |
|  |  |  | HH | **0.68*** | **<0.0001** | **<0.0001** | **<0.0001** | **<0.0001** | **<0.0001** |
|  |  |  | LL | 0.95 | 0.99 | 0.50 | 0.13 | 0.07 | 0.47 |
| GrayLevelNon  UniformityNormalized | texture | glszm | LH | **0.75*** | **<0.0001** | **<0.0001** | **<0.0001** | **<0.0001** | **<0.0001** |
|  |  |  | HL | **0.77*** | **<0.0001** | **<0.0001** | **<0.0001** | **<0.0001** | **<0.0001** |
|  |  |  | HH | **0.68*** | **<0.0001** | **<0.0001** | **<0.0001** | **<0.0001** | **<0.0001** |
|  |  |  | LL | 0.98 | **<0.0001** | **<0.0001** | **<0.0001** | **<0.0001** | **<0.0001** |
| GrayLevelVariance | texture | glszm | LH | **0.80*** | **<0.0001** | **<0.0001** | **<0.0001** | **<0.0001** | **<0.0001** |
|  |  |  | HL | **0.75*** | **<0.0001** | **<0.0001** | **<0.0001** | **<0.0001** | **<0.0001** |
|  |  |  | HH | **0.80*** | **<0.0001** | **<0.0001** | **<0.0001** | **<0.0001** | **<0.0001** |
|  |  |  | LL | 0.99 | **<0.0001** | **<0.0001** | **<0.0001** | **<0.0001** | **<0.0001** |
| HighGrayLevelZone  Emphasis | texture | glszm | LH | 0.89 | **<0.0001** | **<0.0001** | **<0.0001** | **<0.0001** | **<0.0001** |
|  |  |  | HL | 0.89 | **<0.0001** | **<0.0001** | **<0.0001** | **<0.0001** | **<0.0001** |
|  |  |  | HH | **0.78*** | **<0.0001** | **<0.0001** | **<0.0001** | **<0.0001** | **<0.0001** |
|  |  |  | LL | 0.99 | 0.23 | **0.04** | **0.0003** | **<0.0001** | **<0.0001** |
| LargeAreaEmphasis | texture | glszm | LH | **0.75*** | 0.19 | **0.001** | **<0.0001** | **<0.0001** | **<0.0001** |
|  |  |  | HL | 0.91 | **0.03** | **<0.0001** | **<0.0001** | **<0.0001** | **<0.0001** |
|  |  |  | HH | 0.97 | 0.52 | **0.0006** | **<0.0001** | **<0.0001** | **<0.0001** |
|  |  |  | LL | **0.66*** | 0.29 | **0.006** | **0.0001** | **<0.0001** | **<0.0001** |
| LargeAreaHighGray  LevelEmphasis | texture | glszm | LH | **0.71*** | 0.29 | **0.004** | **0.0001** | **<0.0001** | **<0.0001** |
|  |  |  | HL | **0.83*** | 0.06 | **<0.0001** | **<0.0001** | **<0.0001** | **<0.0001** |
|  |  |  | HH | 0.97 | **0.01** | 0.06 | **0.005** | **0.02** | 0.06 |
|  |  |  | LL | **0.65*** | 0.13 | **0.0001** | **<0.0001** | **<0.0001** | **<0.0001** |
| LargeAreaLowGray  LevelEmphasis | texture | glszm | LH | **0.82*** | 0.09 | **0.0001** | **<0.0001** | **<0.0001** | **<0.0001** |
|  |  |  | HL | 0.97 | **0.02** | **0.0002** | **0.0006** | **0.002** | 0.33 |
|  |  |  | HH | **0.84*** | 0.08 | **0.001** | **<0.0001** | **<0.0001** | **<0.0001** |
|  |  |  | LL | **0.67*** | 0.48 | 0.06 | **0.01** | **0.001** | **<0.0001** |
| LowGrayLevelZone  Emphasis | texture | glszm | LH | 0.89 | **<0.0001** | **<0.0001** | **<0.0001** | **<0.0001** | **<0.0001** |
|  |  |  | HL | 0.88 | **0.0054** | **<0.0001** | **<0.0001** | **<0.0001** | **<0.0001** |
|  |  |  | HH | **0.62*** | **<0.0001** | **<0.0001** | **<0.0001** | **<0.0001** | **<0.0001** |
|  |  |  | LL | 0.94 | 0.65 | 0.19 | 0.47 | 0.48 | **0.006** |
| SizeZoneNon  Uniformity | texture | glszm | LH | 0.97 | **0.01** | **<0.0001** | **<0.0001** | **<0.0001** | **<0.0001** |
|  |  |  | HL | 0.98 | 0.10 | 0.09 | 0.25 | 0.73 | **0.01** |
|  |  |  | HH | 0.91 | 0.33 | 0.38 | **0.01** | **<0.0001** | **<0.0001** |
|  |  |  | LL | 0.99 | **<0.0001** | **<0.0001** | **<0.0001** | **<0.0001** | **<0.0001** |
| SizeZoneNon  UniformityNormalized | texture | glszm | LH | **0.77*** | **<0.0001** | **<0.0001** | **<0.0001** | **<0.0001** | **<0.0001** |
|  |  |  | HL | **0.79*** | **<0.0001** | **<0.0001** | **<0.0001** | **<0.0001** | **<0.0001** |
|  |  |  | HH | **0.81*** | **<0.0001** | **<0.0001** | **<0.0001** | **<0.0001** | **0.0005** |
|  |  |  | LL | 0.99 | **0.0001** | **<0.0001** | **<0.0001** | **<0.0001** | **<0.0001** |
| SmallAreaEmphasis | texture | glszm | LH | **0.77*** | **<0.0001** | **<0.0001** | **<0.0001** | **<0.0001** | **<0.0001** |
|  |  |  | HL | **0.79*** | **<0.0001** | **<0.0001** | **<0.0001** | **<0.0001** | **<0.0001** |
|  |  |  | HH | **0.84*** | **<0.0001** | **<0.0001** | **<0.0001** | **<0.0001** | **0.003** |
|  |  |  | LL | 0.99 | **0.0007** | **<0.0001** | **<0.0001** | **<0.0001** | **<0.0001** |
| SmallAreaHighGray  LevelEmphasis | texture | glszm | LH | 0.87 | **<0.0001** | **<0.0001** | **<0.0001** | **<0.0001** | **<0.0001** |
|  |  |  | HL | 0.87 | **<0.0001** | **<0.0001** | **<0.0001** | **<0.0001** | **<0.0001** |
|  |  |  | HH | **0.79*** | **<0.0001** | **<0.0001** | **<0.0001** | **<0.0001** | **<0.0001** |
|  |  |  | LL | 0.98 | **0.03** | **<0.0001** | **<0.0001** | **<0.0001** | **<0.0001** |
| SmallAreaLowGray  LevelEmphasis | texture | glszm | LH | 0.91 | **0.001** | **0.0002** | **<0.0001** | **<0.0001** | **<0.0001** |
|  |  |  | HL | 0.89 | 0.25 | **0.0001** | **<0.0001** | **<0.0001** | **<0.0001** |
|  |  |  | HH | **0.60*** | **<0.0001** | **<0.0001** | **<0.0001** | **<0.0001** | **<0.0001** |
|  |  |  | LL | 0.94 | 0.77 | 0.10 | 0.18 | 0.18 | **0.0004** |
| ZoneEntropy | texture | glszm | LH | **0.65*** | **0.004** | **<0.0001** | **<0.0001** | **<0.0001** | **<0.0001** |
|  |  |  | HL | **0.58*** | **0.0001** | **<0.0001** | **<0.0001** | **<0.0001** | **<0.0001** |
|  |  |  | HH | **0.74*** | **<0.0001** | **<0.0001** | **<0.0001** | **<0.0001** | **<0.0001** |
|  |  |  | LL | 0.94 | **<0.0001** | **<0.0001** | **<0.0001** | **<0.0001** | **<0.0001** |
| ZonePercentage | texture | glszm | LH | 0.93 | **<0.0001** | **<0.0001** | **<0.0001** | **<0.0001** | **<0.0001** |
|  |  |  | HL | 0.98 | **0.0001** | **<0.0001** | **<0.0001** | **<0.0001** | **<0.0001** |
|  |  |  | HH | 0.92 | **0.03** | **<0.0001** | **<0.0001** | **<0.0001** | **<0.0001** |
|  |  |  | LL | 0.97 | **<0.0001** | **<0.0001** | **<0.0001** | **<0.0001** | **<0.0001** |
| ZoneVariance | texture | glszm | LH | **0.76*** | 0.19 | **0.001** | **<0.0001** | **<0.0001** | **<0.0001** |
|  |  |  | HL | 0.91 | **0.03** | **<0.0001** | **<0.0001** | **<0.0001** | **<0.0001** |
|  |  |  | HH | 0.97 | 0.49 | **0.0005** | **<0.0001** | **<0.0001** | **<0.0001** |
|  |  |  | LL | **0.65*** | 0.29 | **0.006** | **0.0001** | **<0.0001** | **<0.0001** |
| Coarseness_modified | texture | ngtdm1 | LH | 0.99 | **<0.0001** | **<0.0001** | **<0.0001** | **<0.0001** | **<0.0001** |
|  |  |  | HL | 0.99 | **<0.0001** | **<0.0001** | **<0.0001** | **<0.0001** | **0.0019** |
|  |  |  | HH | 0.94 | **<0.0001** | **<0.0001** | **<0.0001** | **<0.0001** | **<0.0001** |
|  |  |  | LL | 0.97 | **0.006** | **<0.0001** | **<0.0001** | **<0.0001** | **<0.0001** |
| Complexity | texture | ngtdm1 | LH | 0.93 | **0.0002** | **<0.0001** | **<0.0001** | **<0.0001** | **<0.0001** |
|  |  |  | HL | 0.91 | **0.0002** | **<0.0001** | **<0.0001** | **<0.0001** | **<0.0001** |
|  |  |  | HH | **0.81*** | **<0.0001** | **<0.0001** | **<0.0001** | **<0.0001** | **<0.0001** |
|  |  |  | LL | 0.99 | 0.65 | 0.60 | 0.13 | **0.02** | **<0.0001** |
| Contrast | texture | ngtdm1 | LH | 0.97 | **<0.0001** | **<0.0001** | **<0.0001** | **<0.0001** | **<0.0001** |
|  |  |  | HL | 0.99 | 0.69 | 0.20 | 0.49 | 0.44 | 0.22 |
|  |  |  | HH | **0.74*** | **<0.0001** | **<0.0001** | **<0.0001** | **<0.0001** | **<0.0001** |
|  |  |  | LL | 1.00 | 0.82 | 0.13 | 0.24 | 0.19 | 0.37 |
| Strength | texture | ngtdm1 | LH | 0.93 | **<0.0001** | **<0.0001** | **<0.0001** | **<0.0001** | **<0.0001** |
|  |  |  | HL | 0.93 | **<0.0001** | **<0.0001** | **<0.0001** | **<0.0001** | **<0.0001** |
|  |  |  | HH | 0.86 | **0.0004** | **<0.0001** | **<0.0001** | **<0.0001** | **<0.0001** |
|  |  |  | LL | 0.99 | **0.0002** | **<0.0001** | **<0.0001** | **<0.0001** | **<0.0001** |
| Coarseness_modified | texture | ngtdm4 | LH | 0.89 | **<0.0001** | **<0.0001** | **<0.0001** | **<0.0001** | **<0.0001** |
|  |  |  | HL | 0.95 | **<0.0001** | **<0.0001** | **<0.0001** | **<0.0001** | **<0.0001** |
|  |  |  | HH | 0.92 | **0.002** | **<0.0001** | **<0.0001** | **<0.0001** | **<0.0001** |
|  |  |  | LL | 1.00 | **0.0001** | **<0.0001** | **<0.0001** | **<0.0001** | **<0.0001** |
| Complexity | texture | ngtdm4 | LH | 0.94 | **<0.0001** | **<0.0001** | **<0.0001** | **<0.0001** | **<0.0001** |
|  |  |  | HL | 0.92 | **<0.0001** | **<0.0001** | **<0.0001** | **<0.0001** | **<0.0001** |
|  |  |  | HH | **0.81*** | **<0.0001** | **<0.0001** | **<0.0001** | **<0.0001** | **<0.0001** |
|  |  |  | LL | 0.99 | **0.03** | **0.0002** | **<0.0001** | **<0.0001** | **<0.0001** |
| Contrast | texture | ngtdm4 | LH | 0.98 | **<0.0001** | **<0.0001** | **<0.0001** | **<0.0001** | **<0.0001** |
|  |  |  | HL | 0.99 | 0.70 | 0.12 | 0.32 | 0.18 | **0.03** |
|  |  |  | HH | **0.76*** | **<0.0001** | **<0.0001** | **<0.0001** | **<0.0001** | **<0.0001** |
|  |  |  | LL | 1.00 | 0.61 | 0.97 | 0.32 | 0.15 | **0.0004** |
| Strength | texture | ngtdm4 | LH | 0.92 | **<0.0001** | **<0.0001** | **<0.0001** | **<0.0001** | **<0.0001** |
|  |  |  | HL | 0.92 | **<0.0001** | **<0.0001** | **<0.0001** | **<0.0001** | **<0.0001** |
|  |  |  | HH | 0.86 | **0.0004** | **<0.0001** | **<0.0001** | **<0.0001** | **<0.0001** |
|  |  |  | LL | 0.99 | **0.0004** | **<0.0001** | **<0.0001** | **<0.0001** | **<0.0001** |
| Coarseness_modified | texture | ngtdm7 | LH | 0.91 | **<0.0001** | **<0.0001** | **<0.0001** | **<0.0001** | **<0.0001** |
|  |  |  | HL | 0.96 | **<0.0001** | **<0.0001** | **<0.0001** | **<0.0001** | **<0.0001** |
|  |  |  | HH | 0.92 | **0.001** | **<0.0001** | **<0.0001** | **<0.0001** | **<0.0001** |
|  |  |  | LL | 1.00 | **<0.0001** | **<0.0001** | **<0.0001** | **<0.0001** | **<0.0001** |
| Complexity | texture | ngtdm7 | LH | 0.94 | **<0.0001** | **<0.0001** | **<0.0001** | **<0.0001** | **<0.0001** |
|  |  |  | HL | 0.92 | **<0.0001** | **<0.0001** | **<0.0001** | **<0.0001** | **<0.0001** |
|  |  |  | HH | **0.81*** | **<0.0001** | **<0.0001** | **<0.0001** | **<0.0001** | **<0.0001** |
|  |  |  | LL | 1.00 | **0.009** | **<0.0001** | **<0.0001** | **<0.0001** | **<0.0001** |
| Contrast | texture | ngtdm7 | LH | 0.98 | **<0.0001** | **<0.0001** | **<0.0001** | **<0.0001** | **<0.0001** |
|  |  |  | HL | 0.99 | 0.74 | 0.15 | 0.39 | 0.25 | 0.06 |
|  |  |  | HH | **0.76*** | **<0.0001** | **<0.0001** | **<0.0001** | **<0.0001** | **<0.0001** |
|  |  |  | LL | 1.00 | 0.41 | 0.46 | 0.07 | **0.01** | **<0.0001** |
| Strength | texture | ngtdm7 | LH | 0.92 | **<0.0001** | **<0.0001** | **<0.0001** | **<0.0001** | **<0.0001** |
|  |  |  | HL | 0.92 | **<0.0001** | **<0.0001** | **<0.0001** | **<0.0001** | **<0.0001** |
|  |  |  | HH | 0.86 | **0.0004** | **<0.0001** | **<0.0001** | **<0.0001** | **<0.0001** |
|  |  |  | LL | 0.99 | **0.0007** | **<0.0001** | **<0.0001** | **<0.0001** | **<0.0001** |

Notes: Significant FDR-adjusted p values for the multivariate model are in bold. OCCC < 0.85 are in bold and marked with an asterisk. Reference algorithm for mixed model analysis=FBP.

**Table S5. OCCC values and False Discovery Rate (FDR) adjusted p value from the multivariable mixed model for the reconstruction algorithm impact (FDR corrected) for all the LoG features.**

| **Feature** | **Category** | **Sub-category** | **Sigma**  **(mm)** | **OCCC** | **IR20**  **(mixed)** | **IR40 (mixed)** | **IR50 (mixed)** | **IR60 (mixed)** | **IR80 (mixed)** |
| --- | --- | --- | --- | --- | --- | --- | --- | --- | --- |
| 10Percentile | firstorder |  | 0.5 | 0.98 | 0.31 | **0.007** | **0.0002** | **<0.0001** | **<0.0001** |
|  |  |  | 1 | 1.00 | **<0.0001** | **<0.0001** | **<0.0001** | **<0.0001** | **<0.0001** |
|  |  |  | 1.5 | 1.00 | **<0.0001** | **<0.0001** | **<0.0001** | **<0.0001** | **<0.0001** |
|  |  |  | 2.5 | 1.00 | **<0.0001** | **<0.0001** | **<0.0001** | **<0.0001** | **<0.0001** |
|  |  |  | 5 | 1.00 | **<0.0001** | **<0.0001** | **<0.0001** | **<0.0001** | **<0.0001** |
| 90Percentile | firstorder |  | 0.5 | 0.97 | **0.0009** | **<0.0001** | **<0.0001** | **<0.0001** | **<0.0001** |
|  |  |  | 1 | 1.00 | 0.35 | 0.06 | **0.02** | **0.007** | **0.0006** |
|  |  |  | 1.5 | 1.00 | 0.94 | 0.83 | 0.74 | 0.54 | 0.47 |
|  |  |  | 2.5 | 1.00 | 0.18 | **0.009** | **0.0006** | **<0.0001** | **<0.0001** |
|  |  |  | 5 | 1.00 | 0.62 | 0.06 | **0.03** | **0.005** | **0.01** |
| Entropy | firstorder |  | 0.5 | 0.99 | 0.16 | **0.04** | **0.048** | 0.12 | 0.82 |
|  |  |  | 1 | 1.00 | 0.32 | **0.02** | **0.002** | **0.0001** | **<0.0001** |
|  |  |  | 1.5 | 1.00 | **0.001** | **<0.0001** | **<0.0001** | **<0.0001** | **<0.0001** |
|  |  |  | 2.5 | 1.00 | **<0.0001** | **<0.0001** | **<0.0001** | **<0.0001** | **<0.0001** |
|  |  |  | 5 | 1.00 | **<0.0001** | **<0.0001** | **<0.0001** | **<0.0001** | **<0.0001** |
| InterquartileRange | firstorder |  | 0.5 | 0.95 | **<0.0001** | **<0.0001** | **<0.0001** | **<0.0001** | **<0.0001** |
|  |  |  | 1 | 1.00 | 0.59 | 0.18 | 0.13 | 0.06 | **0.02** |
|  |  |  | 1.5 | 1.00 | **0.005** | **<0.0001** | **<0.0001** | **<0.0001** | **<0.0001** |
|  |  |  | 2.5 | 1.00 | **<0.0001** | **<0.0001** | **<0.0001** | **<0.0001** | **<0.0001** |
|  |  |  | 5 | 1.00 | **<0.0001** | **<0.0001** | **<0.0001** | **<0.0001** | **<0.0001** |
| Kurtosis | firstorder |  | 0.5 | **0.75*** | 0.16 | **0.001** | **<0.0001** | **<0.0001** | **<0.0001** |
|  |  |  | 1 | 0.99 | **0.008** | **<0.0001** | **<0.0001** | **<0.0001** | **<0.0001** |
|  |  |  | 1.5 | 1.00 | **0.0001** | **<0.0001** | **<0.0001** | **<0.0001** | **<0.0001** |
|  |  |  | 2.5 | 1.00 | **0.0006** | **<0.0001** | **<0.0001** | **<0.0001** | **<0.0001** |
|  |  |  | 5 | 1.00 | **0.02** | **<0.0001** | **<0.0001** | **<0.0001** | **<0.0001** |
| Maximum | firstorder |  | 0.5 | 0.93 | **<0.0001** | **<0.0001** | **<0.0001** | **<0.0001** | **<0.0001** |
|  |  |  | 1 | 0.98 | **<0.0001** | **<0.0001** | **<0.0001** | **<0.0001** | **<0.0001** |
|  |  |  | 1.5 | 1.00 | **<0.0001** | **<0.0001** | **<0.0001** | **<0.0001** | **<0.0001** |
|  |  |  | 2.5 | 1.00 | **<0.0001** | **<0.0001** | **<0.0001** | **<0.0001** | **<0.0001** |
|  |  |  | 5 | 1.00 | **<0.0001** | **<0.0001** | **<0.0001** | **<0.0001** | **<0.0001** |
| Mean | firstorder |  | 0.5 | 1.00 | **<0.0001** | **<0.0001** | **<0.0001** | **<0.0001** | **<0.0001** |
|  |  |  | 1 | 1.00 | **<0.0001** | **<0.0001** | **<0.0001** | **<0.0001** | **<0.0001** |
|  |  |  | 1.5 | 1.00 | **<0.0001** | **<0.0001** | **<0.0001** | **<0.0001** | **<0.0001** |
|  |  |  | 2.5 | 1.00 | **<0.0001** | **<0.0001** | **<0.0001** | **<0.0001** | **<0.0001** |
|  |  |  | 5 | 1.00 | **<0.0001** | **<0.0001** | **<0.0001** | **<0.0001** | **<0.0001** |
| MeanAbsolute  Deviation | firstorder |  | 0.5 | 0.99 | 0.51 | 0.33 | 0.38 | 0.54 | 0.60 |
|  |  |  | 1 | 1.00 | **<0.0001** | **<0.0001** | **<0.0001** | **<0.0001** | **<0.0001** |
|  |  |  | 1.5 | 1.00 | **<0.0001** | **<0.0001** | **<0.0001** | **<0.0001** | **<0.0001** |
|  |  |  | 2.5 | 1.00 | **<0.0001** | **<0.0001** | **<0.0001** | **<0.0001** | **<0.0001** |
|  |  |  | 5 | 1.00 | **<0.0001** | **<0.0001** | **<0.0001** | **<0.0001** | **<0.0001** |
| Median | firstorder |  | 0.5 | 0.96 | **<0.0001** | **<0.0001** | **<0.0001** | **<0.0001** | **<0.0001** |
|  |  |  | 1 | 1.00 | **<0.0001** | **<0.0001** | **<0.0001** | **<0.0001** | **<0.0001** |
|  |  |  | 1.5 | 1.00 | 0.59 | 0.18 | 0.08 | 0.06 | **0.01** |
|  |  |  | 2.5 | 1.00 | **0.03** | **<0.0001** | **<0.0001** | **<0.0001** | **<0.0001** |
|  |  |  | 5 | 1.00 | **<0.0001** | **<0.0001** | **<0.0001** | **<0.0001** | **<0.0001** |
| Minimum | firstorder |  | 0.5 | 0.88 | **<0.0001** | **<0.0001** | **<0.0001** | **<0.0001** | **<0.0001** |
|  |  |  | 1 | 0.97 | **<0.0001** | **<0.0001** | **<0.0001** | **<0.0001** | **<0.0001** |
|  |  |  | 1.5 | 0.99 | **<0.0001** | **<0.0001** | **<0.0001** | **<0.0001** | **<0.0001** |
|  |  |  | 2.5 | 1.00 | **<0.0001** | **<0.0001** | **<0.0001** | **<0.0001** | **<0.0001** |
|  |  |  | 5 | 1.00 | **<0.0001** | **<0.0001** | **<0.0001** | **<0.0001** | **<0.0001** |
| Range | firstorder |  | 0.5 | 0.89 | **<0.0001** | **<0.0001** | **<0.0001** | **<0.0001** | **<0.0001** |
|  |  |  | 1 | 0.97 | **<0.0001** | **<0.0001** | **<0.0001** | **<0.0001** | **<0.0001** |
|  |  |  | 1.5 | 0.99 | **<0.0001** | **<0.0001** | **<0.0001** | **<0.0001** | **<0.0001** |
|  |  |  | 2.5 | 1.00 | **<0.0001** | **<0.0001** | **<0.0001** | **<0.0001** | **<0.0001** |
|  |  |  | 5 | 1.00 | **<0.0001** | **<0.0001** | **<0.0001** | **<0.0001** | **<0.0001** |
| RobustMeanAbsoluteDeviation | firstorder |  | 0.5 | 0.97 | **<0.0001** | **<0.0001** | **<0.0001** | **<0.0001** | **<0.0001** |
|  |  |  | 1 | 1.00 | 0.20 | **0.004** | **0.0003** | **<0.0001** | **<0.0001** |
|  |  |  | 1.5 | 1.00 | **<0.0001** | **<0.0001** | **<0.0001** | **<0.0001** | **<0.0001** |
|  |  |  | 2.5 | 1.00 | **<0.0001** | **<0.0001** | **<0.0001** | **<0.0001** | **<0.0001** |
|  |  |  | 5 | 1.00 | **<0.0001** | **<0.0001** | **<0.0001** | **<0.0001** | **<0.0001** |
| RootMeanSquared | firstorder |  | 0.5 | 1.00 | **<0.0001** | **<0.0001** | **<0.0001** | **<0.0001** | **<0.0001** |
|  |  |  | 1 | 1.00 | **<0.0001** | **<0.0001** | **<0.0001** | **<0.0001** | **<0.0001** |
|  |  |  | 1.5 | 1.00 | **<0.0001** | **<0.0001** | **<0.0001** | **<0.0001** | **<0.0001** |
|  |  |  | 2.5 | 1.00 | **<0.0001** | **<0.0001** | **<0.0001** | **<0.0001** | **<0.0001** |
|  |  |  | 5 | 1.00 | **<0.0001** | **<0.0001** | **<0.0001** | **<0.0001** | **<0.0001** |
| Skewness | firstorder |  | 0.5 | 0.93 | 0.55 | 0.11 | **0.03** | **0.005** | **<0.0001** |
|  |  |  | 1 | 1.00 | 0.93 | 0.72 | 0.59 | 0.45 | 0.20 |
|  |  |  | 1.5 | 1.00 | 0.98 | 0.89 | 0.80 | 0.69 | 0.47 |
|  |  |  | 2.5 | 1.00 | 0.08 | **0.0002** | **<0.0001** | **<0.0001** | **<0.0001** |
|  |  |  | 5 | 1.00 | 0.94 | 0.84 | 0.85 | 0.81 | 0.73 |
| TotalEnergy_modified | firstorder |  | 0.5 | 1.00 | **<0.0001** | **<0.0001** | **<0.0001** | **<0.0001** | **<0.0001** |
|  |  |  | 1 | 1.00 | **<0.0001** | **<0.0001** | **<0.0001** | **<0.0001** | **<0.0001** |
|  |  |  | 1.5 | 1.00 | **<0.0001** | **<0.0001** | **<0.0001** | **<0.0001** | **<0.0001** |
|  |  |  | 2.5 | 1.00 | **<0.0001** | **<0.0001** | **<0.0001** | **<0.0001** | **<0.0001** |
|  |  |  | 5 | 1.00 | **<0.0001** | **<0.0001** | **<0.0001** | **<0.0001** | **<0.0001** |
| Uniformity | firstorder |  | 0.5 | 0.98 | **<0.0001** | **<0.0001** | **<0.0001** | **<0.0001** | **<0.0001** |
|  |  |  | 1 | 1.00 | **0.0001** | **<0.0001** | **<0.0001** | **<0.0001** | **<0.0001** |
|  |  |  | 1.5 | 1.00 | 0.06 | **0.0003** | **<0.0001** | **<0.0001** | **<0.0001** |
|  |  |  | 2.5 | 1.00 | 0.14 | **0.002** | **0.0001** | **<0.0001** | **<0.0001** |
|  |  |  | 5 | 1.00 | **<0.0001** | **<0.0001** | **<0.0001** | **<0.0001** | **<0.0001** |
| Variance | firstorder |  | 0.5 | 0.97 | **0.0003** | **<0.0001** | **<0.0001** | **<0.0001** | **<0.0001** |
|  |  |  | 1 | 0.99 | **<0.0001** | **<0.0001** | **<0.0001** | **<0.0001** | **<0.0001** |
|  |  |  | 1.5 | 1.00 | **<0.0001** | **<0.0001** | **<0.0001** | **<0.0001** | **<0.0001** |
|  |  |  | 2.5 | 1.00 | **<0.0001** | **<0.0001** | **<0.0001** | **<0.0001** | **<0.0001** |
|  |  |  | 5 | 1.00 | **<0.0001** | **<0.0001** | **<0.0001** | **<0.0001** | **<0.0001** |
| Autocorrelation | texture | glcm1 | 0.5 | 0.86 | **<0.0001** | **<0.0001** | **<0.0001** | **<0.0001** | **<0.0001** |
|  |  |  | 1 | 0.96 | **<0.0001** | **<0.0001** | **<0.0001** | **<0.0001** | **<0.0001** |
|  |  |  | 1.5 | 0.99 | 0.09 | **<0.0001** | **<0.0001** | **<0.0001** | **<0.0001** |
|  |  |  | 2.5 | 0.99 | 0.37 | **0.04** | **0.005** | **<0.0001** | **<0.0001** |
|  |  |  | 5 | 1.00 | 0.73 | 0.18 | **0.007** | **0.009** | **0.01** |
| ClusterProminence | texture | glcm1 | 0.5 | 0.93 | **0.002** | **<0.0001** | **<0.0001** | **<0.0001** | **<0.0001** |
|  |  |  | 1 | 0.98 | **0.0005** | **<0.0001** | **<0.0001** | **<0.0001** | **<0.0001** |
|  |  |  | 1.5 | 0.99 | **0.0003** | **<0.0001** | **<0.0001** | **<0.0001** | **<0.0001** |
|  |  |  | 2.5 | 1.00 | 0.06 | **<0.0001** | **<0.0001** | **<0.0001** | **<0.0001** |
|  |  |  | 5 | 1.00 | **0.0003** | **<0.0001** | **<0.0001** | **<0.0001** | **<0.0001** |
| ClusterShade | texture | glcm1 | 0.5 | 0.98 | 0.38 | **0.03** | **0.007** | **0.002** | **<0.0001** |
|  |  |  | 1 | 0.99 | 0.33 | **0.03** | **0.006** | **0.001** | **<0.0001** |
|  |  |  | 1.5 | 1.00 | 0.49 | 0.13 | 0.06 | **0.02** | **0.002** |
|  |  |  | 2.5 | 1.00 | 0.76 | 0.37 | 0.30 | 0.20 | 0.067 |
|  |  |  | 5 | 1.00 | 0.10 | **0.002** | **0.0001** | **<0.0001** | **<0.0001** |
| ClusterTendency | texture | glcm1 | 0.5 | 0.97 | **<0.0001** | **<0.0001** | **<0.0001** | **<0.0001** | **<0.0001** |
|  |  |  | 1 | 0.99 | **<0.0001** | **<0.0001** | **<0.0001** | **<0.0001** | **<0.0001** |
|  |  |  | 1.5 | 1.00 | **<0.0001** | **<0.0001** | **<0.0001** | **<0.0001** | **<0.0001** |
|  |  |  | 2.5 | 1.00 | **<0.0001** | **<0.0001** | **<0.0001** | **<0.0001** | **<0.0001** |
|  |  |  | 5 | 1.00 | **<0.0001** | **<0.0001** | **<0.0001** | **<0.0001** | **<0.0001** |
| Contrast | texture | glcm1 | 0.5 | 0.95 | **0.008** | **<0.0001** | **<0.0001** | **<0.0001** | **<0.0001** |
|  |  |  | 1 | 0.98 | **<0.0001** | **<0.0001** | **<0.0001** | **<0.0001** | **<0.0001** |
|  |  |  | 1.5 | 1.00 | **<0.0001** | **<0.0001** | **<0.0001** | **<0.0001** | **<0.0001** |
|  |  |  | 2.5 | 1.00 | **<0.0001** | **<0.0001** | **<0.0001** | **<0.0001** | **<0.0001** |
|  |  |  | 5 | 1.00 | **<0.0001** | **<0.0001** | **<0.0001** | **<0.0001** | **<0.0001** |
| Correlation | texture | glcm1 | 0.5 | 0.91 | 0.23 | 0.31 | 0.74 | 0.63 | **0.03** |
|  |  |  | 1 | 0.98 | **<0.0001** | **<0.0001** | **<0.0001** | **<0.0001** | **<0.0001** |
|  |  |  | 1.5 | 1.00 | **<0.0001** | **<0.0001** | **<0.0001** | **<0.0001** | **<0.0001** |
|  |  |  | 2.5 | 1.00 | **<0.0001** | **<0.0001** | **<0.0001** | **<0.0001** | **<0.0001** |
|  |  |  | 5 | 1.00 | **0.0006** | **<0.0001** | **<0.0001** | **<0.0001** | **<0.0001** |
| DifferenceAverage | texture | glcm1 | 0.5 | 0.98 | 0.15 | **0.047** | 0.051 | 0.08 | 0.16 |
|  |  |  | 1 | 1.00 | 0.15 | **0.0009** | **<0.0001** | **<0.0001** | **<0.0001** |
|  |  |  | 1.5 | 1.00 | **0.0005** | **<0.0001** | **<0.0001** | **<0.0001** | **<0.0001** |
|  |  |  | 2.5 | 1.00 | **<0.0001** | **<0.0001** | **<0.0001** | **<0.0001** | **<0.0001** |
|  |  |  | 5 | 1.00 | **<0.0001** | **<0.0001** | **<0.0001** | **<0.0001** | **<0.0001** |
| DifferenceEntropy | texture | glcm1 | 0.5 | 0.98 | 0.96 | 0.23 | **0.02** | **0.0002** | **<0.0001** |
|  |  |  | 1 | 1.00 | **0.01** | **<0.0001** | **<0.0001** | **<0.0001** | **<0.0001** |
|  |  |  | 1.5 | 1.00 | **<0.0001** | **<0.0001** | **<0.0001** | **<0.0001** | **<0.0001** |
|  |  |  | 2.5 | 1.00 | **<0.0001** | **<0.0001** | **<0.0001** | **<0.0001** | **<0.0001** |
|  |  |  | 5 | 1.00 | **<0.0001** | **<0.0001** | **<0.0001** | **<0.0001** | **<0.0001** |
| DifferenceVariance | texture | glcm1 | 0.5 | 0.92 | **<0.0001** | **<0.0001** | **<0.0001** | **<0.0001** | **<0.0001** |
|  |  |  | 1 | 0.97 | **<0.0001** | **<0.0001** | **<0.0001** | **<0.0001** | **<0.0001** |
|  |  |  | 1.5 | 0.99 | **<0.0001** | **<0.0001** | **<0.0001** | **<0.0001** | **<0.0001** |
|  |  |  | 2.5 | 1.00 | **<0.0001** | **<0.0001** | **<0.0001** | **<0.0001** | **<0.0001** |
|  |  |  | 5 | 1.00 | **<0.0001** | **<0.0001** | **<0.0001** | **<0.0001** | **<0.0001** |
| Id | texture | glcm1 | 0.5 | 0.92 | **<0.0001** | **<0.0001** | **<0.0001** | **<0.0001** | **<0.0001** |
|  |  |  | 1 | 0.99 | **<0.0001** | **<0.0001** | **<0.0001** | **<0.0001** | **<0.0001** |
|  |  |  | 1.5 | 1.00 | 0.31 | **0.04** | **0.006** | **0.0008** | **<0.0001** |
|  |  |  | 2.5 | 1.00 | **0.001** | **<0.0001** | **<0.0001** | **<0.0001** | **<0.0001** |
|  |  |  | 5 | 1.00 | **<0.0001** | **<0.0001** | **<0.0001** | **<0.0001** | **<0.0001** |
| Idm | texture | glcm1 | 0.5 | 0.94 | **<0.0001** | **<0.0001** | **<0.0001** | **<0.0001** | **<0.0001** |
|  |  |  | 1 | 1.00 | **<0.0001** | **<0.0001** | **<0.0001** | **<0.0001** | **<0.0001** |
|  |  |  | 1.5 | 1.00 | 0.70 | 0.46 | 0.28 | 0.18 | 0.09 |
|  |  |  | 2.5 | 1.00 | **0.0002** | **<0.0001** | **<0.0001** | **<0.0001** | **<0.0001** |
|  |  |  | 5 | 1.00 | **<0.0001** | **<0.0001** | **<0.0001** | **<0.0001** | **<0.0001** |
| Idmn | texture | glcm1 | 0.5 | 0.97 | **<0.0001** | **<0.0001** | **<0.0001** | **<0.0001** | **<0.0001** |
|  |  |  | 1 | 1.00 | **0.04** | **0.01** | **0.005** | **0.0004** | **<0.0001** |
|  |  |  | 1.5 | 1.00 | 0.75 | 0.95 | 0.94 | 0.85 | 0.33 |
|  |  |  | 2.5 | 1.00 | 0.88 | 0.69 | 0.27 | 0.15 | 0.17 |
|  |  |  | 5 | 1.00 | 0.92 | 0.93 | 0.33 | 0.56 | 0.65 |
| Idn | texture | glcm1 | 0.5 | 0.91 | **<0.0001** | **<0.0001** | **<0.0001** | **<0.0001** | **<0.0001** |
|  |  |  | 1 | 0.99 | **<0.0001** | **<0.0001** | **<0.0001** | **<0.0001** | **<0.0001** |
|  |  |  | 1.5 | 1.00 | 0.10 | **<0.0001** | **<0.0001** | **<0.0001** | **<0.0001** |
|  |  |  | 2.5 | 1.00 | 0.71 | 0.71 | 0.95 | 0.95 | 0.61 |
|  |  |  | 5 | 1.00 | 0.94 | 0.98 | 0.44 | 0.76 | 0.37 |
| Imc1 | texture | glcm1 | 0.5 | **0.74*** | **<0.0001** | **<0.0001** | **<0.0001** | **<0.0001** | **<0.0001** |
|  |  |  | 1 | 0.97 | **<0.0001** | **<0.0001** | **<0.0001** | **<0.0001** | **<0.0001** |
|  |  |  | 1.5 | 1.00 | **0.04** | **<0.0001** | **<0.0001** | **<0.0001** | **<0.0001** |
|  |  |  | 2.5 | 1.00 | **0.0005** | **<0.0001** | **<0.0001** | **<0.0001** | **<0.0001** |
|  |  |  | 5 | 1.00 | **<0.0001** | **<0.0001** | **<0.0001** | **<0.0001** | **<0.0001** |
| Imc2 | texture | glcm1 | 0.5 | 0.90 | **<0.0001** | **<0.0001** | **<0.0001** | **<0.0001** | **<0.0001** |
|  |  |  | 1 | 0.97 | **<0.0001** | **<0.0001** | **<0.0001** | **<0.0001** | **<0.0001** |
|  |  |  | 1.5 | 1.00 | **0.0003** | **<0.0001** | **<0.0001** | **<0.0001** | **<0.0001** |
|  |  |  | 2.5 | 1.00 | 0.38 | **0.002** | **<0.0001** | **<0.0001** | **<0.0001** |
|  |  |  | 5 | 1.00 | **0.03** | **<0.0001** | **<0.0001** | **<0.0001** | **<0.0001** |
| InverseVariance | texture | glcm1 | 0.5 | **0.32*** | **<0.0001** | **<0.0001** | **<0.0001** | **<0.0001** | **<0.0001** |
|  |  |  | 1 | **0.81*** | **<0.0001** | **<0.0001** | **<0.0001** | **<0.0001** | **<0.0001** |
|  |  |  | 1.5 | 0.99 | **<0.0001** | **<0.0001** | **<0.0001** | **<0.0001** | **<0.0001** |
|  |  |  | 2.5 | 1.00 | **<0.0001** | **<0.0001** | **<0.0001** | **<0.0001** | **<0.0001** |
|  |  |  | 5 | 1.00 | 0.76 | 0.46 | 0.28 | 0.12 | 0.48 |
| JointEnergy | texture | glcm1 | 0.5 | 0.94 | **<0.0001** | **<0.0001** | **<0.0001** | **<0.0001** | **<0.0001** |
|  |  |  | 1 | 0.99 | **<0.0001** | **<0.0001** | **<0.0001** | **<0.0001** | **<0.0001** |
|  |  |  | 1.5 | 1.00 | **<0.0001** | **<0.0001** | **<0.0001** | **<0.0001** | **<0.0001** |
|  |  |  | 2.5 | 1.00 | 0.11 | **0.0009** | **<0.0001** | **<0.0001** | **<0.0001** |
|  |  |  | 5 | 1.00 | **<0.0001** | **<0.0001** | **<0.0001** | **<0.0001** | **<0.0001** |
| JointEntropy | texture | glcm1 | 0.5 | 0.99 | **0.0009** | **<0.0001** | **<0.0001** | **<0.0001** | **<0.0001** |
|  |  |  | 1 | 1.00 | 0.21 | **0.01** | **0.002** | **0.0005** | **<0.0001** |
|  |  |  | 1.5 | 1.00 | 0.15 | **0.002** | **0.0002** | **<0.0001** | **<0.0001** |
|  |  |  | 2.5 | 1.00 | **<0.0001** | **<0.0001** | **<0.0001** | **<0.0001** | **<0.0001** |
|  |  |  | 5 | 1.00 | **<0.0001** | **<0.0001** | **<0.0001** | **<0.0001** | **<0.0001** |
| MaximumProbability | texture | glcm1 | 0.5 | **0.76*** | **<0.0001** | **<0.0001** | **<0.0001** | **<0.0001** | **<0.0001** |
|  |  |  | 1 | 0.98 | **<0.0001** | **<0.0001** | **<0.0001** | **<0.0001** | **<0.0001** |
|  |  |  | 1.5 | 1.00 | **<0.0001** | **<0.0001** | **<0.0001** | **<0.0001** | **<0.0001** |
|  |  |  | 2.5 | 1.00 | **0.001** | **<0.0001** | **<0.0001** | **<0.0001** | **<0.0001** |
|  |  |  | 5 | 1.00 | 0.25 | 0.06 | **0.01** | **0.003** | **0.02** |
| MCC | texture | glcm1 | 0.5 | **0.81*** | **<0.0001** | **<0.0001** | **<0.0001** | **<0.0001** | **<0.0001** |
|  |  |  | 1 | 0.98 | 0.09 | **<0.0001** | **<0.0001** | **<0.0001** | **<0.0001** |
|  |  |  | 1.5 | 0.99 | 0.44 | 0.10 | **0.03** | 0.20 | **0.02** |
|  |  |  | 2.5 | 1.00 | **0.03** | **0.003** | **0.001** | **0.0002** | **<0.0001** |
|  |  |  | 5 | 1.00 | 0.17 | 0.18 | 0.21 | 0.24 | 0.31 |
| SumAverage | texture | glcm1 | 0.5 | 0.85 | **<0.0001** | **<0.0001** | **<0.0001** | **<0.0001** | **<0.0001** |
|  |  |  | 1 | 0.96 | **<0.0001** | **<0.0001** | **<0.0001** | **<0.0001** | **<0.0001** |
|  |  |  | 1.5 | 0.98 | 0.09 | **<0.0001** | **<0.0001** | **<0.0001** | **<0.0001** |
|  |  |  | 2.5 | 0.99 | 0.29 | **0.02** | **0.004** | **<0.0001** | **<0.0001** |
|  |  |  | 5 | 1.00 | 0.74 | 0.16 | **0.01** | **0.01** | **0.02** |
| SumEntropy | texture | glcm1 | 0.5 | 0.99 | **0.02** | **0.0005** | **0.0003** | **0.0007** | **0.02** |
|  |  |  | 1 | 1.00 | 0.18 | **0.005** | **0.0004** | **<0.0001** | **<0.0001** |
|  |  |  | 1.5 | 1.00 | 0.82 | 0.55 | 0.46 | 0.35 | 0.19 |
|  |  |  | 2.5 | 1.00 | **0.0001** | **<0.0001** | **<0.0001** | **<0.0001** | **<0.0001** |
|  |  |  | 5 | 1.00 | **<0.0001** | **<0.0001** | **<0.0001** | **<0.0001** | **<0.0001** |
| SumSquares | texture | glcm1 | 0.5 | 0.97 | **0.0004** | **<0.0001** | **<0.0001** | **<0.0001** | **<0.0001** |
|  |  |  | 1 | 0.99 | **<0.0001** | **<0.0001** | **<0.0001** | **<0.0001** | **<0.0001** |
|  |  |  | 1.5 | 1.00 | **<0.0001** | **<0.0001** | **<0.0001** | **<0.0001** | **<0.0001** |
|  |  |  | 2.5 | 1.00 | **<0.0001** | **<0.0001** | **<0.0001** | **<0.0001** | **<0.0001** |
|  |  |  | 5 | 1.00 | **<0.0001** | **<0.0001** | **<0.0001** | **<0.0001** | **<0.0001** |
| Autocorrelation | texture | glcm4 | 0.5 | 0.85 | **<0.0001** | **<0.0001** | **<0.0001** | **<0.0001** | **<0.0001** |
|  |  |  | 1 | 0.95 | **<0.0001** | **<0.0001** | **<0.0001** | **<0.0001** | **<0.0001** |
|  |  |  | 1.5 | 0.98 | 0.09 | **<0.0001** | **<0.0001** | **<0.0001** | **<0.0001** |
|  |  |  | 2.5 | 0.99 | 0.38 | **0.04** | **0.006** | **<0.0001** | **<0.0001** |
|  |  |  | 5 | 1.00 | 0.73 | 0.18 | **0.009** | **0.01** | **0.01** |
| ClusterProminence | texture | glcm4 | 0.5 | 0.92 | **0.005** | **<0.0001** | **<0.0001** | **<0.0001** | **<0.0001** |
|  |  |  | 1 | 0.98 | **0.0004** | **<0.0001** | **<0.0001** | **<0.0001** | **<0.0001** |
|  |  |  | 1.5 | 1.00 | **0.001** | **<0.0001** | **<0.0001** | **<0.0001** | **<0.0001** |
|  |  |  | 2.5 | 1.00 | 0.13 | **0.0007** | **<0.0001** | **<0.0001** | **<0.0001** |
|  |  |  | 5 | 1.00 | **0.001** | **<0.0001** | **<0.0001** | **<0.0001** | **<0.0001** |
| ClusterShade | texture | glcm4 | 0.5 | 0.97 | 0.55 | 0.13 | **0.049** | **0.02** | **0.001** |
|  |  |  | 1 | 0.99 | 0.98 | 0.95 | 0.97 | 0.99 | 0.95 |
|  |  |  | 1.5 | 1.00 | 0.85 | 0.66 | 0.52 | 0.42 | 0.27 |
|  |  |  | 2.5 | 1.00 | 0.84 | 0.54 | 0.51 | 0.39 | 0.20 |
|  |  |  | 5 | 1.00 | 0.08 | **0.0008** | **<0.0001** | **<0.0001** | **<0.0001** |
| ClusterTendency | texture | glcm4 | 0.5 | 0.97 | **0.001** | **<0.0001** | **<0.0001** | **<0.0001** | **<0.0001** |
|  |  |  | 1 | 0.99 | **<0.0001** | **<0.0001** | **<0.0001** | **<0.0001** | **<0.0001** |
|  |  |  | 1.5 | 1.00 | **<0.0001** | **<0.0001** | **<0.0001** | **<0.0001** | **<0.0001** |
|  |  |  | 2.5 | 1.00 | **<0.0001** | **<0.0001** | **<0.0001** | **<0.0001** | **<0.0001** |
|  |  |  | 5 | 1.00 | **<0.0001** | **<0.0001** | **<0.0001** | **<0.0001** | **<0.0001** |
| Contrast | texture | glcm4 | 0.5 | 0.97 | **0.002** | **<0.0001** | **<0.0001** | **<0.0001** | **<0.0001** |
|  |  |  | 1 | 0.99 | **<0.0001** | **<0.0001** | **<0.0001** | **<0.0001** | **<0.0001** |
|  |  |  | 1.5 | 1.00 | **<0.0001** | **<0.0001** | **<0.0001** | **<0.0001** | **<0.0001** |
|  |  |  | 2.5 | 1.00 | **<0.0001** | **<0.0001** | **<0.0001** | **<0.0001** | **<0.0001** |
|  |  |  | 5 | 1.00 | **<0.0001** | **<0.0001** | **<0.0001** | **<0.0001** | **<0.0001** |
| Correlation | texture | glcm4 | 0.5 | 0.99 | **0.001** | **<0.0001** | **<0.0001** | **<0.0001** | **<0.0001** |
|  |  |  | 1 | 1.00 | **0.002** | **<0.0001** | **<0.0001** | **<0.0001** | **<0.0001** |
|  |  |  | 1.5 | 1.00 | **<0.0001** | **<0.0001** | **<0.0001** | **<0.0001** | **<0.0001** |
|  |  |  | 2.5 | 1.00 | **<0.0001** | **<0.0001** | **<0.0001** | **<0.0001** | **<0.0001** |
|  |  |  | 5 | 1.00 | **<0.0001** | **<0.0001** | **<0.0001** | **<0.0001** | **<0.0001** |
| DifferenceAverage | texture | glcm4 | 0.5 | 0.99 | 0.85 | 0.40 | 0.06 | **0.001** | **<0.0001** |
|  |  |  | 1 | 1.00 | **0.004** | **<0.0001** | **<0.0001** | **<0.0001** | **<0.0001** |
|  |  |  | 1.5 | 1.00 | **0.0005** | **<0.0001** | **<0.0001** | **<0.0001** | **<0.0001** |
|  |  |  | 2.5 | 1.00 | **<0.0001** | **<0.0001** | **<0.0001** | **<0.0001** | **<0.0001** |
|  |  |  | 5 | 1.00 | **<0.0001** | **<0.0001** | **<0.0001** | **<0.0001** | **<0.0001** |
| DifferenceEntropy | texture | glcm4 | 0.5 | 0.99 | 0.99 | 0.23 | **0.02** | **0.0002** | **<0.0001** |
|  |  |  | 1 | 1.00 | **0.0322** | **<0.0001** | **<0.0001** | **<0.0001** | **<0.0001** |
|  |  |  | 1.5 | 1.00 | **<0.0001** | **<0.0001** | **<0.0001** | **<0.0001** | **<0.0001** |
|  |  |  | 2.5 | 1.00 | **<0.0001** | **<0.0001** | **<0.0001** | **<0.0001** | **<0.0001** |
|  |  |  | 5 | 1.00 | **<0.0001** | **<0.0001** | **<0.0001** | **<0.0001** | **<0.0001** |
| DifferenceVariance | texture | glcm4 | 0.5 | 0.96 | **<0.0001** | **<0.0001** | **<0.0001** | **<0.0001** | **<0.0001** |
|  |  |  | 1 | 0.99 | **<0.0001** | **<0.0001** | **<0.0001** | **<0.0001** | **<0.0001** |
|  |  |  | 1.5 | 1.00 | **<0.0001** | **<0.0001** | **<0.0001** | **<0.0001** | **<0.0001** |
|  |  |  | 2.5 | 1.00 | **<0.0001** | **<0.0001** | **<0.0001** | **<0.0001** | **<0.0001** |
|  |  |  | 5 | 1.00 | **<0.0001** | **<0.0001** | **<0.0001** | **<0.0001** | **<0.0001** |
| Id | texture | glcm4 | 0.5 | 0.98 | **<0.0001** | **<0.0001** | **<0.0001** | **<0.0001** | **<0.0001** |
|  |  |  | 1 | 1.00 | **0.0001** | **<0.0001** | **<0.0001** | **<0.0001** | **<0.0001** |
|  |  |  | 1.5 | 1.00 | **0.002** | **<0.0001** | **<0.0001** | **<0.0001** | **<0.0001** |
|  |  |  | 2.5 | 1.00 | 0.59 | 0.07 | **0.02** | **0.008** | **0.0001** |
|  |  |  | 5 | 1.00 | **<0.0001** | **<0.0001** | **<0.0001** | **<0.0001** | **<0.0001** |
| Idm | texture | glcm4 | 0.5 | 0.98 | **<0.0001** | **<0.0001** | **<0.0001** | **<0.0001** | **<0.0001** |
|  |  |  | 1 | 1.00 | **<0.0001** | **<0.0001** | **<0.0001** | **<0.0001** | **<0.0001** |
|  |  |  | 1.5 | 1.00 | **0.0004** | **<0.0001** | **<0.0001** | **<0.0001** | **<0.0001** |
|  |  |  | 2.5 | 1.00 | 0.65 | 0.09 | **0.03** | **0.01** | **0.0003** |
|  |  |  | 5 | 1.00 | **<0.0001** | **<0.0001** | **<0.0001** | **<0.0001** | **<0.0001** |
| Idmn | texture | glcm4 | 0.5 | 0.97 | **<0.0001** | **<0.0001** | **<0.0001** | **<0.0001** | **<0.0001** |
|  |  |  | 1 | 1.00 | **0.0001** | **<0.0001** | **<0.0001** | **<0.0001** | **<0.0001** |
|  |  |  | 1.5 | 1.00 | 0.53 | **0.04** | **0.01** | **0.002** | **<0.0001** |
|  |  |  | 2.5 | 1.00 | 0.81 | 0.75 | 0.86 | 0.78 | 0.93 |
|  |  |  | 5 | 1.00 | 0.80 | 0.86 | 0.47 | 0.77 | 0.45 |
| Idn | texture | glcm4 | 0.5 | 0.96 | **<0.0001** | **<0.0001** | **<0.0001** | **<0.0001** | **<0.0001** |
|  |  |  | 1 | 1.00 | **<0.0001** | **<0.0001** | **<0.0001** | **<0.0001** | **<0.0001** |
|  |  |  | 1.5 | 1.00 | **0.0106** | **<0.0001** | **<0.0001** | **<0.0001** | **<0.0001** |
|  |  |  | 2.5 | 1.00 | 0.37 | 0.16 | 0.20 | 0.10 | **0.004** |
|  |  |  | 5 | 1.00 | 0.98 | 0.77 | 0.29 | 0.47 | 0.69 |
| Imc1 | texture | glcm4 | 0.5 | 0.88 | **<0.0001** | **<0.0001** | **<0.0001** | **<0.0001** | **<0.0001** |
|  |  |  | 1 | 0.98 | **<0.0001** | **<0.0001** | **<0.0001** | **<0.0001** | **<0.0001** |
|  |  |  | 1.5 | 1.00 | **<0.0001** | **<0.0001** | **<0.0001** | **<0.0001** | **<0.0001** |
|  |  |  | 2.5 | 1.00 | **0.008** | **<0.0001** | **<0.0001** | **<0.0001** | **<0.0001** |
|  |  |  | 5 | 1.00 | **<0.0001** | **<0.0001** | **<0.0001** | **<0.0001** | **<0.0001** |
| Imc2 | texture | glcm4 | 0.5 | 0.93 | **<0.0001** | **<0.0001** | **<0.0001** | **<0.0001** | **<0.0001** |
|  |  |  | 1 | 0.98 | **<0.0001** | **<0.0001** | **<0.0001** | **<0.0001** | **<0.0001** |
|  |  |  | 1.5 | 1.00 | **<0.0001** | **<0.0001** | **<0.0001** | **<0.0001** | **<0.0001** |
|  |  |  | 2.5 | 1.00 | **0.002** | **<0.0001** | **<0.0001** | **<0.0001** | **<0.0001** |
|  |  |  | 5 | 1.00 | **0.02** | **<0.0001** | **<0.0001** | **<0.0001** | **<0.0001** |
| InverseVariance | texture | glcm4 | 0.5 | 0.93 | **<0.0001** | **<0.0001** | **<0.0001** | **<0.0001** | **<0.0001** |
|  |  |  | 1 | 1.00 | **<0.0001** | **<0.0001** | **<0.0001** | **<0.0001** | **<0.0001** |
|  |  |  | 1.5 | 1.00 | **<0.0001** | **<0.0001** | **<0.0001** | **<0.0001** | **<0.0001** |
|  |  |  | 2.5 | 1.00 | **<0.0001** | **<0.0001** | **<0.0001** | **<0.0001** | **<0.0001** |
|  |  |  | 5 | 1.00 | **<0.0001** | **<0.0001** | **<0.0001** | **<0.0001** | **<0.0001** |
| JointEnergy | texture | glcm4 | 0.5 | 0.97 | **<0.0001** | **<0.0001** | **<0.0001** | **<0.0001** | **<0.0001** |
|  |  |  | 1 | 1.00 | **<0.0001** | **<0.0001** | **<0.0001** | **<0.0001** | **<0.0001** |
|  |  |  | 1.5 | 1.00 | **<0.0001** | **<0.0001** | **<0.0001** | **<0.0001** | **<0.0001** |
|  |  |  | 2.5 | 1.00 | **0.01** | **<0.0001** | **<0.0001** | **<0.0001** | **<0.0001** |
|  |  |  | 5 | 1.00 | 0.09 | **0.0001** | **<0.0001** | **<0.0001** | **<0.0001** |
| JointEntropy | texture | glcm4 | 0.5 | 0.99 | **0.0004** | **<0.0001** | **<0.0001** | **<0.0001** | **<0.0001** |
|  |  |  | 1 | 1.00 | 0.15 | **0.005** | **0.0011** | **0.0004** | **<0.0001** |
|  |  |  | 1.5 | 1.00 | 0.64 | 0.20 | 0.10 | **0.03** | **0.003** |
|  |  |  | 2.5 | 1.00 | **0.0002** | **<0.0001** | **<0.0001** | **<0.0001** | **<0.0001** |
|  |  |  | 5 | 1.00 | **<0.0001** | **<0.0001** | **<0.0001** | **<0.0001** | **<0.0001** |
| MaximumProbability | texture | glcm4 | 0.5 | 0.88 | **<0.0001** | **<0.0001** | **<0.0001** | **<0.0001** | **<0.0001** |
|  |  |  | 1 | 0.99 | **<0.0001** | **<0.0001** | **<0.0001** | **<0.0001** | **<0.0001** |
|  |  |  | 1.5 | 1.00 | **<0.0001** | **<0.0001** | **<0.0001** | **<0.0001** | **<0.0001** |
|  |  |  | 2.5 | 1.00 | **<0.0001** | **<0.0001** | **<0.0001** | **<0.0001** | **<0.0001** |
|  |  |  | 5 | 1.00 | 0.88 | 0.91 | 0.62 | 0.35 | **0.04** |
| MCC | texture | glcm4 | 0.5 | 0.92 | **<0.0001** | **<0.0001** | **<0.0001** | **<0.0001** | **<0.0001** |
|  |  |  | 1 | 0.99 | **<0.0001** | **<0.0001** | **<0.0001** | **<0.0001** | **<0.0001** |
|  |  |  | 1.5 | 1.00 | **<0.0001** | **<0.0001** | **<0.0001** | **<0.0001** | **<0.0001** |
|  |  |  | 2.5 | 1.00 | 0.52 | 0.41 | 0.65 | 0.21 | 0.25 |
|  |  |  | 5 | 1.00 | 0.13 | **0.0005** | **0.0001** | **<0.0001** | **<0.0001** |
| SumAverage | texture | glcm4 | 0.5 | 0.85 | **<0.0001** | **<0.0001** | **<0.0001** | **<0.0001** | **<0.0001** |
|  |  |  | 1 | 0.95 | **<0.0001** | **<0.0001** | **<0.0001** | **<0.0001** | **<0.0001** |
|  |  |  | 1.5 | 0.98 | 0.08 | **<0.0001** | **<0.0001** | **<0.0001** | **<0.0001** |
|  |  |  | 2.5 | 0.99 | 0.28 | **0.02** | **0.003** | **<0.0001** | **<0.0001** |
|  |  |  | 5 | 1.00 | 0.74 | 0.17 | **0.01** | **0.01** | **0.03** |
| SumEntropy | texture | glcm4 | 0.5 | 0.99 | 0.26 | 0.25 | 0.55 | 0.81 | **0.006** |
|  |  |  | 1 | 1.00 | 0.40 | **0.05** | **0.005** | **0.0002** | **<0.0001** |
|  |  |  | 1.5 | 1.00 | **0.002** | **<0.0001** | **<0.0001** | **<0.0001** | **<0.0001** |
|  |  |  | 2.5 | 1.00 | **<0.0001** | **<0.0001** | **<0.0001** | **<0.0001** | **<0.0001** |
|  |  |  | 5 | 1.00 | **<0.0001** | **<0.0001** | **<0.0001** | **<0.0001** | **<0.0001** |
| SumSquares | texture | glcm4 | 0.5 | 0.97 | **0.002** | **<0.0001** | **<0.0001** | **<0.0001** | **<0.0001** |
|  |  |  | 1 | 0.99 | **<0.0001** | **<0.0001** | **<0.0001** | **<0.0001** | **<0.0001** |
|  |  |  | 1.5 | 1.00 | **<0.0001** | **<0.0001** | **<0.0001** | **<0.0001** | **<0.0001** |
|  |  |  | 2.5 | 1.00 | **<0.0001** | **<0.0001** | **<0.0001** | **<0.0001** | **<0.0001** |
|  |  |  | 5 | 1.00 | **<0.0001** | **<0.0001** | **<0.0001** | **<0.0001** | **<0.0001** |
| Autocorrelation | texture | glcm7 | 0.5 | 0.86 | **<0.0001** | **<0.0001** | **<0.0001** | **<0.0001** | **<0.0001** |
|  |  |  | 1 | 0.95 | **<0.0001** | **<0.0001** | **<0.0001** | **<0.0001** | **<0.0001** |
|  |  |  | 1.5 | 0.99 | 0.09 | **<0.0001** | **<0.0001** | **<0.0001** | **<0.0001** |
|  |  |  | 2.5 | 0.99 | 0.37 | **0.04** | **0.005** | **<0.0001** | **<0.0001** |
|  |  |  | 5 | 1.00 | 0.73 | 0.18 | **0.009** | **0.01** | **0.02** |
| ClusterProminence | texture | glcm7 | 0.5 | 0.92 | **0.006** | **<0.0001** | **<0.0001** | **<0.0001** | **<0.0001** |
|  |  |  | 1 | 0.98 | **0.001** | **<0.0001** | **<0.0001** | **<0.0001** | **<0.0001** |
|  |  |  | 1.5 | 0.99 | **0.001** | **<0.0001** | **<0.0001** | **<0.0001** | **<0.0001** |
|  |  |  | 2.5 | 1.00 | 0.07 | **0.0001** | **<0.0001** | **<0.0001** | **<0.0001** |
|  |  |  | 5 | 1.00 | **0.001** | **<0.0001** | **<0.0001** | **<0.0001** | **<0.0001** |
| ClusterShade | texture | glcm7 | 0.5 | 0.97 | 0.34 | **0.03** | **0.005** | **0.0009** | **<0.0001** |
|  |  |  | 1 | 0.99 | 0.45 | 0.08 | **0.03** | **0.008** | **0.0003** |
|  |  |  | 1.5 | 1.00 | 0.80 | 0.49 | 0.44 | 0.35 | 0.20 |
|  |  |  | 2.5 | 1.00 | 0.99 | 0.93 | 0.98 | 0.96 | 0.86 |
|  |  |  | 5 | 1.00 | 0.07 | **0.0003** | **<0.0001** | **<0.0001** | **<0.0001** |
| ClusterTendency | texture | glcm7 | 0.5 | 0.97 | **0.001** | **<0.0001** | **<0.0001** | **<0.0001** | **<0.0001** |
|  |  |  | 1 | 0.99 | **<0.0001** | **<0.0001** | **<0.0001** | **<0.0001** | **<0.0001** |
|  |  |  | 1.5 | 1.00 | **<0.0001** | **<0.0001** | **<0.0001** | **<0.0001** | **<0.0001** |
|  |  |  | 2.5 | 1.00 | **<0.0001** | **<0.0001** | **<0.0001** | **<0.0001** | **<0.0001** |
|  |  |  | 5 | 1.00 | **<0.0001** | **<0.0001** | **<0.0001** | **<0.0001** | **<0.0001** |
| Contrast | texture | glcm7 | 0.5 | 0.97 | **0.002** | **<0.0001** | **<0.0001** | **<0.0001** | **<0.0001** |
|  |  |  | 1 | 0.99 | **<0.0001** | **<0.0001** | **<0.0001** | **<0.0001** | **<0.0001** |
|  |  |  | 1.5 | 1.00 | **<0.0001** | **<0.0001** | **<0.0001** | **<0.0001** | **<0.0001** |
|  |  |  | 2.5 | 1.00 | **<0.0001** | **<0.0001** | **<0.0001** | **<0.0001** | **<0.0001** |
|  |  |  | 5 | 1.00 | **<0.0001** | **<0.0001** | **<0.0001** | **<0.0001** | **<0.0001** |
| Correlation | texture | glcm7 | 0.5 | 0.99 | 0.35 | 0.54 | 0.90 | 0.67 | 0.11 |
|  |  |  | 1 | 1.00 | 0.24 | **0.003** | **0.0001** | **<0.0001** | **<0.0001** |
|  |  |  | 1.5 | 1.00 | **0.01** | **<0.0001** | **<0.0001** | **<0.0001** | **<0.0001** |
|  |  |  | 2.5 | 1.00 | **<0.0001** | **<0.0001** | **<0.0001** | **<0.0001** | **<0.0001** |
|  |  |  | 5 | 1.00 | **<0.0001** | **<0.0001** | **<0.0001** | **<0.0001** | **<0.0001** |
| DifferenceAverage | texture | glcm7 | 0.5 | 0.99 | 0.86 | 0.09 | **0.004** | **<0.0001** | **<0.0001** |
|  |  |  | 1 | 1.00 | **0.0008** | **<0.0001** | **<0.0001** | **<0.0001** | **<0.0001** |
|  |  |  | 1.5 | 1.00 | **<0.0001** | **<0.0001** | **<0.0001** | **<0.0001** | **<0.0001** |
|  |  |  | 2.5 | 1.00 | **<0.0001** | **<0.0001** | **<0.0001** | **<0.0001** | **<0.0001** |
|  |  |  | 5 | 1.00 | **<0.0001** | **<0.0001** | **<0.0001** | **<0.0001** | **<0.0001** |
| DifferenceEntropy | texture | glcm7 | 0.5 | 0.99 | 0.57 | **0.014** | **0.0001** | **<0.0001** | **<0.0001** |
|  |  |  | 1 | 1.00 | **0.001** | **<0.0001** | **<0.0001** | **<0.0001** | **<0.0001** |
|  |  |  | 1.5 | 1.00 | **<0.0001** | **<0.0001** | **<0.0001** | **<0.0001** | **<0.0001** |
|  |  |  | 2.5 | 1.00 | **<0.0001** | **<0.0001** | **<0.0001** | **<0.0001** | **<0.0001** |
|  |  |  | 5 | 1.00 | **<0.0001** | **<0.0001** | **<0.0001** | **<0.0001** | **<0.0001** |
| DifferenceVariance | texture | glcm7 | 0.5 | 0.95 | **<0.0001** | **<0.0001** | **<0.0001** | **<0.0001** | **<0.0001** |
|  |  |  | 1 | 0.99 | **<0.0001** | **<0.0001** | **<0.0001** | **<0.0001** | **<0.0001** |
|  |  |  | 1.5 | 1.00 | **<0.0001** | **<0.0001** | **<0.0001** | **<0.0001** | **<0.0001** |
|  |  |  | 2.5 | 1.00 | **<0.0001** | **<0.0001** | **<0.0001** | **<0.0001** | **<0.0001** |
|  |  |  | 5 | 1.00 | **<0.0001** | **<0.0001** | **<0.0001** | **<0.0001** | **<0.0001** |
| Id | texture | glcm7 | 0.5 | 0.99 | **<0.0001** | **<0.0001** | **<0.0001** | **<0.0001** | **<0.0001** |
|  |  |  | 1 | 1.00 | **0.003** | **<0.0001** | **<0.0001** | **<0.0001** | **<0.0001** |
|  |  |  | 1.5 | 1.00 | 0.42 | 0.08 | **0.02** | **0.008** | **0.0003** |
|  |  |  | 2.5 | 1.00 | 0.41 | **0.02** | **0.002** | **0.0003** | **<0.0001** |
|  |  |  | 5 | 1.00 | **<0.0001** | **<0.0001** | **<0.0001** | **<0.0001** | **<0.0001** |
| Idm | texture | glcm7 | 0.5 | 0.99 | **<0.0001** | **<0.0001** | **<0.0001** | **<0.0001** | **<0.0001** |
|  |  |  | 1 | 1.00 | **0.0002** | **<0.0001** | **<0.0001** | **<0.0001** | **<0.0001** |
|  |  |  | 1.5 | 1.00 | 0.12 | **0.0009** | **<0.0001** | **<0.0001** | **<0.0001** |
|  |  |  | 2.5 | 1.00 | 0.66 | 0.13 | **0.04** | **0.02** | **0.001** |
|  |  |  | 5 | 1.00 | **<0.0001** | **<0.0001** | **<0.0001** | **<0.0001** | **<0.0001** |
| Idmn | texture | glcm7 | 0.5 | 0.97 | **<0.0001** | **<0.0001** | **<0.0001** | **<0.0001** | **<0.0001** |
|  |  |  | 1 | 1.00 | **0.0001** | **<0.0001** | **<0.0001** | **<0.0001** | **<0.0001** |
|  |  |  | 1.5 | 1.00 | 0.29 | **0.003** | **0.0004** | **<0.0001** | **<0.0001** |
|  |  |  | 2.5 | 1.00 | 0.49 | 0.31 | 0.48 | 0.41 | 0.10 |
|  |  |  | 5 | 1.00 | 0.91 | 0.91 | 0.29 | 0.49 | 0.78 |
| Idn | texture | glcm7 | 0.5 | 0.96 | **<0.0001** | **<0.0001** | **<0.0001** | **<0.0001** | **<0.0001** |
|  |  |  | 1 | 1.00 | **<0.0001** | **<0.0001** | **<0.0001** | **<0.0001** | **<0.0001** |
|  |  |  | 1.5 | 1.00 | **0.02** | **<0.0001** | **<0.0001** | **<0.0001** | **<0.0001** |
|  |  |  | 2.5 | 1.00 | 0.25 | 0.07 | 0.07 | **0.02** | **0.0001** |
|  |  |  | 5 | 1.00 | 0.88 | 0.59 | 0.18 | 0.27 | 0.94 |
| Imc1 | texture | glcm7 | 0.5 | 0.92 | **<0.0001** | **<0.0001** | **<0.0001** | **<0.0001** | **<0.0001** |
|  |  |  | 1 | 0.98 | **<0.0001** | **<0.0001** | **<0.0001** | **<0.0001** | **<0.0001** |
|  |  |  | 1.5 | 1.00 | **<0.0001** | **<0.0001** | **<0.0001** | **<0.0001** | **<0.0001** |
|  |  |  | 2.5 | 1.00 | **<0.0001** | **<0.0001** | **<0.0001** | **<0.0001** | **<0.0001** |
|  |  |  | 5 | 1.00 | 0.17 | **0.0002** | **<0.0001** | **<0.0001** | **<0.0001** |
| Imc2 | texture | glcm7 | 0.5 | 0.95 | **<0.0001** | **<0.0001** | **<0.0001** | **<0.0001** | **<0.0001** |
|  |  |  | 1 | 0.99 | **<0.0001** | **<0.0001** | **<0.0001** | **<0.0001** | **<0.0001** |
|  |  |  | 1.5 | 1.00 | **<0.0001** | **<0.0001** | **<0.0001** | **<0.0001** | **<0.0001** |
|  |  |  | 2.5 | 1.00 | **<0.0001** | **<0.0001** | **<0.0001** | **<0.0001** | **<0.0001** |
|  |  |  | 5 | 1.00 | 0.59 | 0.15 | 0.09 | **0.02** | **0.0001** |
| InverseVariance | texture | glcm7 | 0.5 | 0.95 | **<0.0001** | **<0.0001** | **<0.0001** | **<0.0001** | **<0.0001** |
|  |  |  | 1 | 1.00 | **<0.0001** | **<0.0001** | **<0.0001** | **<0.0001** | **<0.0001** |
|  |  |  | 1.5 | 1.00 | **<0.0001** | **<0.0001** | **<0.0001** | **<0.0001** | **<0.0001** |
|  |  |  | 2.5 | 1.00 | **<0.0001** | **<0.0001** | **<0.0001** | **<0.0001** | **<0.0001** |
|  |  |  | 5 | 1.00 | **<0.0001** | **<0.0001** | **<0.0001** | **<0.0001** | **<0.0001** |
| JointEnergy | texture | glcm7 | 0.5 | 0.97 | **<0.0001** | **<0.0001** | **<0.0001** | **<0.0001** | **<0.0001** |
|  |  |  | 1 | 1.00 | **<0.0001** | **<0.0001** | **<0.0001** | **<0.0001** | **<0.0001** |
|  |  |  | 1.5 | 1.00 | **<0.0001** | **<0.0001** | **<0.0001** | **<0.0001** | **<0.0001** |
|  |  |  | 2.5 | 1.00 | **0.03** | **<0.0001** | **<0.0001** | **<0.0001** | **<0.0001** |
|  |  |  | 5 | 1.00 | 0.20 | **0.002** | **0.0001** | **<0.0001** | **<0.0001** |
| JointEntropy | texture | glcm7 | 0.5 | 0.99 | **0.0006** | **<0.0001** | **<0.0001** | **<0.0001** | **<0.0001** |
|  |  |  | 1 | 1.00 | 0.27 | **0.03** | **0.02** | **0.01** | **0.007** |
|  |  |  | 1.5 | 1.00 | 0.35 | **0.02** | **0.005** | **0.0004** | **<0.0001** |
|  |  |  | 2.5 | 1.00 | **0.0001** | **<0.0001** | **<0.0001** | **<0.0001** | **<0.0001** |
|  |  |  | 5 | 1.00 | **<0.0001** | **<0.0001** | **<0.0001** | **<0.0001** | **<0.0001** |
| MaximumProbability | texture | glcm7 | 0.5 | 0.90 | **<0.0001** | **<0.0001** | **<0.0001** | **<0.0001** | **<0.0001** |
|  |  |  | 1 | 0.99 | **<0.0001** | **<0.0001** | **<0.0001** | **<0.0001** | **<0.0001** |
|  |  |  | 1.5 | 1.00 | **<0.0001** | **<0.0001** | **<0.0001** | **<0.0001** | **<0.0001** |
|  |  |  | 2.5 | 1.00 | **<0.0001** | **<0.0001** | **<0.0001** | **<0.0001** | **<0.0001** |
|  |  |  | 5 | 1.00 | 0.88 | 0.59 | 0.25 | 0.16 | **0.02** |
| MCC | texture | glcm7 | 0.5 | 0.95 | **<0.0001** | **<0.0001** | **<0.0001** | **<0.0001** | **<0.0001** |
|  |  |  | 1 | 0.99 | **<0.0001** | **<0.0001** | **<0.0001** | **<0.0001** | **<0.0001** |
|  |  |  | 1.5 | 1.00 | **0.0001** | **<0.0001** | **<0.0001** | **<0.0001** | **<0.0001** |
|  |  |  | 2.5 | 1.00 | **0.01** | **0.001** | **0.001** | **0.0007** | **0.0002** |
|  |  |  | 5 | 1.00 | 0.10 | **0.002** | **0.0001** | **<0.0001** | **<0.0001** |
| SumAverage | texture | glcm7 | 0.5 | 0.85 | **<0.0001** | **<0.0001** | **<0.0001** | **<0.0001** | **<0.0001** |
|  |  |  | 1 | 0.95 | **<0.0001** | **<0.0001** | **<0.0001** | **<0.0001** | **<0.0001** |
|  |  |  | 1.5 | 0.98 | 0.08 | **<0.0001** | **<0.0001** | **<0.0001** | **<0.0001** |
|  |  |  | 2.5 | 0.99 | 0.26 | **0.01** | **0.002** | **<0.0001** | **<0.0001** |
|  |  |  | 5 | 1.00 | 0.73 | 0.16 | **0.01** | **0.01** | **0.02** |
| SumEntropy | texture | glcm7 | 0.5 | 0.99 | 0.41 | 0.60 | 0.91 | 0.28 | **0.0001** |
|  |  |  | 1 | 1.00 | 0.14 | **0.001** | **<0.0001** | **<0.0001** | **<0.0001** |
|  |  |  | 1.5 | 1.00 | **0.0009** | **<0.0001** | **<0.0001** | **<0.0001** | **<0.0001** |
|  |  |  | 2.5 | 1.00 | **<0.0001** | **<0.0001** | **<0.0001** | **<0.0001** | **<0.0001** |
|  |  |  | 5 | 1.00 | **<0.0001** | **<0.0001** | **<0.0001** | **<0.0001** | **<0.0001** |
| SumSquares | texture | glcm7 | 0.5 | 0.97 | **0.002** | **<0.0001** | **<0.0001** | **<0.0001** | **<0.0001** |
|  |  |  | 1 | 0.99 | **<0.0001** | **<0.0001** | **<0.0001** | **<0.0001** | **<0.0001** |
|  |  |  | 1.5 | 1.00 | **<0.0001** | **<0.0001** | **<0.0001** | **<0.0001** | **<0.0001** |
|  |  |  | 2.5 | 1.00 | **<0.0001** | **<0.0001** | **<0.0001** | **<0.0001** | **<0.0001** |
|  |  |  | 5 | 1.00 | **<0.0001** | **<0.0001** | **<0.0001** | **<0.0001** | **<0.0001** |
| DependenceEntropy | texture | gldm | 0.5 | 0.99 | **0.0007** | **<0.0001** | **<0.0001** | **<0.0001** | **<0.0001** |
|  |  |  | 1 | 1.00 | 0.26 | **0.001** | **<0.0001** | **<0.0001** | **<0.0001** |
|  |  |  | 1.5 | 1.00 | **0.007** | **<0.0001** | **<0.0001** | **<0.0001** | **<0.0001** |
|  |  |  | 2.5 | 1.00 | 0.39 | 0.10 | **0.05** | 0.16 | 0.27 |
|  |  |  | 5 | 1.00 | **0.002** | **<0.0001** | **<0.0001** | **<0.0001** | **<0.0001** |
| DependenceNonUniformityNormalized | texture | gldm | 0.5 | **0.74*** | **<0.0001** | **<0.0001** | **<0.0001** | **<0.0001** | **<0.0001** |
|  |  |  | 1 | 1.00 | **<0.0001** | **<0.0001** | **<0.0001** | **<0.0001** | **<0.0001** |
|  |  |  | 1.5 | 1.00 | **0.007** | **<0.0001** | **<0.0001** | **<0.0001** | **<0.0001** |
|  |  |  | 2.5 | 1.00 | 0.24 | **0.006** | **<0.0001** | **<0.0001** | **<0.0001** |
|  |  |  | 5 | 1.00 | 0.20 | 0.14 | 0.34 | 0.59 | 0.55 |
| DependenceVariance | texture | gldm | 0.5 | **0.54*** | **<0.0001** | **<0.0001** | **<0.0001** | **<0.0001** | **<0.0001** |
|  |  |  | 1 | 0.92 | **<0.0001** | **<0.0001** | **<0.0001** | **<0.0001** | **<0.0001** |
|  |  |  | 1.5 | 1.00 | **<0.0001** | **<0.0001** | **<0.0001** | **<0.0001** | **<0.0001** |
|  |  |  | 2.5 | 1.00 | **0.0003** | **<0.0001** | **<0.0001** | **<0.0001** | **<0.0001** |
|  |  |  | 5 | 1.00 | **0.05** | **0.004** | **0.002** | **0.001** | **<0.0001** |
| GrayLevelNonUniformity_modified | texture | gldm | 0.5 | 0.98 | **<0.0001** | **<0.0001** | **<0.0001** | **<0.0001** | **<0.0001** |
|  |  |  | 1 | 1.00 | **0.0001** | **<0.0001** | **<0.0001** | **<0.0001** | **<0.0001** |
|  |  |  | 1.5 | 1.00 | 0.06 | **0.0003** | **<0.0001** | **<0.0001** | **<0.0001** |
|  |  |  | 2.5 | 1.00 | 0.14 | **0.002** | **0.0001** | **<0.0001** | **<0.0001** |
|  |  |  | 5 | 1.00 | **<0.0001** | **<0.0001** | **<0.0001** | **<0.0001** | **<0.0001** |
| GrayLevelVariance | texture | gldm | 0.5 | 0.96 | **0.0001** | **<0.0001** | **<0.0001** | **<0.0001** | **<0.0001** |
|  |  |  | 1 | 0.99 | **<0.0001** | **<0.0001** | **<0.0001** | **<0.0001** | **<0.0001** |
|  |  |  | 1.5 | 1.00 | **<0.0001** | **<0.0001** | **<0.0001** | **<0.0001** | **<0.0001** |
|  |  |  | 2.5 | 1.00 | **<0.0001** | **<0.0001** | **<0.0001** | **<0.0001** | **<0.0001** |
|  |  |  | 5 | 1.00 | **<0.0001** | **<0.0001** | **<0.0001** | **<0.0001** | **<0.0001** |
| HighGrayLevel  Emphasis | texture | gldm | 0.5 | 0.86 | **<0.0001** | **<0.0001** | **<0.0001** | **<0.0001** | **<0.0001** |
|  |  |  | 1 | 0.96 | **<0.0001** | **<0.0001** | **<0.0001** | **<0.0001** | **<0.0001** |
|  |  |  | 1.5 | 0.99 | 0.08 | **<0.0001** | **<0.0001** | **<0.0001** | **<0.0001** |
|  |  |  | 2.5 | 0.99 | 0.36 | **0.03** | **0.004** | **<0.0001** | **<0.0001** |
|  |  |  | 5 | 1.00 | 0.73 | 0.18 | **0.007** | **0.008** | **0.01** |
| LargeDependence  Emphasis | texture | gldm | 0.5 | **0.74*** | **<0.0001** | **<0.0001** | **<0.0001** | **<0.0001** | **<0.0001** |
|  |  |  | 1 | 0.98 | **<0.0001** | **<0.0001** | **<0.0001** | **<0.0001** | **<0.0001** |
|  |  |  | 1.5 | 1.00 | **<0.0001** | **<0.0001** | **<0.0001** | **<0.0001** | **<0.0001** |
|  |  |  | 2.5 | 1.00 | 0.72 | 0.91 | 0.71 | 0.44 | 0.08 |
|  |  |  | 5 | 1.00 | **0.0002** | **<0.0001** | **<0.0001** | **<0.0001** | **<0.0001** |
| LargeDependenceHighGrayLevelEmphasis | texture | gldm | 0.5 | **0.69*** | **<0.0001** | **<0.0001** | **<0.0001** | **<0.0001** | **<0.0001** |
|  |  |  | 1 | 0.93 | **<0.0001** | **<0.0001** | **<0.0001** | **<0.0001** | **<0.0001** |
|  |  |  | 1.5 | 0.99 | **0.005** | **<0.0001** | **<0.0001** | **<0.0001** | **<0.0001** |
|  |  |  | 2.5 | 1.00 | 0.32 | **0.04** | **0.002** | **<0.0001** | **<0.0001** |
|  |  |  | 5 | 1.00 | 0.44 | 0.10 | **0.04** | 0.053 | 0.08 |
| LargeDependenceLowGrayLevelEmphasis | texture | gldm | 0.5 | 0.94 | 0.39 | **0.04** | **0.0006** | **<0.0001** | **<0.0001** |
|  |  |  | 1 | 0.98 | 0.20 | **0.05** | **0.01** | **0.002** | **<0.0001** |
|  |  |  | 1.5 | 0.99 | 0.66 | **0.0016** | **0.0003** | **0.0004** | **<0.0001** |
|  |  |  | 2.5 | 0.98 | 0.45 | **0.05** | **0.03** | **0.009** | **0.0001** |
|  |  |  | 5 | 0.99 | 0.96 | 0.46 | 0.14 | 0.21 | 0.46 |
| LowGrayLevel  Emphasis | texture | gldm | 0.5 | **0.82*** | **<0.0001** | **<0.0001** | **<0.0001** | **<0.0001** | **<0.0001** |
|  |  |  | 1 | 0.92 | **0.0016** | **<0.0001** | **<0.0001** | **<0.0001** | **<0.0001** |
|  |  |  | 1.5 | 0.97 | 0.67 | **0.008** | **0.001** | **0.001** | **<0.0001** |
|  |  |  | 2.5 | 0.98 | 0.68 | 0.20 | 0.29 | 0.19 | **0.05** |
|  |  |  | 5 | 0.99 | 0.96 | 0.56 | 0.15 | 0.24 | 0.49 |
| SmallDependence  Emphasis | texture | gldm | 0.5 | 0.97 | **0.0005** | **<0.0001** | **<0.0001** | **<0.0001** | **<0.0001** |
|  |  |  | 1 | 1.00 | 0.70 | **0.03** | **0.006** | **0.0004** | **<0.0001** |
|  |  |  | 1.5 | 1.00 | **0.0005** | **<0.0001** | **<0.0001** | **<0.0001** | **<0.0001** |
|  |  |  | 2.5 | 1.00 | **<0.0001** | **<0.0001** | **<0.0001** | **<0.0001** | **<0.0001** |
|  |  |  | 5 | 1.00 | **0.002** | **<0.0001** | **<0.0001** | **<0.0001** | **<0.0001** |
| SmallDependenceHighGrayLevelEmphasis | texture | gldm | 0.5 | 0.93 | 0.06 | **<0.0001** | **<0.0001** | **<0.0001** | **<0.0001** |
|  |  |  | 1 | 0.98 | **0.0009** | **<0.0001** | **<0.0001** | **<0.0001** | **<0.0001** |
|  |  |  | 1.5 | 0.99 | **0.05** | **<0.0001** | **<0.0001** | **<0.0001** | **<0.0001** |
|  |  |  | 2.5 | 1.00 | 0.10 | **0.0001** | **<0.0001** | **<0.0001** | **<0.0001** |
|  |  |  | 5 | 1.00 | 0.66 | 0.18 | **0.002** | **0.001** | **0.0003** |
| SmallDependenceLowGrayLevelEmphasis | texture | gldm | 0.5 | 0.85 | **0.0001** | **<0.0001** | **<0.0001** | **<0.0001** | **<0.0001** |
|  |  |  | 1 | 0.94 | 0.06 | **0.0005** | **0.0001** | **<0.0001** | **<0.0001** |
|  |  |  | 1.5 | 0.98 | 0.96 | 0.46 | 0.29 | 0.52 | **0.05** |
|  |  |  | 2.5 | 0.99 | 0.96 | 0.53 | 0.98 | 0.75 | 0.93 |
|  |  |  | 5 | 0.99 | 0.98 | 0.71 | 0.23 | 0.68 | 0.81 |
| GrayLevelNonUniformityNormalized | texture | glrlm | 0.5 | 0.99 | 0.38 | 0.98 | 0.16 | **0.0004** | **<0.0001** |
|  |  |  | 1 | 1.00 | **<0.0001** | **<0.0001** | **<0.0001** | **<0.0001** | **<0.0001** |
|  |  |  | 1.5 | 1.00 | **<0.0001** | **<0.0001** | **<0.0001** | **<0.0001** | **<0.0001** |
|  |  |  | 2.5 | 1.00 | **<0.0001** | **<0.0001** | **<0.0001** | **<0.0001** | **<0.0001** |
|  |  |  | 5 | 1.00 | **<0.0001** | **<0.0001** | **<0.0001** | **<0.0001** | **<0.0001** |
| GrayLevelVariance | texture | glrlm | 0.5 | 0.92 | **<0.0001** | **<0.0001** | **<0.0001** | **<0.0001** | **<0.0001** |
|  |  |  | 1 | 0.98 | **<0.0001** | **<0.0001** | **<0.0001** | **<0.0001** | **<0.0001** |
|  |  |  | 1.5 | 0.99 | **<0.0001** | **<0.0001** | **<0.0001** | **<0.0001** | **<0.0001** |
|  |  |  | 2.5 | 1.00 | **<0.0001** | **<0.0001** | **<0.0001** | **<0.0001** | **<0.0001** |
|  |  |  | 5 | 1.00 | **<0.0001** | **<0.0001** | **<0.0001** | **<0.0001** | **<0.0001** |
| HighGrayLevelRun  Emphasis | texture | glrlm | 0.5 | 0.86 | **<0.0001** | **<0.0001** | **<0.0001** | **<0.0001** | **<0.0001** |
|  |  |  | 1 | 0.96 | **<0.0001** | **<0.0001** | **<0.0001** | **<0.0001** | **<0.0001** |
|  |  |  | 1.5 | 0.99 | 0.13 | **<0.0001** | **<0.0001** | **<0.0001** | **<0.0001** |
|  |  |  | 2.5 | 0.99 | 0.41 | **0.05** | **0.01** | **0.0001** | **<0.0001** |
|  |  |  | 5 | 1.00 | 0.78 | 0.21 | **0.01** | **0.01** | **0.02** |
| LongRunEmphasis | texture | glrlm | 0.5 | **0.68*** | **<0.0001** | **<0.0001** | **<0.0001** | **<0.0001** | **<0.0001** |
|  |  |  | 1 | 0.95 | **<0.0001** | **<0.0001** | **<0.0001** | **<0.0001** | **<0.0001** |
|  |  |  | 1.5 | 0.99 | **0.0001** | **<0.0001** | **<0.0001** | **<0.0001** | **<0.0001** |
|  |  |  | 2.5 | 1.00 | **0.03** | **<0.0001** | **<0.0001** | **<0.0001** | **<0.0001** |
|  |  |  | 5 | 1.00 | 0.08 | **<0.0001** | **<0.0001** | **<0.0001** | **<0.0001** |
| LongRunHighGray  LevelEmphasis | texture | glrlm | 0.5 | **0.63*** | **<0.0001** | **<0.0001** | **<0.0001** | **<0.0001** | **<0.0001** |
|  |  |  | 1 | 0.90 | **<0.0001** | **<0.0001** | **<0.0001** | **<0.0001** | **<0.0001** |
|  |  |  | 1.5 | 0.98 | **0.002** | **<0.0001** | **<0.0001** | **<0.0001** | **<0.0001** |
|  |  |  | 2.5 | 1.00 | 0.15 | **0.01** | **0.0001** | **<0.0001** | **<0.0001** |
|  |  |  | 5 | 1.00 | 0.20 | 0.06 | **0.04** | **0.047** | 0.054 |
| LongRunLowGray  LevelEmphasis | texture | glrlm | 0.5 | 0.93 | 0.86 | 0.51 | 0.86 | 0.52 | **0.001** |
|  |  |  | 1 | 0.98 | 0.46 | **0.01** | **0.001** | **0.0001** | **<0.0001** |
|  |  |  | 1.5 | 0.99 | 0.28 | 0.94 | 0.58 | 0.14 | 0.14 |
|  |  |  | 2.5 | 0.99 | 0.22 | 0.10 | **0.05** | **0.03** | **0.002** |
|  |  |  | 5 | 1.00 | 0.53 | 0.16 | 0.06 | 0.10 | 0.23 |
| LowGrayLevelRun  Emphasis | texture | glrlm | 0.5 | **0.84*** | **<0.0001** | **<0.0001** | **<0.0001** | **<0.0001** | **<0.0001** |
|  |  |  | 1 | 0.92 | **0.0222** | **<0.0001** | **<0.0001** | **<0.0001** | **<0.0001** |
|  |  |  | 1.5 | 0.97 | 0.85 | **0.03** | **0.009** | **0.02** | **0.0001** |
|  |  |  | 2.5 | 0.98 | 0.45 | 0.20 | 0.27 | 0.19 | **0.04** |
|  |  |  | 5 | 0.99 | 0.84 | 0.37 | 0.09 | 0.17 | 0.36 |
| RunEntropy | texture | glrlm | 0.5 | **0.76*** | **<0.0001** | **<0.0001** | **<0.0001** | **<0.0001** | **<0.0001** |
|  |  |  | 1 | 0.87 | **<0.0001** | **<0.0001** | **<0.0001** | **<0.0001** | **<0.0001** |
|  |  |  | 1.5 | 0.99 | **<0.0001** | **<0.0001** | **<0.0001** | **<0.0001** | **<0.0001** |
|  |  |  | 2.5 | 1.00 | **<0.0001** | **<0.0001** | **<0.0001** | **<0.0001** | **<0.0001** |
|  |  |  | 5 | 1.00 | 0.87 | 0.29 | 0.13 | **0.03** | **0.002** |
| RunLengthNon  UniformityNormalized | texture | glrlm | 0.5 | 0.92 | **<0.0001** | **<0.0001** | **<0.0001** | **<0.0001** | **<0.0001** |
|  |  |  | 1 | 1.00 | **0.004** | **<0.0001** | **<0.0001** | **<0.0001** | **<0.0001** |
|  |  |  | 1.5 | 1.00 | **<0.0001** | **<0.0001** | **<0.0001** | **<0.0001** | **<0.0001** |
|  |  |  | 2.5 | 1.00 | **<0.0001** | **<0.0001** | **<0.0001** | **<0.0001** | **<0.0001** |
|  |  |  | 5 | 1.00 | **<0.0001** | **<0.0001** | **<0.0001** | **<0.0001** | **<0.0001** |
| RunPercentage | texture | glrlm | 0.5 | **0.84*** | **<0.0001** | **<0.0001** | **<0.0001** | **<0.0001** | **<0.0001** |
|  |  |  | 1 | 0.99 | **<0.0001** | **<0.0001** | **<0.0001** | **<0.0001** | **<0.0001** |
|  |  |  | 1.5 | 1.00 | **<0.0001** | **<0.0001** | **<0.0001** | **<0.0001** | **<0.0001** |
|  |  |  | 2.5 | 1.00 | 0.23 | **0.0004** | **<0.0001** | **<0.0001** | **<0.0001** |
|  |  |  | 5 | 1.00 | **<0.0001** | **<0.0001** | **<0.0001** | **<0.0001** | **<0.0001** |
| RunVariance | texture | glrlm | 0.5 | **0.54*** | **<0.0001** | **<0.0001** | **<0.0001** | **<0.0001** | **<0.0001** |
|  |  |  | 1 | 0.92 | **<0.0001** | **<0.0001** | **<0.0001** | **<0.0001** | **<0.0001** |
|  |  |  | 1.5 | 0.99 | **<0.0001** | **<0.0001** | **<0.0001** | **<0.0001** | **<0.0001** |
|  |  |  | 2.5 | 1.00 | **0.01** | **<0.0001** | **<0.0001** | **<0.0001** | **<0.0001** |
|  |  |  | 5 | 1.00 | 0.95 | 0.21 | 0.08 | **0.003** | **0.002** |
| ShortRunEmphasis | texture | glrlm | 0.5 | 0.92 | **<0.0001** | **<0.0001** | **<0.0001** | **<0.0001** | **<0.0001** |
|  |  |  | 1 | 1.00 | **0.002** | **<0.0001** | **<0.0001** | **<0.0001** | **<0.0001** |
|  |  |  | 1.5 | 1.00 | **<0.0001** | **<0.0001** | **<0.0001** | **<0.0001** | **<0.0001** |
|  |  |  | 2.5 | 1.00 | **<0.0001** | **<0.0001** | **<0.0001** | **<0.0001** | **<0.0001** |
|  |  |  | 5 | 1.00 | **<0.0001** | **<0.0001** | **<0.0001** | **<0.0001** | **<0.0001** |
| ShortRunHighGray  LevelEmphasis | texture | glrlm | 0.5 | 0.90 | **0.0008** | **<0.0001** | **<0.0001** | **<0.0001** | **<0.0001** |
|  |  |  | 1 | 0.97 | **0.0005** | **<0.0001** | **<0.0001** | **<0.0001** | **<0.0001** |
|  |  |  | 1.5 | 0.99 | 0.10 | **<0.0001** | **<0.0001** | **<0.0001** | **<0.0001** |
|  |  |  | 2.5 | 0.99 | 0.35 | **0.02** | **0.004** | **<0.0001** | **<0.0001** |
|  |  |  | 5 | 1.00 | 0.81 | 0.23 | **0.006** | **0.007** | **0.006** |
| ShortRunLowGray  LevelEmphasis | texture | glrlm | 0.5 | **0.81*** | **<0.0001** | **<0.0001** | **<0.0001** | **<0.0001** | **<0.0001** |
|  |  |  | 1 | 0.92 | **0.0338** | **<0.0001** | **<0.0001** | **<0.0001** | **<0.0001** |
|  |  |  | 1.5 | 0.97 | 0.94 | 0.08 | **0.03** | 0.08 | **0.001** |
|  |  |  | 2.5 | 0.98 | 0.49 | 0.28 | 0.44 | 0.35 | 0.12 |
|  |  |  | 5 | 0.99 | 0.89 | 0.45 | 0.11 | 0.22 | 0.47 |
| GrayLevelNon  UniformityNormalized | texture | glszm | 0.5 | 0.89 | **<0.0001** | **<0.0001** | **<0.0001** | **<0.0001** | **<0.0001** |
|  |  |  | 1 | 0.98 | **<0.0001** | **<0.0001** | **<0.0001** | **<0.0001** | **<0.0001** |
|  |  |  | 1.5 | 0.99 | **<0.0001** | **<0.0001** | **<0.0001** | **<0.0001** | **<0.0001** |
|  |  |  | 2.5 | 1.00 | **<0.0001** | **<0.0001** | **<0.0001** | **<0.0001** | **<0.0001** |
|  |  |  | 5 | 1.00 | **0.01** | **<0.0001** | **<0.0001** | **<0.0001** | **<0.0001** |
| GrayLevelVariance | texture | glszm | 0.5 | **0.83*** | **<0.0001** | **<0.0001** | **<0.0001** | **<0.0001** | **<0.0001** |
|  |  |  | 1 | 0.96 | **<0.0001** | **<0.0001** | **<0.0001** | **<0.0001** | **<0.0001** |
|  |  |  | 1.5 | 0.99 | **<0.0001** | **<0.0001** | **<0.0001** | **<0.0001** | **<0.0001** |
|  |  |  | 2.5 | 1.00 | **<0.0001** | **<0.0001** | **<0.0001** | **<0.0001** | **<0.0001** |
|  |  |  | 5 | 1.00 | **0.05** | **0.0001** | **<0.0001** | **<0.0001** | **<0.0001** |
| HighGrayLevelZone  Emphasis | texture | glszm | 0.5 | 0.87 | **<0.0001** | **<0.0001** | **<0.0001** | **<0.0001** | **<0.0001** |
|  |  |  | 1 | 0.97 | **<0.0001** | **<0.0001** | **<0.0001** | **<0.0001** | **<0.0001** |
|  |  |  | 1.5 | 0.99 | 0.14 | **<0.0001** | **<0.0001** | **<0.0001** | **<0.0001** |
|  |  |  | 2.5 | 0.99 | 0.43 | 0.06 | **0.02** | **0.0002** | **<0.0001** |
|  |  |  | 5 | 1.00 | 0.74 | 0.23 | **0.01** | **0.02** | **0.02** |
| LargeAreaEmphasis | texture | glszm | 0.5 | 0.97 | **0.002** | **<0.0001** | **<0.0001** | **<0.0001** | **<0.0001** |
|  |  |  | 1 | 0.99 | **0.01** | **<0.0001** | **<0.0001** | **<0.0001** | **<0.0001** |
|  |  |  | 1.5 | 1.00 | 0.18 | **0.001** | **0.0001** | **<0.0001** | **<0.0001** |
|  |  |  | 2.5 | 1.00 | 0.58 | **0.04** | 0.08 | **0.02** | **0.01** |
|  |  |  | 5 | 1.00 | 0.15 | **0.0005** | **<0.0001** | **<0.0001** | **<0.0001** |
| LargeAreaHighGray  LevelEmphasis | texture | glszm | 0.5 | 0.95 | **0.0003** | **<0.0001** | **<0.0001** | **<0.0001** | **<0.0001** |
|  |  |  | 1 | 0.98 | **0.004** | **<0.0001** | **<0.0001** | **<0.0001** | **<0.0001** |
|  |  |  | 1.5 | 0.99 | 0.49 | **0.001** | **0.0004** | **0.0001** | **<0.0001** |
|  |  |  | 2.5 | 1.00 | 0.20 | 0.14 | **0.02** | **0.009** | **0.001** |
|  |  |  | 5 | 1.00 | 0.20 | 0.15 | 0.19 | 0.27 | 0.36 |
| LargeAreaLowGray  LevelEmphasis | texture | glszm | 0.5 | 0.98 | 0.09 | **0.002** | **0.007** | **0.04** | 0.44 |
|  |  |  | 1 | 1.00 | 0.80 | 0.19 | 0.21 | 0.13 | 0.18 |
|  |  |  | 1.5 | 1.00 | 0.86 | 0.09 | 0.12 | 0.18 | 0.10 |
|  |  |  | 2.5 | 0.99 | 0.13 | 0.07 | **0.02** | **0.02** | **0.007** |
|  |  |  | 5 | 1.00 | 0.11 | **0.01** | **0.007** | **0.004** | **0.003** |
| LowGrayLevelZone  Emphasis | texture | glszm | 0.5 | 0.85 | **0.047** | **<0.0001** | **<0.0001** | **<0.0001** | **<0.0001** |
|  |  |  | 1 | 0.92 | 0.15 | **0.002** | **0.0003** | **<0.0001** | **<0.0001** |
|  |  |  | 1.5 | 0.97 | 0.95 | 0.06 | **0.04** | 0.09 | **0.003** |
|  |  |  | 2.5 | 0.98 | 0.28 | 0.15 | 0.19 | 0.13 | **0.02** |
|  |  |  | 5 | 0.99 | 0.38 | 0.12 | **0.03** | 0.08 | 0.14 |
| SizeZoneNon  Uniformity | texture | glszm | 0.5 | 0.97 | **0.005** | **0.0002** | **0.0007** | **0.007** | 0.66 |
|  |  |  | 1 | 1.00 | **0.004** | **<0.0001** | **<0.0001** | **<0.0001** | **<0.0001** |
|  |  |  | 1.5 | 1.00 | **0.0001** | **<0.0001** | **<0.0001** | **<0.0001** | **<0.0001** |
|  |  |  | 2.5 | 1.00 | **<0.0001** | **<0.0001** | **<0.0001** | **<0.0001** | **<0.0001** |
|  |  |  | 5 | 1.00 | **<0.0001** | **<0.0001** | **<0.0001** | **<0.0001** | **<0.0001** |
| SizeZoneNon  UniformityNormalized | texture | glszm | 0.5 | **0.83*** | 0.50 | **0.03** | **0.0003** | **<0.0001** | **<0.0001** |
|  |  |  | 1 | 0.97 | **<0.0001** | **<0.0001** | **<0.0001** | **<0.0001** | **<0.0001** |
|  |  |  | 1.5 | 0.99 | **<0.0001** | **<0.0001** | **<0.0001** | **<0.0001** | **<0.0001** |
|  |  |  | 2.5 | 1.00 | **<0.0001** | **<0.0001** | **<0.0001** | **<0.0001** | **<0.0001** |
|  |  |  | 5 | 1.00 | **0.005** | **<0.0001** | **<0.0001** | **<0.0001** | **<0.0001** |
| SmallAreaEmphasis | texture | glszm | 0.5 | **0.83*** | 0.47 | **0.04** | **0.0009** | **<0.0001** | **<0.0001** |
|  |  |  | 1 | 0.97 | **<0.0001** | **<0.0001** | **<0.0001** | **<0.0001** | **<0.0001** |
|  |  |  | 1.5 | 0.99 | **<0.0001** | **<0.0001** | **<0.0001** | **<0.0001** | **<0.0001** |
|  |  |  | 2.5 | 1.00 | **0.0001** | **<0.0001** | **<0.0001** | **<0.0001** | **<0.0001** |
|  |  |  | 5 | 1.00 | **0.03** | **0.0002** | **<0.0001** | **<0.0001** | **<0.0001** |
| SmallAreaHighGray  LevelEmphasis | texture | glszm | 0.5 | 0.87 | **<0.0001** | **<0.0001** | **<0.0001** | **<0.0001** | **<0.0001** |
|  |  |  | 1 | 0.96 | **<0.0001** | **<0.0001** | **<0.0001** | **<0.0001** | **<0.0001** |
|  |  |  | 1.5 | 0.99 | **0.02** | **<0.0001** | **<0.0001** | **<0.0001** | **<0.0001** |
|  |  |  | 2.5 | 0.99 | 0.17 | **0.0010** | **0.0001** | **<0.0001** | **<0.0001** |
|  |  |  | 5 | 1.00 | 0.38 | 0.054 | **0.0003** | **0.0002** | **<0.0001** |
| SmallAreaLowGray  LevelEmphasis | texture | glszm | 0.5 | 0.85 | 0.19 | **0.004** | **<0.0001** | **<0.0001** | **<0.0001** |
|  |  |  | 1 | 0.92 | 0.46 | 0.08 | **0.04** | **0.04** | **0.002** |
|  |  |  | 1.5 | 0.97 | 0.94 | 0.61 | 0.62 | 0.10 | 0.38 |
|  |  |  | 2.5 | 0.98 | 0.52 | 0.21 | 0.23 | 0.10 | 0.07 |
|  |  |  | 5 | 0.97 | 0.28 | 0.49 | 0.58 | 0.61 | 0.66 |
| ZoneEntropy | texture | glszm | 0.5 | **0.58*** | **<0.0001** | **<0.0001** | **<0.0001** | **<0.0001** | **<0.0001** |
|  |  |  | 1 | 0.99 | 0.49 | 0.36 | 0.14 | 0.06 | **0.0005** |
|  |  |  | 1.5 | 1.00 | 0.07 | **<0.0001** | **<0.0001** | **<0.0001** | **<0.0001** |
|  |  |  | 2.5 | 1.00 | **0.05** | **<0.0001** | **<0.0001** | **<0.0001** | **<0.0001** |
|  |  |  | 5 | 1.00 | 0.10 | **0.04** | **0.005** | **0.001** | **0.002** |
| ZonePercentage | texture | glszm | 0.5 | 0.98 | **0.001** | **<0.0001** | **<0.0001** | **<0.0001** | **0.004** |
|  |  |  | 1 | 1.00 | 0.91 | 0.10 | **0.02** | **0.003** | **<0.0001** |
|  |  |  | 1.5 | 1.00 | **0.0006** | **<0.0001** | **<0.0001** | **<0.0001** | **<0.0001** |
|  |  |  | 2.5 | 1.00 | **<0.0001** | **<0.0001** | **<0.0001** | **<0.0001** | **<0.0001** |
|  |  |  | 5 | 1.00 | **0.0001** | **<0.0001** | **<0.0001** | **<0.0001** | **<0.0001** |
| ZoneVariance | texture | glszm | 0.5 | 0.97 | **0.001** | **<0.0001** | **<0.0001** | **<0.0001** | **<0.0001** |
|  |  |  | 1 | 0.99 | **0.01** | **<0.0001** | **<0.0001** | **<0.0001** | **<0.0001** |
|  |  |  | 1.5 | 1.00 | 0.18 | **0.001** | **0.0001** | **<0.0001** | **<0.0001** |
|  |  |  | 2.5 | 1.00 | 0.66 | 0.06 | 0.16 | 0.06 | 0.06 |
|  |  |  | 5 | 1.00 | 0.18 | **0.0008** | **<0.0001** | **<0.0001** | **<0.0001** |
| Coarseness_modified | texture | ngtdm1 | 0.5 | 0.98 | **0.03** | **<0.0001** | **<0.0001** | **<0.0001** | **<0.0001** |
|  |  |  | 1 | 0.98 | **<0.0001** | **<0.0001** | **<0.0001** | **<0.0001** | **<0.0001** |
|  |  |  | 1.5 | 0.99 | **0.0008** | **<0.0001** | **<0.0001** | **<0.0001** | **<0.0001** |
|  |  |  | 2.5 | 1.00 | **0.006** | **<0.0001** | **<0.0001** | **<0.0001** | **<0.0001** |
|  |  |  | 5 | 1.00 | **0.0005** | **<0.0001** | **<0.0001** | **<0.0001** | **<0.0001** |
| Complexity | texture | ngtdm1 | 0.5 | 0.90 | **0.0001** | **<0.0001** | **<0.0001** | **<0.0001** | **<0.0001** |
|  |  |  | 1 | 0.97 | **0.0001** | **<0.0001** | **<0.0001** | **<0.0001** | **<0.0001** |
|  |  |  | 1.5 | 0.99 | **0.0007** | **<0.0001** | **<0.0001** | **<0.0001** | **<0.0001** |
|  |  |  | 2.5 | 1.00 | **0.0040** | **<0.0001** | **<0.0001** | **<0.0001** | **<0.0001** |
|  |  |  | 5 | 1.00 | 0.46 | 0.07 | **0.0003** | **0.0001** | **0.0001** |
| Contrast | texture | ngtdm1 | 0.5 | 0.98 | **0.002** | **0.0002** | **<0.0001** | **0.0002** | **<0.0001** |
|  |  |  | 1 | 0.99 | 0.37 | 0.13 | 0.41 | 0.47 | 0.52 |
|  |  |  | 1.5 | 1.00 | 0.51 | 0.55 | 0.21 | 0.08 | **0.01** |
|  |  |  | 2.5 | 1.00 | 0.85 | 0.28 | **0.05** | **0.02** | **0.005** |
|  |  |  | 5 | 1.00 | 0.96 | 0.79 | 0.21 | 0.37 | 0.93 |
| Strength | texture | ngtdm1 | 0.5 | 0.94 | **<0.0001** | **<0.0001** | **<0.0001** | **<0.0001** | **<0.0001** |
|  |  |  | 1 | 0.98 | **<0.0001** | **<0.0001** | **<0.0001** | **<0.0001** | **<0.0001** |
|  |  |  | 1.5 | 0.99 | **0.009** | **<0.0001** | **<0.0001** | **<0.0001** | **<0.0001** |
|  |  |  | 2.5 | 1.00 | **0.02** | **0.0009** | **0.0004** | **<0.0001** | **<0.0001** |
|  |  |  | 5 | 1.00 | 0.81 | 0.34 | **0.006** | **0.004** | **0.003** |
| Coarseness_modified | texture | ngtdm4 | 0.5 | 0.98 | 0.81 | 0.72 | 0.94 | 0.67 | 0.10 |
|  |  |  | 1 | 0.99 | 0.15 | **0.007** | **0.0008** | **0.0002** | **<0.0001** |
|  |  |  | 1.5 | 1.00 | **0.02** | **<0.0001** | **<0.0001** | **<0.0001** | **<0.0001** |
|  |  |  | 2.5 | 1.00 | 0.58 | 0.46 | 0.37 | 0.73 | 0.65 |
|  |  |  | 5 | 1.00 | **<0.0001** | **<0.0001** | **<0.0001** | **<0.0001** | **<0.0001** |
| Complexity | texture | ngtdm4 | 0.5 | 0.91 | **<0.0001** | **<0.0001** | **<0.0001** | **<0.0001** | **<0.0001** |
|  |  |  | 1 | 0.97 | **<0.0001** | **<0.0001** | **<0.0001** | **<0.0001** | **<0.0001** |
|  |  |  | 1.5 | 0.99 | **0.001** | **<0.0001** | **<0.0001** | **<0.0001** | **<0.0001** |
|  |  |  | 2.5 | 1.00 | **0.002** | **<0.0001** | **<0.0001** | **<0.0001** | **<0.0001** |
|  |  |  | 5 | 1.00 | 0.43 | **0.05** | **0.0001** | **<0.0001** | **<0.0001** |
| Contrast | texture | ngtdm4 | 0.5 | 0.99 | **0.001** | **0.0003** | **<0.0001** | **0.0009** | **<0.0001** |
|  |  |  | 1 | 1.00 | 0.22 | 0.21 | 0.73 | 0.95 | 0.78 |
|  |  |  | 1.5 | 1.00 | 0.53 | 0.61 | 0.33 | 0.15 | **0.04** |
|  |  |  | 2.5 | 1.00 | 0.7842 | 0.19 | **0.03** | **0.007** | **0.0005** |
|  |  |  | 5 | 1.00 | 0.58 | 0.41 | 0.94 | 0.65 | 0.09 |
| Strength | texture | ngtdm4 | 0.5 | 0.93 | **<0.0001** | **<0.0001** | **<0.0001** | **<0.0001** | **<0.0001** |
|  |  |  | 1 | 0.97 | **<0.0001** | **<0.0001** | **<0.0001** | **<0.0001** | **<0.0001** |
|  |  |  | 1.5 | 0.99 | **0.007** | **<0.0001** | **<0.0001** | **<0.0001** | **<0.0001** |
|  |  |  | 2.5 | 1.00 | **0.05** | **0.002** | **0.001** | **0.0001** | **<0.0001** |
|  |  |  | 5 | 1.00 | 0.87 | 0.45 | **0.02** | **0.01** | **0.03** |
| Coarseness_modified | texture | ngtdm7 | 0.5 | 0.98 | 0.80 | 0.34 | 0.10 | **0.01** | **<0.0001** |
|  |  |  | 1 | 1.00 | **0.01** | **<0.0001** | **<0.0001** | **<0.0001** | **<0.0001** |
|  |  |  | 1.5 | 1.00 | **0.01** | **<0.0001** | **<0.0001** | **<0.0001** | **<0.0001** |
|  |  |  | 2.5 | 1.00 | 0.92 | 0.59 | 0.41 | 0.14 | **0.05** |
|  |  |  | 5 | 1.00 | **0.0006** | **<0.0001** | **<0.0001** | **<0.0001** | **<0.0001** |
| Complexity | texture | ngtdm7 | 0.5 | 0.91 | **<0.0001** | **<0.0001** | **<0.0001** | **<0.0001** | **<0.0001** |
|  |  |  | 1 | 0.97 | **<0.0001** | **<0.0001** | **<0.0001** | **<0.0001** | **<0.0001** |
|  |  |  | 1.5 | 0.99 | **0.0006** | **<0.0001** | **<0.0001** | **<0.0001** | **<0.0001** |
|  |  |  | 2.5 | 1.00 | **0.002** | **<0.0001** | **<0.0001** | **<0.0001** | **<0.0001** |
|  |  |  | 5 | 1.00 | 0.42 | **0.04** | **<0.0001** | **<0.0001** | **<0.0001** |
| Contrast | texture | ngtdm7 | 0.5 | 0.99 | **0.001** | **0.0005** | **0.0001** | **0.001** | **<0.0001** |
|  |  |  | 1 | 1.00 | 0.20 | 0.26 | 0.83 | 0.95 | 0.66 |
|  |  |  | 1.5 | 1.00 | 0.72 | 0.93 | 0.62 | 0.41 | 0.24 |
|  |  |  | 2.5 | 1.00 | 0.97 | 0.32 | 0.07 | **0.03** | **0.007** |
|  |  |  | 5 | 1.00 | 0.66 | 0.50 | 0.84 | 0.77 | 0.14 |
| Strength | texture | ngtdm7 | 0.5 | 0.93 | **<0.0001** | **<0.0001** | **<0.0001** | **<0.0001** | **<0.0001** |
|  |  |  | 1 | 0.97 | **<0.0001** | **<0.0001** | **<0.0001** | **<0.0001** | **<0.0001** |
|  |  |  | 1.5 | 0.99 | **0.008** | **<0.0001** | **<0.0001** | **<0.0001** | **<0.0001** |
|  |  |  | 2.5 | 1.00 | **0.05** | **0.001** | **0.0006** | **<0.0001** | **<0.0001** |
|  |  |  | 5 | 1.00 | 0.84 | 0.42 | **0.01** | **0.01** | **0.02** |

Notes: Significant FDR-adjusted p values for the multivariate model are in bold. OCCC < 0.85 are in bold and marked with an asterisk. Reference algorithm for mixed model analysis=FBP.

**Table S6. Percentage of features falling in each of the 4 groups, for the IR50 and the IR80 reconstructions. The results reported in this table refer to the sub-analysis performed on IR40, IR50, IR60 and IR80 (taking IR40 for comparison), similarly to Table 4 for the complete analysis. The percentage for the original images is evaluated excluding the features of the shape category.**

| **Image type** | **Image subtype** | **GROUP 1** | | **GROUP 2** | | **GROUP 3** | | **GROUP 4** | |
| --- | --- | --- | --- | --- | --- | --- | --- | --- | --- |
|  |  | **IR50** | **IR80** | **IR50** | **IR80** | **IR50** | **IR80** | **IR50** | **IR80** |
| **original** | **All**  (shape excluded) | **75.7** | **87.9** | **20** | **7.8** | **4.3** | **4.3** | **0.0** | **0.0** |
| **Wavelet** | **All** | **78.6** | **90.2** | **17.5** | **5.9** | **3.2** | **3.9** | **0.7** | **0.0** |
|  | LH | 19.6 | 22.7 | 4.5 | 1.4 | 0.9 | 0.9 | 0.0 | 0.0 |
|  | HL | 18.0 | 22.7 | 6.3 | 1.6 | 0.7 | 0.7 | 0.0 | 0.0 |
|  | HH | 20.4 | 22.7 | 3.2 | 0.9 | 1.4 | 1.4 | 0.0 | 0.0 |
|  | LL | 20.5 | 22.1 | 3.6 | 2.0 | 0.2 | 0.9 | 0.7 | 0.0 |
| **LoG** | **All** | **59.3** | **86.0** | **39.8** | **13.2** | **0.9** | **0.9** | **0.0** | **0.0** |
|  | 0.5 mm | 14.0 | 18.0 | 5.1 | 1.1 | 0.9 | 0.9 | 0.0 | 0.0 |
|  | 1.0 mm | 13.9 | 18.4 | 6.1 | 1.6 | 0.0 | 0.0 | 0.0 | 0.0 |
|  | 1.5 mm | 11.6 | 17.7 | 8.4 | 2.3 | 0.0 | 0.0 | 0.0 | 0.0 |
|  | 2.5 mm | 9.7 | 17.1 | 10.3 | 2.9 | 0.0 | 0.0 | 0.0 | 0.0 |
|  | 5.0 mm | 10.1 | 14.7 | 9.9 | 5.3 | 0.0 | 0.0 | 0.0 | 0.0 |


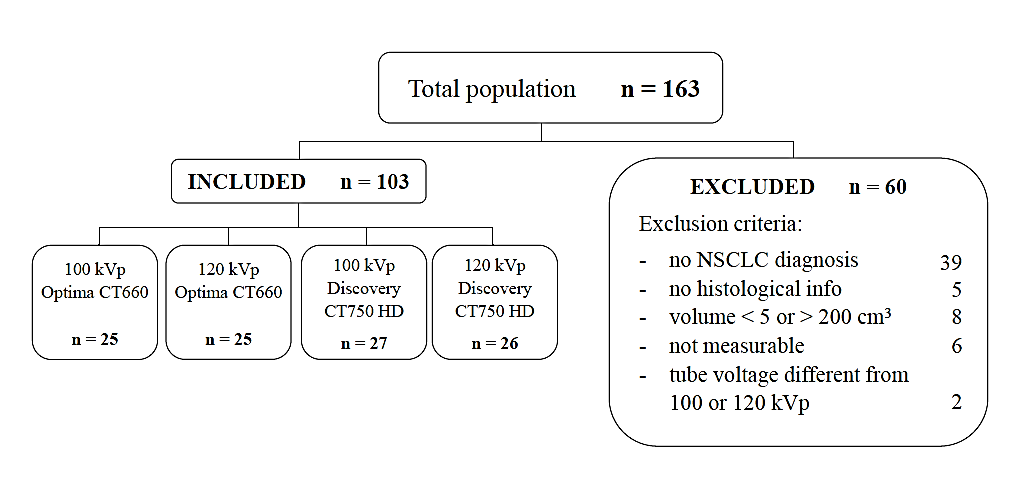
**Figure S1. Study flowchart with exclusion criteria.**

**Figure S2. Overall Concordance Correlation Coefficient (OCCC) for concordance between different algorithms for the sub-analysis** **of the blending levels mostly used in our clinical setting** **(IR40, IR50, IR60 and IR80). The OCCC is plotted within each subtype of feature and for feature extracted from the original images (a), and the Wavelet (b) and LoG-filtered (c) images.**

**(a)**

**
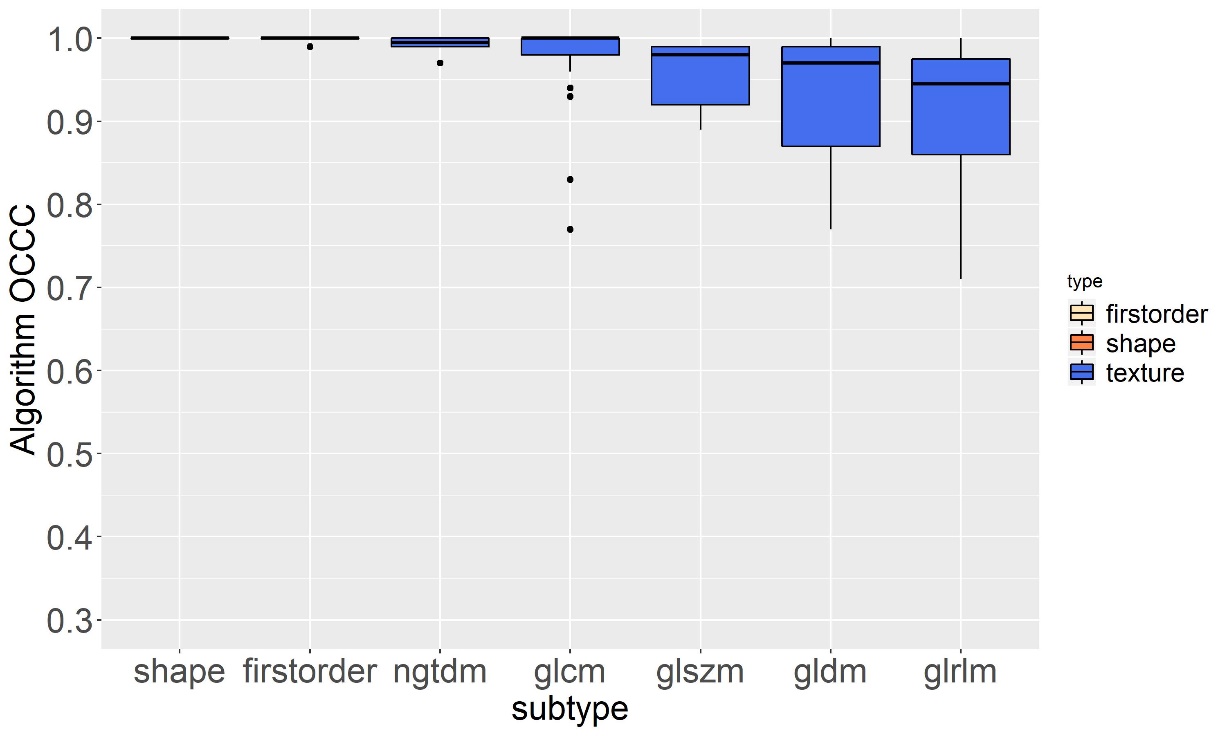
**

**(b)**

**
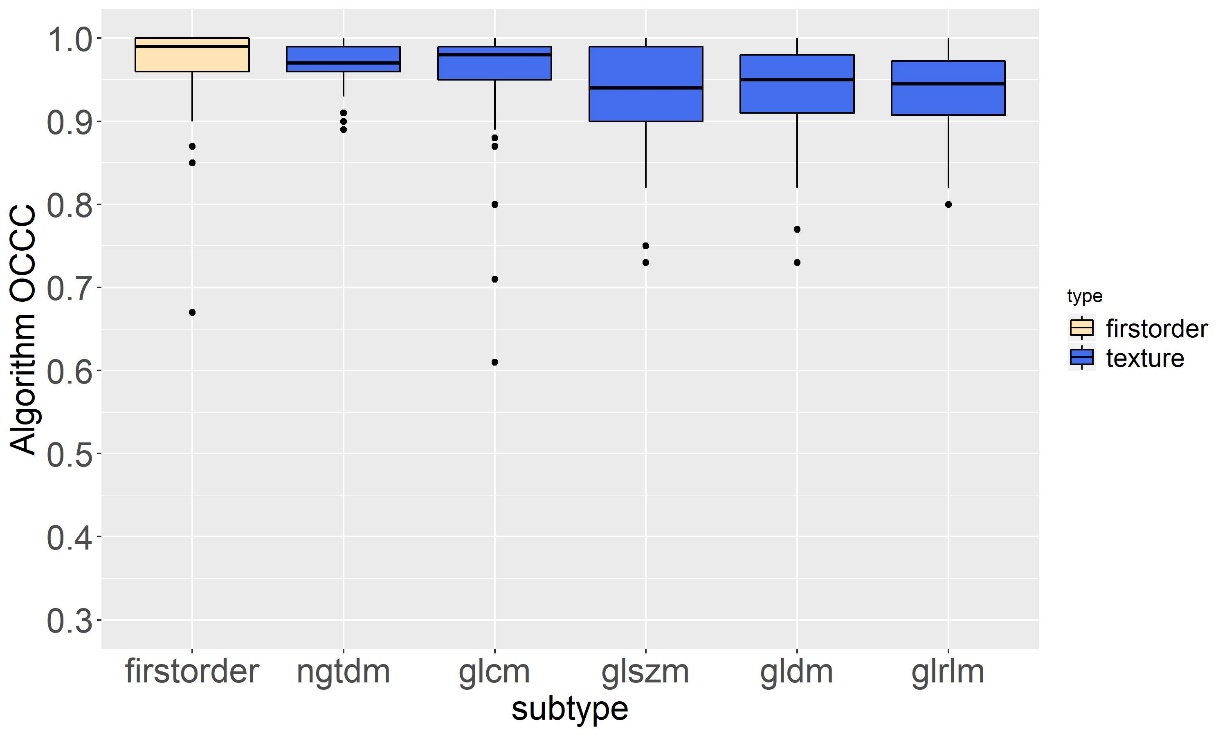
**

**(c)**

**
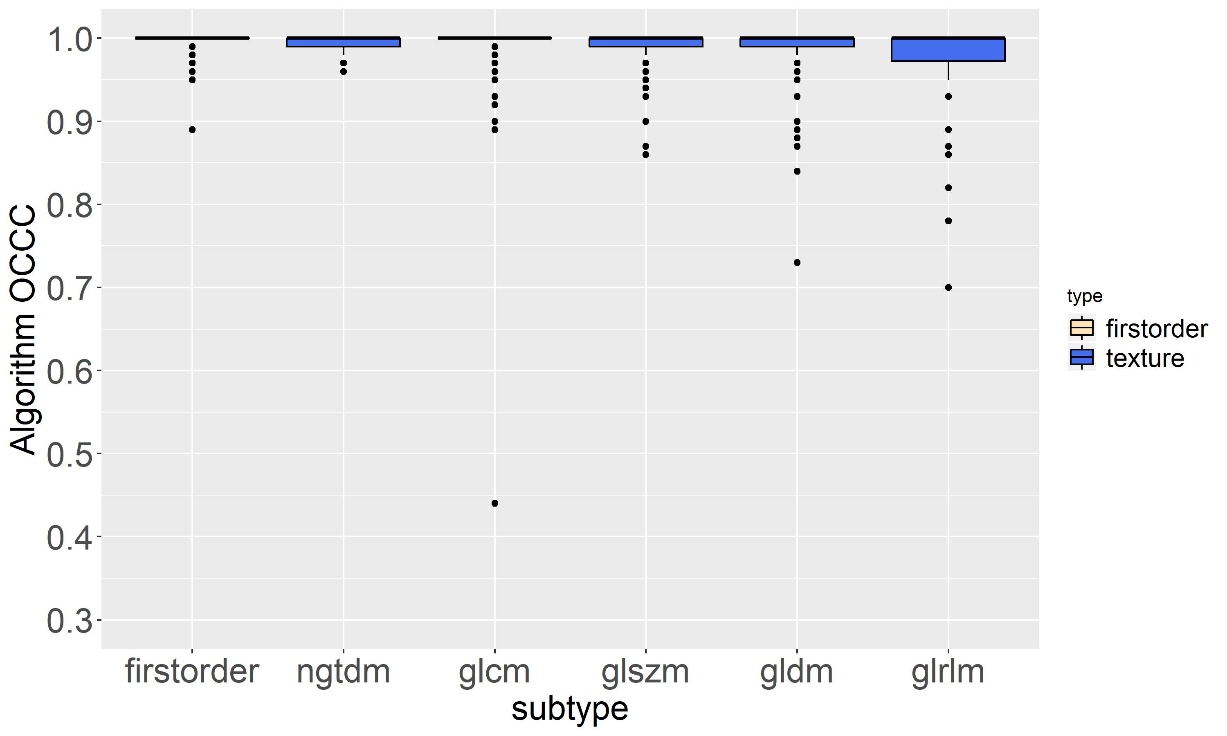
**

**Figure S3. Heatmap representing the number of features significantly different for paired comparisons of reconstruction algorithm strength (Wilcoxon signed rank-test), for the original images.** Shape feature excluded.

|  | **FBP** | **IR20** | **IR40** | **IR50** | **IR60** | **IR80** |
| --- | --- | --- | --- | --- | --- | --- |
| **FBP** | 0 | 121 | 124 | 124 | 127 | 128 |
| **IR20** | 121 | 0 | 120 | 122 | 127 | 127 |
| **IR40** | 124 | 120 | 0 | 117 | 123 | 127 |
| **IR50** | 124 | 122 | 117 | 0 | 117 | 125 |
| **IR60** | 127 | 127 | 123 | 117 | 0 | 120 |
| **IR80** | 128 | 127 | 127 | 125 | 120 | 0 |

**Figure S4. Box plot representing the distribution of the radiomic features (a) *original_glrlm_RunVariance* and (b) modified *original_glrlm_RunVariance,*** **according to algorithm.** The modified *original_glrlm_RunVariance* was obtained after rescaling, using the correspondent coefficients for each algorithm obtained in the mixed model. Minimum and maximum are depicted by whiskers, the box signifies the upper and lower quartiles, the median and the mean are represented, respectively by a line and a small rhombus within the box**.**

**(a)**

**
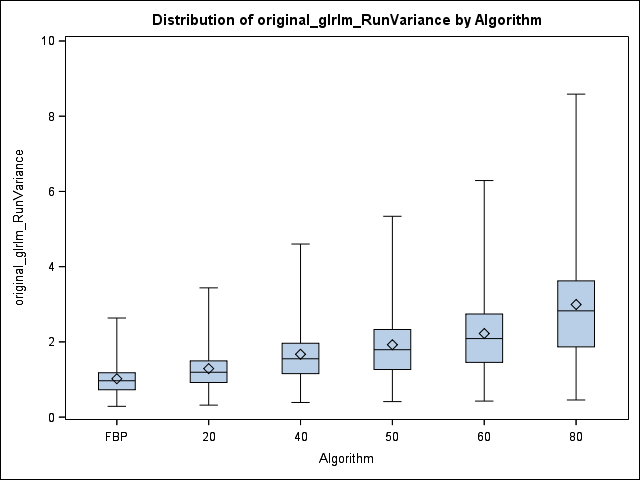
**

**(b)**

**
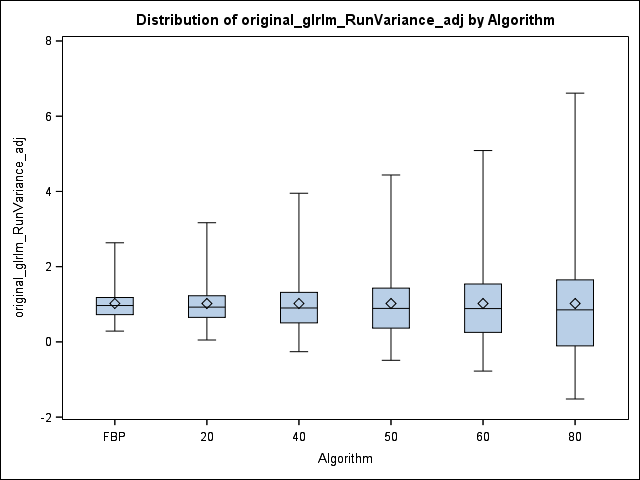
**
